# COPD patients with chronic bronchitis and higher sputum eosinophil counts show increased type‐2 and PDE4 gene expression in sputum

**DOI:** 10.1111/jcmm.16146

**Published:** 2020-12-09

**Authors:** Dave Singh, Michele Bassi, Deborah Balzano, Germano Lucci, Aida Emirova, Marie Anna Nandeuil, Gera Jellema, Ebenezer K. Afolabi, Brian Leaker, Oliver Kornmann, Kai Michael Beeh, Henrik Watz, Mirco Govoni

**Affiliations:** ^1^ Medicines Evaluation Unit The University of Manchester Manchester University NHS Foundation Hospital Trust Manchester UK; ^2^ Global Clinical Development Chiesi, Parma Italy; ^3^ Almac Diagnostics Craigavon UK; ^4^ The Heart Lung Centre London UK; ^5^ IKF Pneumologie Frankfurt Clinical Research Centre Respiratory Diseases Frankfurt Germany; ^6^ Insaf Respiratory Research Institute Wiesbaden Germany; ^7^ Pulmonary Research Institute at Lung Clinic Grosshansdorf Airway Research Center North (ARCN) Member of the German Center for Lung Research (DZL) Grosshansdorf Germany

**Keywords:** chronic obstructive pulmonary disease, eosinophilic inflammation, phosphodiesterase 4 inhibitors

## Abstract

Chronic obstructive pulmonary disease (COPD) patients with higher eosinophil counts are associated with increased clinical response to phosphodiesterase‐4‐inhibitors (PDE4i). However, the underlying inflammatory mechanisms associated with this increased response is not yet elucidated. This post hoc analysis focused on sputum gene expression in patients with chronic bronchitis who underwent 32‐day treatment with two doses of the inhaled PDE4i CHF6001 (tanimilast) or placebo on top of triple therapy. Biological characterization and treatment effects were assessed between patients with different sputum eosinophil levels (eosinophil^high^ ≥ 3%; eosinophil^low^ < 3%) at baseline (primary samples) or at the end of the treatment of the placebo arm (validation samples). Forty‐one genes were differentially expressed in primary samples (p‐adjusted for false discovery rate < 0.05); all up‐regulated in eosinophil^high^ patients and functionally enriched for type‐2 and PDE4 inflammatory processes. Eleven out of nineteen genes having immune system biological processes annotations including IL5RA, ALOX15, IL1RL1, CLC, GATA1 and PDE4D were replicated using validation samples. The expression of a number of these inflammatory mediators was reduced by tanimilast treatment, with greater effects observed in eosinophil^high^ patients. These findings suggest that type‐2 and PDE4 overexpression in COPD patients with higher sputum eosinophil counts contribute to the differential clinical response to PDE4i observed in previous clinical trials.

## 1 INTRODUCTION

Chronic obstructive pulmonary disease (COPD) patients with higher sputum or blood eosinophil counts have a greater response to inhaled corticosteroids (ICS),^1^, ^2^, ^3^ with blood eosinophil counts being able to predict the ICS effect on exacerbation prevention. Furthermore, post hoc analyses of randomized controlled trials (RCTs) involving COPD patients with chronic bronchitis who received the PDE4 inhibitor roflumilast or placebo in addition to maintenance ICS and long‐acting bronchodilators also demonstrated an association between higher blood eosinophil counts and greater effects of roflumilast on exacerbation prevention.^4^ The mechanisms responsible for these differential drug effects are unknown but may relate to an increased presence of type‐2 (T2) inflammation in COPD patients with higher blood eosinophil counts causing different responses to anti‐inflammatory drugs.^3^

CHF6001 (international non‐proprietary name (INN): tanimilast) is a novel PDE4 inhibitor currently in clinical development, specifically formulated as an extrafine formulation to be delivered by inhalation.^5^ PDE4s are a family of cAMP‐specific enzymes encoded by four genes (PDE4A, PDE4B, PDE4C and PDE4D) that are abundantly expressed in leucocytes. Tanimilast can inhibit all A–D isoforms with equal potency exerting a broad spectrum of anti‐inflammatory effects in almost all cells of the immune system.^6^ In preclinical studies and RCTs, tanimilast showed a potent topical anti‐inflammatory effect which was devoid of class‐related systemic adverse effects.^5^, ^6^, ^7^, ^8^, ^9^, ^10^ In recent post hoc analyses, it was shown that tanimilast significantly reduced the exacerbation rate in the subgroup of COPD patients with chronic bronchitis and eosinophil count ≥ 150cells/µl after 24 weeks of treatment.^10^ In a biomarker RCT conducted in COPD patients with chronic bronchitis receiving triple therapy (ICS/ long‐acting β_2_ agonist therapy (LABA) / long‐acting muscarinic antagonist (LAMA)), tanimilast showed clear anti‐inflammatory effects by modulating a range of airway biomarkers and inflammation pathways after 32 days of treatment.^8^, ^11^ Moreover, the ability of tanimilast to reduce sputum eosinophil counts was increased in patients with higher eosinophils levels in sputum (≥3%).^12^ These data are compatible with previous RCT results with roflumilast showing inhibition of sputum eosinophil counts accompanied by a reduction in bronchial mucosal eosinophil numbers.^13^

We have performed a post hoc analysis using samples from the tanimilast biomarker RCT.^8^ Samples obtained before randomization (primary samples) and at the end of the treatment of the placebo arm (validation samples) were used to stratify patients according to sputum eosinophil counts. A threshold of 3% was used to define a subset of patients associated with the phenotype of eosinophilic COPD, that is ‘eosinophil^high^’ (≥3%) versus ‘eosinophil^low^’ (<3%).^14^, ^15^, ^16^ Differential whole‐genome gene expression analysis was carried out in whole blood and sputum cells by microarray with the aim to characterize the underlying biology and to evaluate the effect of the treatment on the two groups of patients.

## 2 MATERIALS AND METHODS

### 2.1 Study objective and design

This post hoc analysis was conducted on samples from patients being treated with triple therapy who were randomized to one of the 32‐day treatment periods (800 or 1600 μg twice daily (BID); total daily doses of 1600 or 3200 μg or placebo) in a crossover study (ClinicalTrials.gov: NCT03004417), results of which have been previously reported.^8^ Samples obtained before receiving the investigational drug (at screening visit; baseline samples for primary analysis) and at the end of the placebo treatment (latest available collection on Day 20, 26 or 32; placebo samples for validation analysis) were used to stratify patients into two subgroups using a sputum eosinophil threshold of 3%, that is ‘eosinophil^high^’ (≥3%) and ‘eosinophil^low^’ (<3%). Microarray differential whole‐genome gene expression analysis between subgroups and the effect of the treatment on the identified significant genes both in the primary and validation analyses was carried out in whole blood and/or sputum cells.

Methods for the sputum and blood sample collection, and processing for the ribonucleic acid [RNA] assessments, extraction and amplification, sample profiling, microarray data quality control and pre‐processing have been previously reported.^11^ The raw data analysed in this publication have been deposited in NCBI's Gene Expression Omnibus and are accessible through GEO Series accession number GSE133513 (https://www.ncbi.nlm.nih.gov/geo/query/acc.cgi?acc=GSE133513).

### 2.2 Patients

Patients were male or female, ≥40 years of age, current or ex‐smokers with a smoking history ≥ 10 pack‐years, a diagnosis of COPD, post‐bronchodilator FEV_1_ ≥ 30% and < 70% predicted, ratio of FEV_1_ to forced vital capacity (FVC) <0.70, COPD Assessment Test (CAT) score ≥ 10, a history of chronic bronchitis (defined as chronic cough and sputum production for more than three months per year for at least two consecutive years), and treated with inhaled triple ICS/LABA/LAMA therapy for at least two months prior to enrolment. All patients provided written informed consent prior to any study‐related procedure. The key exclusion criteria were a moderate or severe COPD exacerbation within six weeks prior to entry or between screening and randomization, and the use of PDE4i within two months prior to entry.^8^ The clinical study was approved by independent ethics committees for each institution and was performed in accordance with the principles of the Declaration of Helsinki, and the International Conference on Harmonization notes for guidance on Good Clinical Practice (ICH/CPMP/135/95). The clinical study is registered on ClinicalTrials.gov (NCT03004417).

### 2.3 Processing and data analysis

Microarray preparation and data processing were described in detail in a previous manuscript.^11^ Briefly, samples were pre‐processed using Robust Multichip Algorithm (RMA). Probe‐level intensity measurements (CEL files) were background corrected, normalized and summarized as expression measurements using RMA. Pre‐processed data were filtered to remove control transcripts and any uninformative transcripts (lowly expressed, invariant probe sets). Differential expression analysis between subgroups was performed in R version 4.0 (R Core Team, Vienna, Austria, 2020). The eBayes algorithm of the Linear Models for Microarray Data (LIMMA) Bioconductor package v3.44.1,^17^ with Benjamini‐Hochberg multiple testing correction,^18^ was used to identify significant probe sets from filtered microarray data. All probe sets with *P*‐value adjusted for false discovery rate (pFDR)<0.05 were considered to produce lists of significant differentially expressed genes (DEGs) for each analysis. When multiple probe sets were associated with the same gene, the probe set with the lowest pFDR value was considered in the DEG list.

The treatment response for the DEGs identified in the differential expression analyses between subgroups was evaluated as change from baseline to the end of the treatment period (Day 20, 26 or 32, with the latest available sample used for the analyses) using an ANCOVA model with (subgroup), subject (within subgroup), period, treatment, (treatment‐by‐subgroup interaction) and baseline value as independent variables.

### 2.4 Hierarchical clustering and functional enrichment analysis

Hierarchical heatmap clustering was performed in R version 4.0. Differentially expressed probe set lists were annotated with gene identifiers using the latest annotation provided by Affymetrix for the Plus 2.0 array. To further understand the underlying biological significance of DEGs, g:Profiler^19^ was used for functional enrichment analysis to produce KEGG, Reactome and Wiki pathways as well as gene ontology (GO) functional annotation (biological process (BP), cellular component (CC) and molecular function (MF)). DEG lists were used as input. Only DEG lists comprising more than 30 unique genes were used as input for the analysis. Entities were ranked according to a statistically derived enrichment score and were adjusted for Benjamini‐Hochberg multiple testing (pFDR < 0.05).

### 2.5 Molecular interaction network analysis

The genes of interest were input into the PSICQUIC (Proteomics Standard Initiative proposed the Proteomics Standard Initiative Common QUery InterfaCe) web service to explore the molecular interaction based on Reactome,^20^ and IMEx databases.^21^ Network diagrams were drawn with Cytoscape version 3.8.0.

## 3 RESULTS

Fifty‐six patients (mean age 66; 40 males; 32 current smokers) were included in the analyses. Mean (SD) post‐bronchodilator predicted FEV_1_ was 49.7 (12.1) %, and COPD Assessment Test (CAT) score was 20.8 (5.9). The mean (SD) baseline blood eosinophil count was 252 (140) cells/µl or 3.3 (1.9) %. Mean (SD) and median (IQR) sputum percentage count were 3.7 (4.4) % and 2 (2.4) % for eosinophils and 83.5 (9.4) % and 83.9 (15.1) % for neutrophils, respectively.

45 163 and 44 355 probe sets in blood and sputum, respectively, corresponding to nearly the whole protein‐coding genome of 19 000 genes,^22^ were included in the analyses.

### 3.1 Primary analysis; baseline samples

At baseline, eighteen patients (32%) were eosinophil^high^ (≥3%; mean sputum eosinophils 8.8%), while thirty‐eight patients (68%) were eosinophil^low^ (mean 1.3%). Table 1 summarizes the clinical and sputum characteristics of the patients stratified by sputum eosinophil group. Eosinophil^high^ in comparison to eosinophil^low^ patients were characterized by higher proportion of males (78% vs 68%, respectively) and ex‐smokers (67% vs 32%, respectively), and lower levels of sputum neutrophils % (*P* = .007).

**Table 1:** Baseline demographics and disease characteristics

Parameter	Eosinophil^high^ (N = 18)	Eosinophil^low^ (N = 38)
Age (years), mean (SD)	66.6 (6.0)	65.9 (6.3)
Male gender, n (%)	14 (78)	26 (68)
Race, n (%)		
Caucasian	18 (100)	37 (97)
Asian	0 (0)	1 (3)
BMI (kg/m^2^), mean (SD)	27.2 (4.4)	25.3 (4.1)
Time since first COPD diagnosis (years), mean (range)	7.5 (1.8‐14.2)	10.2 (2.1‐21.0)
Smoking status at screening, n (%)		
Ex‐smoker	12 (67)	12 (32)
Current smoker	6 (33)	26 (68)
Post‐bronchodilator FEV_1_/FVC, mean (SD)	0.41 (0.10)	0.47 (0.10)
Post‐bronchodilator FEV_1_ (L), mean (SD)	1.31 (0.36)	1.53 (0.47)
Post‐bronchodilator FEV_1_ (% predicted), mean (SD)	45.1 (11.0)	51.9 (12.1)
COPD Assessment Test, mean (SD)	18.6 (4.7)	21.9 (6.2)
Serum C‐reactive protein (mg/L), mean (SD)	8.4 (14.7)	4.1 (5.0)
Serum interleukin‐6 (pg/mL), mean (SD)	2.6 (3.9)	1.7 (1.2)
Baseline Dyspnoea Index, mean (SD)	5.9 (1.8)	6.2 (1.9)
Sputum characteristics, mean (SD)		
Neutrophil cell count/weight (×10^6^/g)	2.97 (2.42)	6.05 (9.89)
Macrophage cell count/weight (×10^6^/g)	0.29 (0.28)	0.36 (0.31)
Eosinophil cell count/weight (×10^6^/g)	0.31 (0.35)	0.07 (0.10)
Lymphocyte cell count/weight (×10^6^/g)	0.01 (0.01)	0.01 (0.01)
Neutrophil %	78.6 (7.4)	85.8 (9.4)
Macrophage %	9.8 (6.5)	10.9 (8.4)
Eosinophil %	8.8 (4.6)	1.3 (0.8)
Lymphocyte %	0.2 (0.2)	0.2 (0.3)
Epithelial cells %	2.3 (3.6)	2.1 (3.4)

Abbreviations: BMI, body mass index; COPD, chronic obstructive pulmonary disease; FEV1, forced expiratory volume in 1 second; FVC, forced vital capacity; L, litre; SD, standard deviation.

**FIGURE 1:**
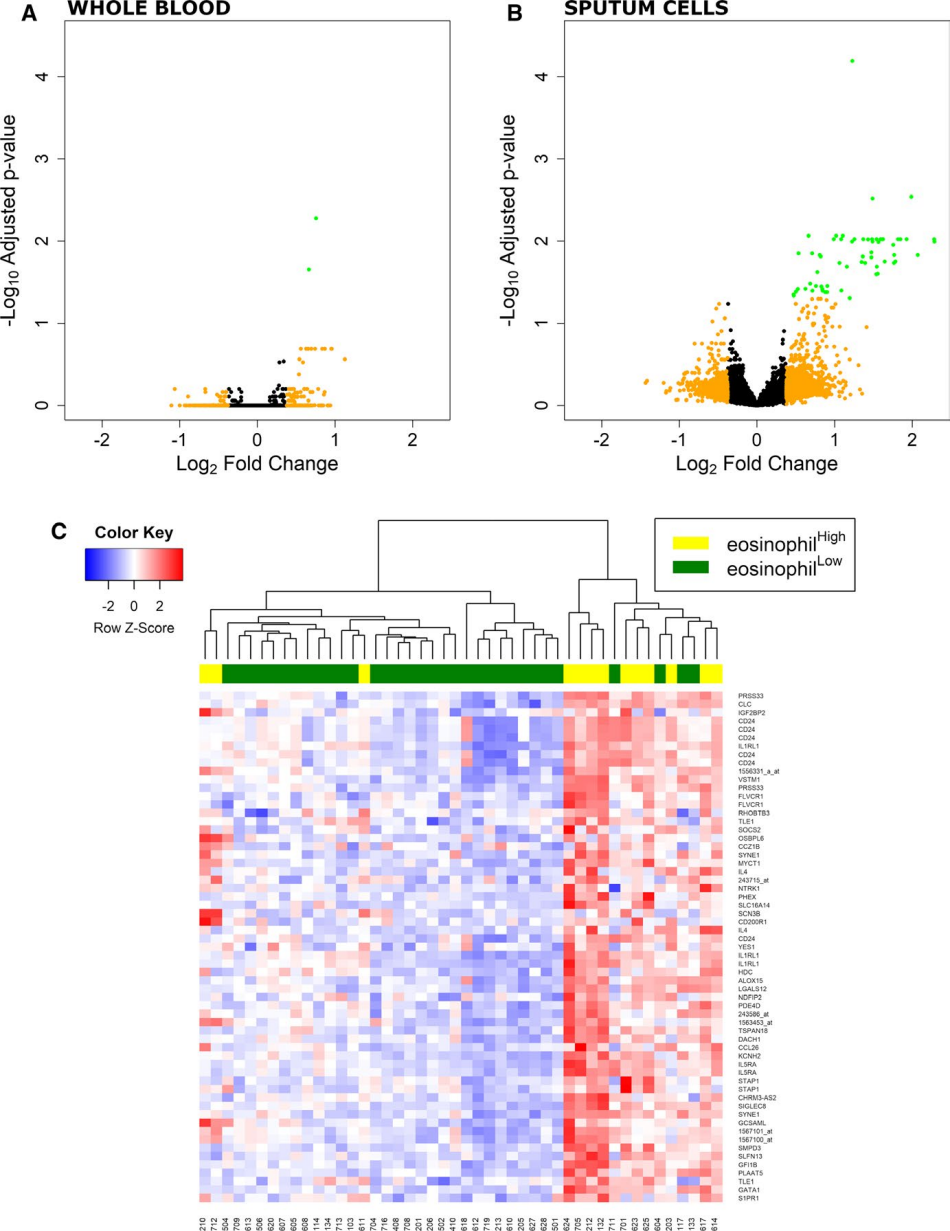
Differential expression analysis in A, whole blood and B, sputum cells between ‘eosinophil^high^’ and ‘eosinophil^low^’ patients (primary analysis; baseline samples). Volcano plot depicting all detected probe sets and coloured by fold change (FC) and adjusted *P*‐value (pFDR): green, FC >|1.3| and pFDR < 0.05; orange, FC>|1.3 |. C, Heatmap with z‐score representation and hierarchical clustering of patients based on values of the differentially (pFDR < 0.05) expressed probe sets in sputum cells (primary analysis; baseline samples)

**FIGURE 2:**
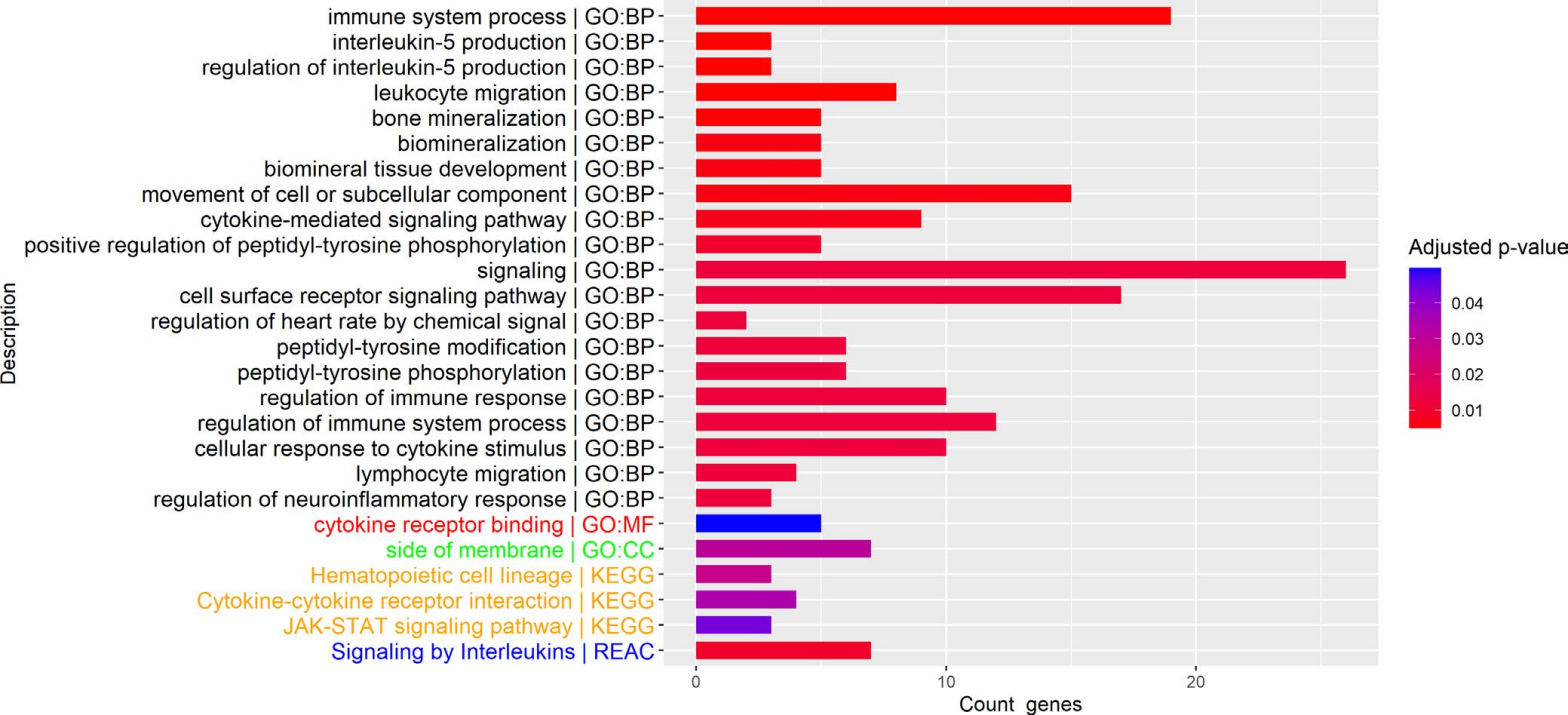
Top 20 gene ontology (GO) biological processes (black), GO molecular functions (red), GO cellular components (green), KEGG pathways (orange) and Reactome pathways (blue) identified by functional enrichment analysis of the significant (pFDR < 0.05) differentially expressed genes (DEGs) in sputum cells between eosinophil^high^ and eosinophil^low^ patients (primary analysis; baseline samples)

In blood, only 2 probe sets were significantly (pFDR < 0.05) up‐regulated in the eosinophil^high^ group, translating for peripheral myelin protein‐22 (PMP22) and phospholipase A acyltransferase‐5 (PLAAT5) (Figure 1A). In contrast, in sputum cells, there were 61 probe sets corresponding to 41 DEGs significantly differentially expressed (pFDR < 0.05) between the two groups (Figure 1B) with one gene in common with blood (PLAAT5). All probe sets were statistically significantly up‐regulated in the eosinophil^high^ compared to eosinophil^low^ population with fold change > |1.3| and pFDR < 0.05 (Table 2). Hierarchical heatmap clustering of the 61 significant probe sets highlighted one up‐regulated cluster enriched for eosinophil^high^ patients and one down‐regulated cluster enriched for eosinophil^low^ patients (Figure 1C). Functional analysis of the DEGs resulted in an enrichment (pFDR < 0.05) of 104 GO biological processes, 1 GO molecular function, 1 GO cellular component, 1 reactome pathway and 3 KEGG pathways. The most associated common terms were immune system processes, cytokine signalling, interleukin‐5 (IL5) production and cellular membrane components (Figure 2; Table S1). Overall, functional enrichment analysis and directionality of differential expression showed up‐regulation of inflammatory genes in sputum cells of eosinophil^high^ patients.

**Table 2:** Significant (pFDR < 0.05) differentially expressed genes (DEGs) in sputum cells between eosinophil^high^ and eosinophil^low^ patients (primary analysis; baseline samples and validation analysis; placebo samples)

Probe set ID	Gene symbol	Fold change primary analysis	pFDR primary analysis	Fold change validation analysis	pFDR validation analysis	Gene title
231947_at	MYCT1^b^	2.34	6.48E‐05	2.52	3.19E‐05	MYC target 1
208253_at	SIGLEC8 ^b^	3.97	2.90E‐03	3.92	1.22E‐03	Sialic acid–binding Ig‐like lectin 8
210036_s_at	KCNH2 ^b^	2.81	3.04E‐03	3.43	1.22E‐03	Potassium voltage‐gated channel subfamily H member 2
1553423_a_at	SLFN13^a^, ^b^	2.15	8.62E‐03	2.88	9.94E‐05	Schlafen family member 13
219695_at	SMPD3^a^, ^b^	2.03	8.62E‐03	2.03	2.10E‐03	Sphingomyelin phosphodiesterase 3
232027_at	SYNE1 ^b^	3.08	9.47E‐03	4.1	3.19E‐05	Spectrin repeat containing nuclear envelope protein 1
237403_at	GFI1B ^b^	2.12	9.47E‐03	2.43	3.19E‐05	Growth factor independent 1B transcriptional repressor
1552348_at	PRSS33^b^	3.8	9.47E‐03	5.44	3.08E‐04	Serine protease 33
235818_at	VSTM1^a^, ^b^	3.02	9.47E‐03	3.53	8.13E‐04	V‐set and transmembrane domain‐containing 1
1557733_a_at	CHRM3‐AS2 ^b^	3.52	9.47E‐03	4.06	1.40E‐03	CHRM3 antisense RNA 2
1554717_a_at	PDE4D^a^, ^b^	2.58	9.47E‐03	2.61	3.11E‐03	Phosphodiesterase 4D
1552908_at	GCSAML^a^	2.39	9.47E‐03	–	–	Germinal centre–associated signalling and motility like
223710_at	CCL26^a^	2.7	9.54E‐03	–	–	C‐C motif chemokine ligand 26
239229_at	PHEX ^b^	2.34	1.02E‐02	3.18	1.84E‐04	Phosphate‐regulating endopeptidase homolog X‐linked
211517_s_at	IL5RA^a^, ^b^	2.81	1.02E‐02	3.16	3.28E‐04	Interleukin‐5 receptor subunit alpha
242809_at	IL1RL1^a^, ^b^	4.88	1.02E‐02	6.2	7.30E‐04	Interleukin‐1 receptor‐like 1
220059_at	STAP1^a^	2.96	1.03E‐02	–	–	Signal‐transducing adaptor family member 1
207067_s_at	HDC	3.38	1.11E‐02	–	–	Histidine decarboxylase
210446_at	GATA1^a^, ^b^	1.64	1.41E‐02	1.58	1.16E‐03	GATA‐binding protein 1
1552875_a_at	CD200R1^a^	1.45	1.41E‐02	–	–	CD200 receptor 1
206207_at	CLC^a^, ^b^	4.2	1.48E‐02	5.17	1.46E‐02	Charcot‐Leyden crystal galectin
207538_at	IL4^a^	1.75	1.48E‐02	–	–	interleukin‐4
238029_s_at	SLC16A14 ^b^	2.57	1.55E‐02	4.3	3.19E‐05	solute carrier family 16 member 14
202976_s_at	RHOBTB3	1.77	1.55E‐02	–	–	Rho‐related BTB domain‐containing 3
207328_at	ALOX15^a^, ^b^	3.13	1.77E‐02	2.9	1.30E‐02	arachidonate 15‐lipoxygenase
208650_s_at	CD24^a^	3.44	1.77E‐02	–	–	CD24 molecule
223828_s_at	LGALS12 ^b^	3.4	1.85E‐02	3.86	1.22E‐03	Galectin 12
222906_at	FLVCR1^a^, ^b^	2.09	1.85E‐02	2.08	2.44E‐02	Feline leukaemia virus subgroup C cellular receptor 1
231050_at	PLAAT5 ^b^	2.23	2.05E‐02	2.31	1.23E‐03	Phospholipase A and acyltransferase 5
203222_s_at	TLE1	1.71	2.39E‐02	–	–	TLE family member 1, transcriptional corepressor
202933_s_at	YES1^a^	1.69	3.53E‐02	–	–	YES proto‐oncogene 1, Src family tyrosine kinase
204722_at	SCN3B	1.78	3.58E‐02	–	–	Sodium voltage‐gated channel beta subunit 3
218847_at	IGF2BP2	1.54	3.80E‐02	–	–	Insulin‐like growth factor 2 mRNA‐binding protein 2
227307_at	TSPAN18 ^b^	1.79	3.84E‐02	2.07	1.22E‐03	tetraspanin 18
215024_at	CCZ1B	1.44	3.84E‐02	‐	‐	CCZ1 homolog B, vacuolar protein trafficking and biogenesis associated
205471_s_at	DACH1 ^b^	2.12	4.00E‐02	2.34	1.22E‐03	Dachshund family transcription factor 1
239401_at	S1PR1^a^, ^b^	1.64	4.04E‐02	1.54	3.31E‐02	Sphingosine‐1‐phosphate receptor 1
223805_at	OSBPL6	1.8	4.04E‐02	–	–	Oxysterol‐binding protein‐like 6
203373_at	SOCS2	1.84	4.19E‐02	–	–	Suppressor of cytokine signalling 2
224801_at	NDFIP2 ^b^	1.87	4.20E‐02	1.8	2.39E‐02	Nedd4 family‐interacting protein 2
208605_s_at	NTRK1^a^	1.39	4.49E‐02	–	–	Neurotrophic receptor tyrosine kinase 1
204947_at	E2F1	–	–	2.19	8.31E‐05	E2F transcription factor 1
225879_at	TSEN54	–	–	2.13	9.94E‐05	tRNA‐splicing endonuclease subunit 54
236563_at	RD3	–	–	2.51	1.84E‐04	Retinal degeneration 3, GUCY2D regulator
214523_at	CEBPE^a^	–	–	2.02	1.84E‐04	CCAAT‐enhancer‐binding protein epsilon
230285_at	SVIP^a^	–	–	1.68	2.32E‐03	Small VCP‐interacting protein
206361_at	PTGDR2^a^	–	–	1.73	2.68E‐03	Prostaglandin D2 receptor 2
201834_at	PRKAB1	–	–	1.69	4.67E‐03	Protein kinase AMP‐activated non‐catalytic subunit beta 1
204637_at	CGA	–	–	1.77	4.90E‐03	Glycoprotein hormones, alpha polypeptide
228677_s_at	RASAL3^a^	–	–	1.53	5.79E‐03	RAS protein activator like 3
230142_s_at	CIRBP	–	–	1.86	5.79E‐03	Cold‐inducible RNA‐binding protein
202766_s_at	FBN1^a^	–	–	2.91	6.21E‐03	Fibrillin 1
220813_at	CYSLTR2^a^	–	–	1.76	7.46E‐03	Cysteinyl leukotriene receptor 2
237142_at	PPARA	–	–	1.7	7.46E‐03	Peroxisome proliferator–activated receptor alpha
1556003_a_at	LOC101928817	–	–	2.22	1.35E‐02	Uncharacterized LOC101928817
202938_x_at	RRP7A	–	–	1.39	1.35E‐02	Ribosomal RNA‐processing 7 homolog A /// ribosomal RNA‐processing 7 homolog B, pseudogene
1553645_at	CCDC141	–	–	1.52	1.44E‐02	Coiled‐coil domain‐containing 141
205099_s_at	CCR1^a^	–	–	−1.67	1.46E‐02	C‐C motif chemokine receptor 1
220637_at	FAM124B	–	–	1.96	2.42E‐02	Family with sequence similarity 124 member B
239201_at	CDK15	–	–	1.43	2.70E‐02	Cyclin‐dependent kinase 15
235369_at	C14orf28	–	–	1.6	4.37E‐02	Chromosome 14 open reading frame 28
244189_at	TTC28‐AS1	–	–	1.64	4.60E‐02	TTC28 antisense RNA 1
208164_s_at	IL9R	–	–	1.43	4.63E‐02	Interleukin‐9 receptor
1554890_a_at	TIA1	–	–	1.46	4.88E‐02	TIA1 cytotoxic granule–associated RNA‐binding protein

^a^ Inflammatory genes annotated in immune system Gene Ontology (GO) biological processes. pFDR, adjusted *P*‐value.

^b^ Differentially expressed genes (DEGs) in common between the primary and validation analyses.

**FIGURE 3:**
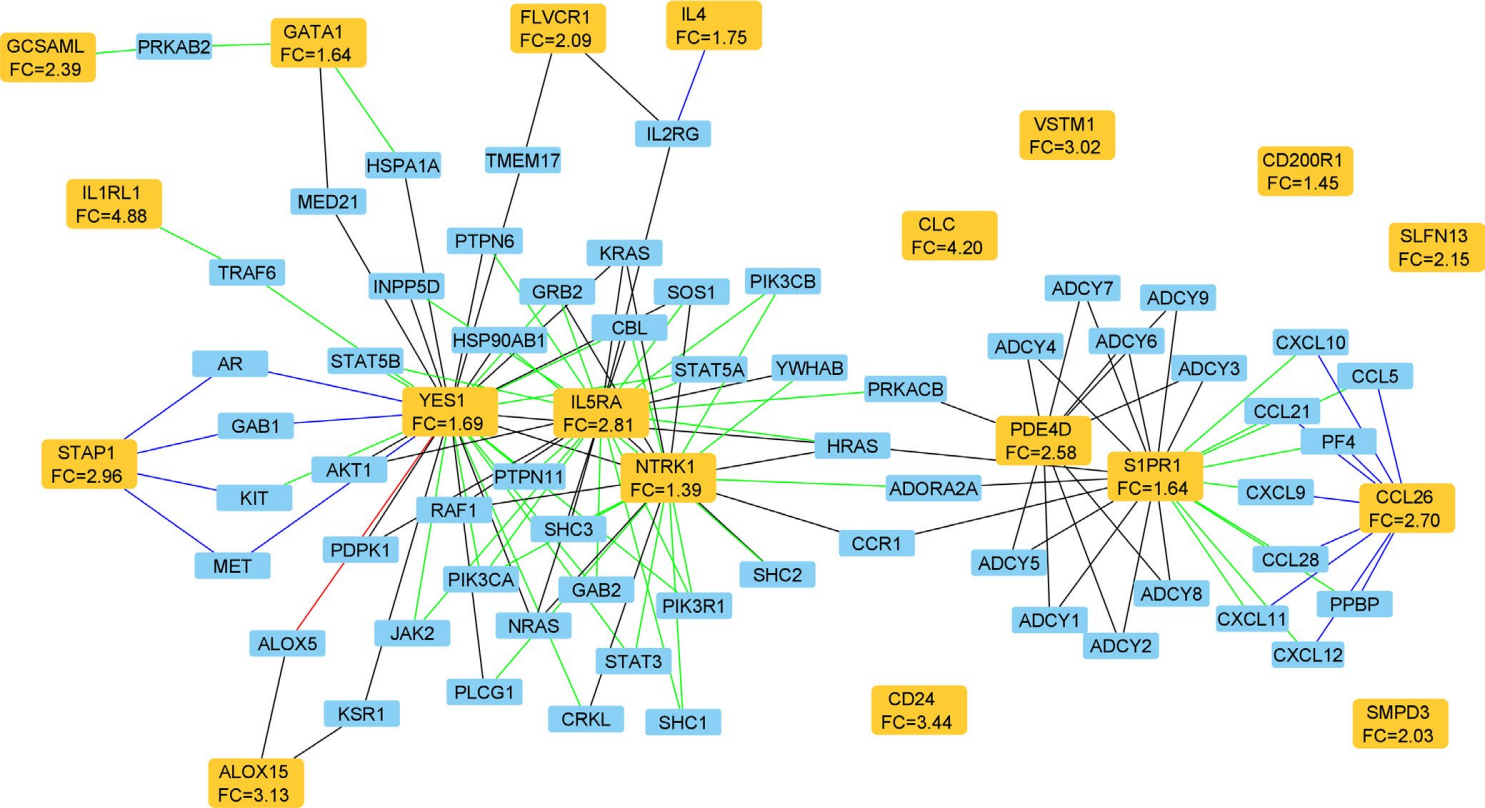
Inflammatory network molecular interaction analysis of the significant (pFDR < 0.05) differentially expressed genes (DEGs) associated with immune system GO biological processes (primary analysis; baseline samples; yellow nodes). Each node represents a single protein‐coding gene locus, edges represent the inferred type of association between nodes; light‐blue nodes, genes biologically associated with the differentially expressed genes (yellow nodes). Edges: a blue line, a direct interaction; a red line, phosphorylation reaction; a black line, biological association, which indicates interaction between molecules that may participate in formation of one physical complex, describing a set of molecules that are co‐purified in a single pull‐down or coimmunoprecipitation; a green line, biological physical association, indicating an interaction between molecules within the same physical complex, suggesting that the molecules are in close proximity but not necessarily in direct contact with each other. FC: fold change

Nineteen out the 41 DEGs had immune system GO biological process annotations,^23^ including interleukin‐5 receptor alpha (IL5RA), interleukin‐4 (IL4), chemokine (C‐C motif) ligand 26 (CCL26), arachidonate 15‐lipoxygenase (ALOX15), neurotrophic tyrosine kinase, receptor, type 1 (NTRK1), interleukin‐1 receptor‐like 1 (IL1RL1), CD24 molecule (CD24), charcot‐Leyden crystal galectin (CLC), GATA‐binding protein 1 (GATA1), feline leukaemia virus subgroup C cellular receptor 1 (FLVCR1), YES proto‐oncogene 1 (YES1), V‐set and transmembrane domain‐containing 1 (VSTM1), schlafen family member 13 (SLFN13), sphingosine‐1‐phosphate receptor 1 (S1PR1), sphingomyelin phosphodiesterase 3 (SMPD3) and the cAMP‐specific PDE4 isoform D (PDE4D) (Table 2; Table S1). Notably these inflammatory genes were all up‐regulated in the eosinophil^high^ group and were associated with T2 inflammation or PDE4 pathways (Figure 3). Furthermore, by exploring molecular network associations, major connections were found, with IL5RA, PDE4D, CCL26, S1PR1, NTRK1 and YES1 playing a central role involving the majority of interactions. Notably, PDE4 was found to be connected to the T2 inflammatory network via IL5RA, and S1PR1 with the cyclic AMP–dependent protein kinase A (PRKACB) and adenylate cyclase isoforms (ADCY) acting as bridging molecules in the network (Figure 3).

To explore if smoking status and gender could explain the biological differences observed between eosinophil^high^ and eosinophil^low^ patients, differential gene expression analysis was conducted between ex‐ and current smokers (43% and 57%, respectively) and male and female patients (71% and 29%, respectively). This analysis highlighted major biological differences in sputum caused by active smoking; 750 probe sets, corresponding to 497 DEGs, were significantly differentially expressed (pFDR < 0.05) in sputum, while no probe sets were significant in blood (Figure S1; Table S2). Only two genes (both up‐regulated in ex‐smokers) were in common with DEGs between eosinophil^high^ and eosinophil^low^ patients; IL1RL1 and the family member 1, transcriptional corepressor (TLE). Functional analysis of DEGs in sputum resulted in an enrichment (pFDR < 0.05) of 318 GO biological processes, 16 GO molecular functions, 25 GO cellular components, 2 KEGG and 12 Wiki pathways (Table S3). The most common terms associated with the top entities were response to chemical stimulus and cytokine activity.

Differential gene expression analysis between male and females in comparison to eosinophilic status did not show any overlapping significant probe set (Figure S2; Table S4; Table S5, Table S6).

### 3.2 Validation analysis; Placebo samples

Stratification using the available placebo sputum samples resulted in thirteen out of fifty patients (26%) being eosinophil^high^; 77% of whom maintained levels ≥ 3% from baseline.

Key results observed in the primary analysis using baseline samples were reproduced in this validation analysis using placebo samples. Specifically, 77 probe sets, corresponding to 48 DEGs, were significantly differentially expressed (pFDR < 0.05) between eosinophil^high^ and eosinophil^low^ patients in sputum cells (Table 2; Figure S3). Twenty‐five out of the 48 DEGs (52%) were in common with the primary analysis. From the 19 genes with immune system process annotations, 11 were common (61%); IL5RA, ALOX15, IL1RL1, CLC, GATA1, FLVCR1, VSTM1, SLFN13, S1PR1, SMPD3 and PDE4D.

Hierarchical heatmap clustering of the 77 significant probe sets highlighted one up‐regulated cluster enriched for eosinophil^high^ patients and one down‐regulated cluster enriched for eosinophil^low^ patients (Figure S4). Seventy‐six out of 77 probe sets were statistically significantly up‐regulated in the eosinophil^high^ compared to eosinophil^low^ population, all with fold change > |1.3| and pFDR < 0.05. Functional analysis of DEGs resulted in an enrichment (pFDR < 0.05) of GO biological processes and GO molecular functions associated with immune system processes, cytokine and interleukin‐5 (IL5) signalling (Table S7).

**FIGURE 4:**
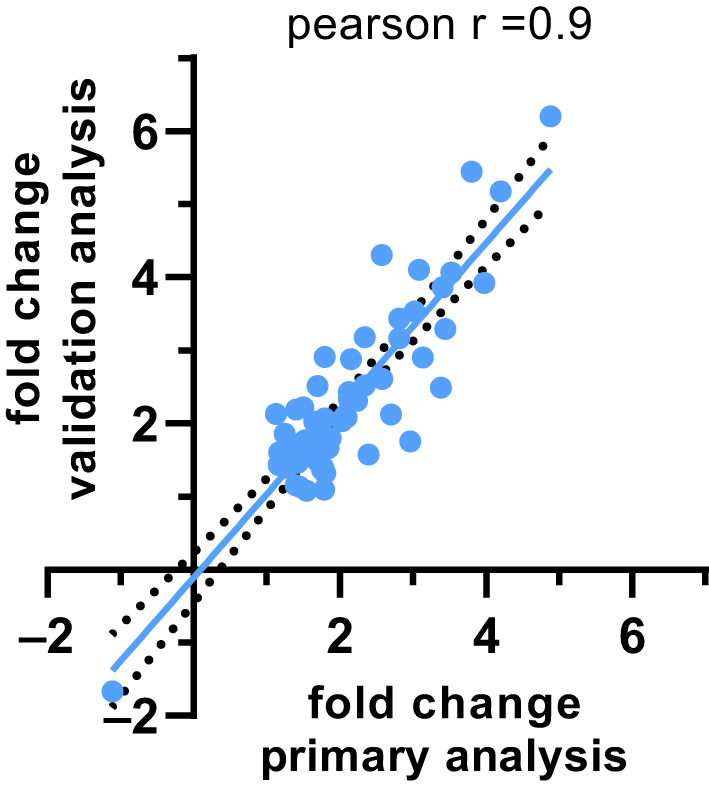
Correlation between fold change values of the primary and validation analyses for the significant (pFDR < 0.05) differentially expressed genes (DEGs) identified in the two analyses, with regression line and 95% confidence intervals

Considering the DEGs identified in the primary and validation analyses (41 and 48 DEGs, respectively, corresponding to 65 unique genes; Table 2), there was a strong correlation between the corresponding fold change values from the two data sets (Pearson‐r = 0.9, *P* < .0001; Figure 4; Table S8). In particular, three key T2 inflammatory DEGs identified in the validation analysis, namely cysteinyl leukotriene receptor 2 (CYSLTR2), prostaglandin D2 receptor 2 (PTGDR2) and CCAAT‐enhancer‐binding protein epsilon (CEBPE) when compared to the primary analysis showed fold changes of 1.76 vs 1.73, 1.73 vs 1.51 and 2.02 vs 1.65, respectively, and pFDR of 7.46E‐03 vs 5.04E‐02, 2.68E‐03 vs 3.47E‐01 and 1.84E‐04 vs 1.06E‐01, respectively (Table S8).

### 3.3 Tanimilast effect on gene expression in eosinophil^high/low^ patients

**FIGURE 5:**
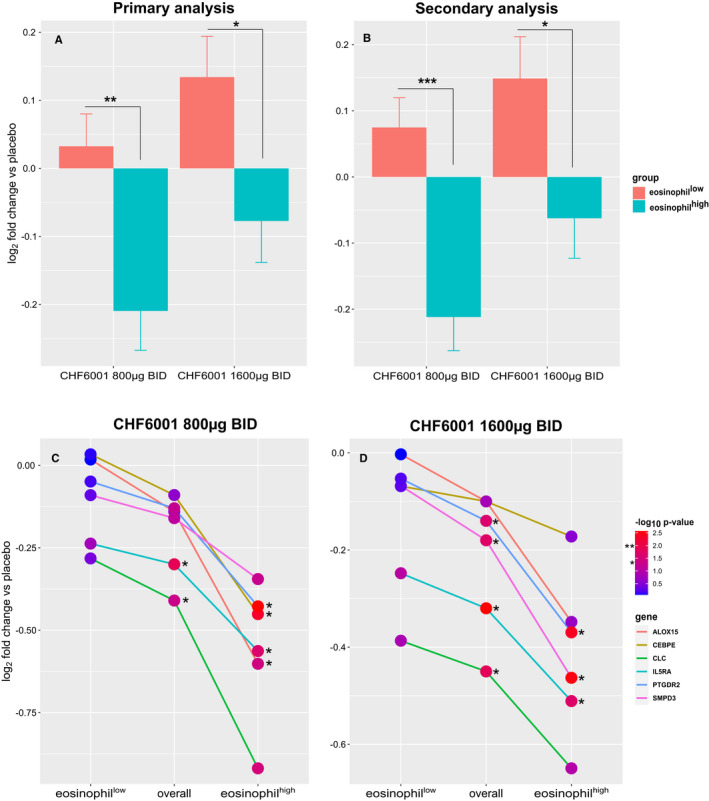
Fold change from pre‐dose to post‐dose expression versus placebo for the differentially expressed genes (DEGs) between eosinophil^high^ and eosinophil^low^ patients. A, B, mean (SEM) of 41 and 48 DEGs of the primary and validation analyses, respectively; Sidak's multiple comparison test ***P < .0001, **P < .001, *P < .05. C, D, DEGs with immune system processes annotations showing a fold change reduction versus placebo < −1.3 and *P < .05 for at least one of the two dose treatments, for tanimilast 800µg BID and tanimilast 1600µg BID, respectively

Analysis in sputum of the pre‐ to post‐dose gene expression fold change for tanimilast versus placebo treatments on the DEGs identified in the primary or in the validation analysis (Table 2) showed a greater treatment effects (causing reduced overall gene expression) for both doses of tanimilast in the eosinophil^high^ population (Figure 5A,B). In particular, among the inflammatory DEGs with immune system processes annotations (19 and 18 in the primary and validation analysis, respectively, corresponding to 26 unique genes; Table 2), IL5RA, CLC, ALOX15, SMPD3, PTGDR2, and CEBPE showed fold change reduction versus placebo < −1.3 and with *P* < .05 for at least one tanimilast dose (Figure 5C,D). Notably the expression of IL5RA was consistently and significantly reduced in the eosinophil^high^ population as well as in the overall population (without eosinophilic distinction) by both tanimilast doses.

## 4 DISCUSSION

The analysis presented here focused on sputum gene expression in COPD patients with chronic bronchitis being treated with inhaled triple therapy. Patients with higher sputum eosinophil counts had increased expression of T2‐ and PDE4‐related genes. Furthermore, the expression of a number of these genes was reduced by the extrafine inhaled PDE4 inhibitor tanimilast in the overall population, with greater effects observed in eosinophil^high^ patients. These findings suggest that T2 and PDE4 overexpression in COPD patients with higher sputum eosinophil counts contributes to the differential clinical response to PDE4 inhibitors observed in previous clinical trials.^4^, ^10^

The presence of T2 mediators in the airways of COPD patients has been previously shown in different studies measuring protein levels,^24^, ^25^, ^26^ and gene expression in bronchial epithelial brush.^27^, ^28^ We now build on these previous findings using an unbiased approach in sputum cells, specifically studying patients with chronic bronchitis already being treated with ICS and bronchodilators. A strength of this study is that primary and validation samples were obtained from the same patients, showing a number of DEGs in common and a strong correlation between the fold change values resulting from the two analyses.

The overexpression of specific genes in COPD patients with higher sputum eosinophil counts was modulated by the inhaled PDE4 inhibitor tanimilast. The genes for IL5RA, CLC, ALOX15, SMPD3, PTGDR2 and CEBPE showed a reduction (*P* < .05 and fold change <−1.3) of expression levels in comparison to placebo for at least one tanimilast dose. Notably the key mediator IL5RA was consistently and significantly reduced in the eosinophil^high^ population and the overall population by both tanimilast doses but with a more pronounced effect in the eosinophilic group. It has previously been reported that PDE4 inhibitors have a greater effect in COPD patients with higher eosinophil counts^4^, ^10^ and that PDE4 inhibition can reduce airways eosinophil numbers.^12^, ^13^ The results presented here demonstrate biological effects of PDE4 inhibition on inflammation processes associated with increased eosinophil counts in COPD patients, supporting these previous clinical observations.

The primary analysis showed 41 DEGs up‐regulated in eosinophil^high^ COPD patients, with 19 genes associated with inflammatory immune system biological processes. Eleven of these 19 genes were replicated in the validation analysis; IL5RA, ALOX15, IL1RL1, CLC, GATA1, FLVCR1, VSTM1, SLFN13, S1PR1, SMPD3 and PDE4D. These primary and validation results demonstrate up‐regulation of a specific T2‐ and PDE4‐related fingerprint that is associated with the phenotype of eosinophilic COPD.

Network analysis showed a set of genes (IL5RA, PDE4D, S1PR1, NTRK1 and YES1) playing central roles as interacting molecules within a network. Notable T2 cytokines and chemokines within this network were IL5RA which plays a key role in eosinophil differentiation, recruitment, activation and survival,^29^ IL4 which is secreted by T2 cells and is involved in the accumulation of eosinophils at sites of inflammation, B cell differentiation and T2 cytokine production,^29^ and CCL26 which acts as a ligand for C‐C motif chemokine receptor 3 (CCR3) which is expressed predominantly on eosinophils and mediates the chemotactic response to several chemokines.^29^ Furthermore, CCR3 receptors are strongly up‐regulated by the sphingolipid inflammatory mediator S1PR1 which is critically involved in eosinophils activation and recruitment.^30^

Other DEGs were also functionally linked to T2 inflammation,^31^, ^32^, ^33^, ^34^, ^35^, ^36^, ^37^ including ALOX15 and the tyrosine kinase receptor NTRK1 which play important roles in immune responses including the cellular response to interleukin‐13.^38^, ^39^ IL1RL1 is the receptor for interleukin‐33 (IL‐33) which acts as a selective chemoattractant of Th2 cells,^29^ and elicits IL5‐dependent eosinophilia.^40^ The signal transducer CD24 is an adhesion antigen expressed at the surface of eosinophils, B lymphocytes, T cells, dendritic cells and neutrophils. CD24 was recently shown to bind a variety of danger‐associated molecular patterns, such as high‐mobility group box protein‐1 (HMGB1), members of the heat‐shock‐protein (HSP) family and nucleolins.^41^ The transcription factor GATA1 is critically involved in T2 cell maturation, activation and granulopoiesis.^42^ Finally, CLC is a lysophospholipase expressed in eosinophils and basophils whose activity mediates extracellular cytotoxicity and inflammation.^43^

The expression of CLC and ALOX15 that we show here to be consistently associated with eosinophil counts and that is reduced by the effect of the PDE4 inhibitor tanimilast in sputum cells was also previously shown to be associated with T2‐high gene expression signature in bronchial epithelial brush.^27^, ^28^

In the validation analysis, we identified other two key receptors which are known pharmacological target of T2 inflammatory conditions, namely cysteinyl leukotriene 2 and prostaglandin D2.^44^, ^45^ Notably, assessment of the fold change values between the primary and validation analyses for the identified DEGs highlighted a strong correlation between the corresponding values from the two data sets.

We also identified a significant up‐regulation of the gene coding for the enzyme PDE4D in the eosinophilic population. This isoform, which is strongly inhibited by both roflumilast and tanimilast,^46^, ^47^ was shown to reduce the expression of adhesion molecules, airways reactivity and enhance muco‐ciliary clearance.^48^ The novel association found in the present analysis between PDE4D up‐regulation and eosinophilic inflammation was also supported by the network analysis of the inflammatory genes showing interactions between PDE4D and the T2 network via IL5RA and S1PR1 with the cyclic AMP–dependent protein kinase A (PRKACB) and adenylate cyclase isoforms (ADCY) acting as bridging molecules. Differently from sputum, the differential expression in blood did result in only two genes significantly up‐regulated in the eosinophil^high^ group of patients. Notably one of these genes, PLAAT5, which is involved in phospholipase A1/2 and acyltransferase activities was also significantly up‐regulated in sputum.

Our findings indicate that eosinophil^high^ COPD patients display a specific profile of T2‐related airway inflammation despite the concomitant use of ICS. In the control groups of two recent meta‐analyses of roflumilast and mepolizumab, in which COPD patients were using maintenance ICS and bronchodilators, there was an increase in exacerbations at higher blood eosinophil counts.^4^, ^49^ Overall, these findings suggest ongoing T2‐ and PDE4‐related inflammation associated with eosinophil numbers in COPD patients treated with ICS that may be targeted with PDE4i.

In our study population, eosinophil^high^ COPD patients were characterized by a higher proportion of males and ex‐smokers. These findings are in line with some other studies which showed a higher prevalence of males in COPD patients with higher eosinophil counts,^50^, ^51^, ^52^, ^53^ and a role of active smoking in decreasing the number of eosinophils in the airways with a possible impact on local T2 inflammation.^54^ Notably, we show that only one inflammatory gene, up‐regulated in ex‐smokers (IL1RL1), overlapped with the differentially expressed genes between eosinophil^high^ and eosinophil^low^ patients. This indicates that biological differences between patients characterized by different eosinophilic numbers in sputum cannot be explained by differences in gender and smoking status. Among the top twenty DEGs influenced by current smoking, AHRR (aryl‐hydrocarbon receptor repressor), CYP1A1 (cytochrome P450 family 1 subfamily A member 1) and CYP1B1 (cytochrome P450 family 1 subfamily B member 1) were also identified in previous studies aimed to assess the effect of smoking in lung tissue^55^ and in the oral mucosa.^56^

A threshold of 3% in sputum is widely used to identify a phenotype characterized by eosinophilic inflammation and comprises approximately 20‐40% of the whole COPD population.^14^, ^15^, ^16^ This proportion is in line with our observations that patients with sputum eosinophils ≥ 3% accounted for approximately one third of the whole study population. We recently showed, in this cohort of patients, that blood eosinophils predict sputum eosinophilia with an accuracy of approximately 80%.^12^ This association between blood and sputum eosinophils was also previously observed in other studies,^14^, ^15^ indicating that blood eosinophil counts can be a good surrogate marker of eosinophilic inflammation in sputum, which we show here is also associated with greater T2 inflammation.

We acknowledge that this analysis has some limitations. The limited sample size, in particular for the group of patients with higher sputum eosinophil counts, might have restricted the pool of genes significantly differential expressed preventing a complete biological differentiation. In addition, the use of complementary techniques (eg protein assessments) can add value to validate further gene expression findings.

In conclusion, recent studies showed that the effect of oral and inhaled PDE4 inhibitors on exacerbations in COPD patients with chronic bronchitis appears to be greater at higher blood eosinophil counts.^4^, ^10^ Furthermore, we recently showed that tanimilast significantly reduced sputum eosinophil numbers in the eosinophil^high^ group.^12^ These previous results coupled with our current data strongly suggest that differential responses to PDE4 inhibition may relate to an increased presence of features of T2 inflammation and PDE4‐related pathways in the eosinophilic COPD phenotype.

## CONFLICT OF INTERESTS

Dave Singh received personal fees from Chiesi during the conduct of this study. Outside the submitted work, he reports grants and personal fees from AstraZeneca, Boehringer Ingelheim, Chiesi, GlaxoSmithKline, Glenmark, Menarini, Mundipharma, Novartis, Pfizer, Pulmatrix, Theravance and Verona, and personal fees from Cipla, Genentech and Peptinnovate. Henrik Watz reports personal fees from Chiesi during the conduct of the study. Outside the submitted work, Dr Watz reports personal fees from Bayer, personal fees from GSK, personal fees from Boehringer Ingelheim, personal fees from Novartis, personal fees from AstraZeneca, personal fees from BerlinChemie and personal fees from Roche. Kai Michael Beeh is a full‐time employee of insaf Respiratory Research Institute. The institution has received compensation for services on advisory boards or consulting for Ablynx, Almirall, AstraZeneca, Berlin Chemie, Boehringer, Chiesi, Cytos, Mundipharma, Novartis, Pohl Boskamp and Zentiva. The institution has received compensation for speaker activities in scientific meetings supported by Almirall, AstraZeneca, Berlin Chemie, Boehringer, Cytos, ERT, GSK, Novartis, Pfizer, Pohl Boskamp and Takeda. The institution has received compensation for design and performance of clinical trials from Almirall, Altana/Nycomed, AstraZeneca, Boehringer, Cytos, GSK, Infinity, Medapharma, MSD, Mundipharma, Novartis, Parexel, Pearl Therapeutics, Pfizer, Revotar, Teva, Sterna and Zentiva. Oliver Kornmann's institution received fees from Chiesi for conducting this study as a participating site. Dr Kornmann reports personal fees as speaker or Advisory Board member from AstraZeneca, Boehringer Ingelheim, GlaxoSmithKline, Sanofi and Novartis. Brian Leaker, Brendan Colgan, Gera Jellema and Ebenezer K. Afolabi have nothing to disclose. Michele Bassi, Deborah Balzano, Germano Lucci, Aida Emirova, Marie Anna Nandeuil and Mirco Govoni are all employees of Chiesi, the sponsor of this trial.

## AUTHOR CONTRIBUTION

Dave Singh, Michele Bassi, Deborah Balzano and Mirco Govoni: Conception. Brian Leaker, Oliver Kornmann, Kai Michael Beeh, Henrik Watz and Dave Singh: Data acquisition. Michele Bassi, Deborah Balzano, Mirco Govoni, Ebenezer K. Afolabi and Gera Jellema: Data analysis. All authors interpreted the data and revised the manuscript for intellectual content and approved the submitted version.

## Supporting information

Supplementary Material

## Data Availability

Chiesi commits to sharing with qualified scientific and medical researchers, conducting legitimate research, patient‐level data, study‐level data, the clinical protocol and the full clinical study report of Chiesi Farmaceutici S.p.A.‐sponsored interventional clinical trials in patients for medicines and indications approved by the European Medicines Agency and/or the US Food and Drug Administration after *1st January 2015*, following the approval of any received research proposal and the signature of a Data Sharing Agreement. Chiesi provides access to clinical trial information consistently with the principle of safeguarding commercially confidential information and patient privacy. To date, the current study is out of scope of the Chiesi policy on Clinical Data Sharing. Other information on Chiesi's data sharing commitment, access and research request's approval process is available in the Clinical Trial Transparency section of http://www.chiesi.com/en/research‐and‐development/.
