# Supplementary material for: COPD patients with chronic bronchitis and higher sputum eosinophil counts show increased type‐2 and PDE4 gene expression in sputum
Source: J Cell Mol Med. 2020 Dec 9;25(2):905–18. doi: 10.1111/jcmm.16146 (PMC7812250; doi:10.1111/jcmm.16146)
Supplement: Supplementary file 1 — Supplementary Material [file JCMM-25-905-s001.docx]

**SUPPORTING INFORMATION**

***TABLE S1*** *Significant Gene Ontology (GO) biological processes (BP), GO molecular functions (MF), GO cellular components (CC), KEGG and REACTOME pathways identified by functional enrichment analysis of the significant (pFDR<0.05) differentially expressed genes (DEGs) in sputum cells between eosinophil^high^ and eosinophil^low^ patients* *(primary analysis; baseline samples). *Molecules from the Gene list that are annotated to the functional group. Abbreviation: pFDR, adjusted p-value*

| Entity | Description | | | pFDR | *Genes |
| --- | --- | --- | --- | --- | --- |
| GO:BP | immune system process | | 5.00E-03 | | SMPD3,IL4,SLFN13,PDE4D,VSTM1,GCSAML,IL1RL1,CCL26,IL5RA,STAP1,CD24,GATA1,CD200R1,CLC,ALOX15,FLVCR1,YES1,S1PR1,NTRK1 |
| GO:BP | interleukin-5 production | 5.00E-03 | | | PDE4D,IL1RL1,IL5RA |
| GO:BP | regulation of interleukin-5 production | 5.00E-03 | | | PDE4D,IL1RL1,IL5RA |
| GO:BP | leukocyte migration | 5.00E-03 | | | SMPD3,PDE4D,GCSAML,CCL26,STAP1,CD200R1,YES1,S1PR1 |
| GO:BP | bone mineralization | 5.00E-03 | | | SMPD3,PHEX,GATA1,ALOX15,S1PR1 |
| GO:BP | biomineralization | 6.26E-03 | | | SMPD3,PHEX,GATA1,ALOX15,S1PR1 |
| GO:BP | biomineral tissue development | 6.26E-03 | | | SMPD3,PHEX,GATA1,ALOX15,S1PR1 |
| GO:BP | movement of cell or subcellular component | 6.34E-03 | | | KCNH2,SMPD3,IL4,PDE4D,GCSAML,CCL26,STAP1,CD24,CD200R1,RHOBTB3,YES1,SCN3B,DACH1,S1PR1,NTRK1 |
| GO:BP | cytokine-mediated signaling pathway | 7.16E-03 | | | IL4,IL1RL1,CCL26,IL5RA,STAP1,CD24,ALOX15,S1PR1,SOCS2 |
| GO:BP | positive regulation of peptidyl-tyrosine phosphorylation | 9.84E-03 | | | IL4,STAP1,CD24,GATA1,YES1 |
| GO:BP | signaling | 1.24E-02 | | | SIGLEC8,KCNH2,SMPD3,IL4,PDE4D,VSTM1,GCSAML,PRSS33,IL1RL1,CCL26,PHEX,IL5RA,STAP1,CD24,GATA1,CD200R1,RHOBTB3,ALOX15,LGALS12,TLE1,YES1,SCN3B,S1PR1,SOCS2,NDFIP2,NTRK1 |
| GO:BP | cell surface receptor signaling pathway | 1.24E-02 | | | SMPD3,IL4,PDE4D,GCSAML,IL1RL1,CCL26,IL5RA,STAP1,CD24,GATA1,CD200R1,ALOX15,TLE1,YES1,S1PR1,SOCS2,NTRK1 |
| GO:BP | regulation of heart rate by chemical signal | 1.24E-02 | | | KCNH2,PDE4D |
| GO:BP | peptidyl-tyrosine modification | 1.24E-02 | | | IL4,STAP1,CD24,GATA1,YES1,NTRK1 |
| GO:BP | peptidyl-tyrosine phosphorylation | 1.24E-02 | | | IL4,STAP1,CD24,GATA1,YES1,NTRK1 |
| GO:BP | regulation of immune response | 1.24E-02 | | | IL4,PDE4D,GCSAML,IL1RL1,STAP1,CD24,CD200R1,CLC,ALOX15,YES1 |
| GO:BP | regulation of immune system process | 1.24E-02 | | | SMPD3,IL4,PDE4D,GCSAML,IL1RL1,STAP1,CD24,GATA1,CD200R1,CLC,ALOX15,YES1 |
| GO:BP | cellular response to cytokine stimulus | 1.24E-02 | | | SMPD3,IL4,IL1RL1,CCL26,IL5RA,STAP1,CD24,ALOX15,S1PR1,SOCS2 |
| GO:BP | lymphocyte migration | 1.24E-02 | | | GCSAML,CCL26,CD200R1,S1PR1 |
| GO:BP | regulation of neuroinflammatory response | 1.24E-02 | | | IL4,STAP1,CD200R1 |
| GO:BP | regulation of localization | 1.36E-02 | | | KCNH2,SMPD3,IL4,PDE4D,GCSAML,IL1RL1,CCL26,STAP1,CD200R1,ALOX15,YES1,SCN3B,DACH1,S1PR1,OSBPL6,NDFIP2 |
| GO:BP | locomotion | 1.36E-02 | | | SMPD3,IL4,PDE4D,GCSAML,CCL26,STAP1,CD24,CD200R1,RHOBTB3,YES1,DACH1,S1PR1,NTRK1 |
| GO:BP | regulation of cytokine production | 1.37E-02 | | | IL4,PDE4D,IL1RL1,IL5RA,CD24,CD200R1,CLC,IGF2BP2 |
| GO:BP | response to cytokine | 1.38E-02 | | | SMPD3,IL4,IL1RL1,CCL26,IL5RA,STAP1,CD24,ALOX15,S1PR1,SOCS2 |
| GO:BP | regulation of actin filament-based process | 1.44E-02 | | | PDE4D,CCL26,STAP1,RHOBTB3,ALOX15,S1PR1 |
| GO:BP | regulation of multicellular organismal process | 1.44E-02 | | | KCNH2,SMPD3,IL4,PDE4D,IL1RL1,IL5RA,STAP1,CD24,GATA1,CD200R1,CLC,FLVCR1,SCN3B,IGF2BP2,S1PR1,SOCS2,NTRK1 |
| GO:BP | actin filament-based process | 1.44E-02 | | | KCNH2,PDE4D,CCL26,STAP1,RHOBTB3,ALOX15,SCN3B,S1PR1 |
| GO:BP | regulation of peptidyl-tyrosine phosphorylation | 1.44E-02 | | | IL4,STAP1,CD24,GATA1,YES1 |
| GO:BP | sphingolipid mediated signaling pathway | 1.80E-02 | | | SMPD3,S1PR1 |
| GO:BP | negative regulation of macrophage migration | 1.80E-02 | | | STAP1,CD200R1 |
| GO:BP | multicellular organism growth | 1.83E-02 | | | SMPD3,PDE4D,FLVCR1,SOCS2 |
| GO:BP | cytokine production | 1.83E-02 | | | IL4,PDE4D,IL1RL1,IL5RA,CD24,CD200R1,CLC,IGF2BP2 |
| GO:BP | regulation of adaptive immune response | 1.91E-02 | | | IL4,IL1RL1,CLC,ALOX15 |
| GO:BP | cellular response to organic substance | 1.91E-02 | | | SMPD3,IL4,PDE4D,IL1RL1,CCL26,PHEX,IL5RA,STAP1,CD24,GATA1,ALOX15,YES1,S1PR1,SOCS2,NTRK1 |
| GO:BP | positive regulation of interleukin-5 production | 1.91E-02 | | | PDE4D,IL1RL1 |
| GO:BP | cell migration | 1.99E-02 | | | SMPD3,IL4,PDE4D,GCSAML,CCL26,STAP1,CD24,CD200R1,YES1,DACH1,S1PR1 |
| GO:BP | T cell migration | 1.99E-02 | | | CCL26,CD200R1,S1PR1 |
| GO:BP | regulation of transport | 2.17E-02 | | | KCNH2,SMPD3,IL4,PDE4D,IL1RL1,STAP1,CD200R1,ALOX15,YES1,SCN3B,OSBPL6,NDFIP2 |
| GO:BP | regulation of cellular component movement | 2.17E-02 | | | SMPD3,IL4,PDE4D,GCSAML,CCL26,STAP1,CD200R1,DACH1,S1PR1 |
| GO:BP | regulation of phosphorylation | 2.17E-02 | | | SMPD3,IL4,PDE4D,CCL26,STAP1,CD24,GATA1,ALOX15,YES1,SOCS2,NTRK1 |
| GO:BP | regulation of phagocytosis, engulfment | 2.17E-02 | | | STAP1,ALOX15 |
| GO:BP | cell communication | 2.17E-02 | | | SIGLEC8,SMPD3,IL4,PDE4D,VSTM1,GCSAML,PRSS33,IL1RL1,CCL26,PHEX,IL5RA,STAP1,CD24,GATA1,CD200R1,RHOBTB3,ALOX15,LGALS12,TLE1,YES1,SCN3B,S1PR1,SOCS2,NDFIP2,NTRK1 |
| GO:BP | engulfment of apoptotic cell | 2.17E-02 | | | RHOBTB3,ALOX15 |
| GO:BP | regulation of membrane invagination | 2.17E-02 | | | STAP1,ALOX15 |
| GO:BP | cardiac muscle cell contraction | 2.17E-02 | | | KCNH2,PDE4D,SCN3B |
| GO:BP | immune response-regulating cell surface receptor signaling pathway | 2.52E-02 | | | PDE4D,GCSAML,STAP1,CD24,CD200R1,YES1 |
| GO:BP | neuroinflammatory response | 2.70E-02 | | | IL4,STAP1,CD200R1 |
| GO:BP | regulation of cell population proliferation | 2.95E-02 | | | SMPD3,IL4,CCL26,IL5RA,CD24,GATA1,CLC,YES1,DACH1,S1PR1,NTRK1 |
| GO:BP | regulation of biological quality | 3.14E-02 | | | KCNH2,SMPD3,IL4,PDE4D,SYNE1,CCL26,HDC,CD24,GATA1,RHOBTB3,ALOX15,FLVCR1,YES1,SCN3B,IGF2BP2,S1PR1,SOCS2,NTRK1 |
| GO:BP | regulation of leukocyte migration | 3.14E-02 | | | SMPD3,GCSAML,STAP1,CD200R1 |
| GO:BP | regulation of protein phosphorylation | 3.14E-02 | | | SMPD3,IL4,PDE4D,CCL26,STAP1,CD24,GATA1,ALOX15,YES1,NTRK1 |
| GO:BP | localization of cell | 3.14E-02 | | | SMPD3,IL4,PDE4D,GCSAML,CCL26,STAP1,CD24,CD200R1,YES1,DACH1,S1PR1 |
| GO:BP | regulation of cell migration | 3.14E-02 | | | SMPD3,IL4,GCSAML,CCL26,STAP1,CD200R1,DACH1,S1PR1 |
| GO:BP | cell motility | 3.14E-02 | | | SMPD3,IL4,PDE4D,GCSAML,CCL26,STAP1,CD24,CD200R1,YES1,DACH1,S1PR1 |
| GO:BP | multicellular organismal signaling | 3.14E-02 | | | KCNH2,PDE4D,SCN3B,S1PR1 |
| GO:BP | cellular response to chemical stimulus | 3.14E-02 | | | KCNH2,SMPD3,IL4,PDE4D,IL1RL1,CCL26,PHEX,IL5RA,STAP1,CD24,GATA1,ALOX15,YES1,S1PR1,SOCS2,NTRK1 |
| GO:BP | regulation of actin cytoskeleton organization | 3.21E-02 | | | CCL26,STAP1,RHOBTB3,ALOX15,S1PR1 |
| GO:BP | myeloid leukocyte migration | 3.30E-02 | | | PDE4D,CCL26,STAP1,CD200R1 |
| GO:BP | positive regulation of protein modification process | 3.30E-02 | | | IL4,CCL26,STAP1,CD24,GATA1,ALOX15,YES1,NDFIP2,NTRK1 |
| GO:BP | negative regulation of neuroinflammatory response | 3.41E-02 | | | IL4,CD200R1 |
| GO:BP | inflammatory response | 3.48E-02 | | | IL4,IL1RL1,CCL26,IL5RA,STAP1,CD200R1,ALOX15 |
| GO:BP | positive regulation of protein phosphorylation | 3.55E-02 | | | IL4,CCL26,STAP1,CD24,GATA1,ALOX15,YES1,NTRK1 |
| GO:BP | leukocyte chemotaxis | 3.55E-02 | | | PDE4D,CCL26,STAP1,S1PR1 |
| GO:BP | phosphatidylethanolamine metabolic process | 3.55E-02 | | | ALOX15,PLAAT5 |
| GO:BP | regulation of phosphate metabolic process | 3.55E-02 | | | SMPD3,IL4,PDE4D,CCL26,STAP1,CD24,GATA1,ALOX15,YES1,SOCS2,NTRK1 |
| GO:BP | signal transduction | 3.55E-02 | | | SIGLEC8,SMPD3,IL4,PDE4D,VSTM1,GCSAML,PRSS33,IL1RL1,CCL26,IL5RA,STAP1,CD24,GATA1,CD200R1,RHOBTB3,ALOX15,LGALS12,TLE1,YES1,S1PR1,SOCS2,NDFIP2,NTRK1 |
| GO:BP | regulation of heart rate | 3.55E-02 | | | KCNH2,PDE4D,SCN3B |
| GO:BP | regulation of phosphorus metabolic process | 3.55E-02 | | | SMPD3,IL4,PDE4D,CCL26,STAP1,CD24,GATA1,ALOX15,YES1,SOCS2,NTRK1 |
| GO:BP | regulation of cell motility | 3.60E-02 | | | SMPD3,IL4,GCSAML,CCL26,STAP1,CD200R1,DACH1,S1PR1 |
| GO:BP | development of primary sexual characteristics | 3.64E-02 | | | GATA1,RHOBTB3,DACH1,NTRK1 |
| GO:BP | positive regulation of heart rate | 3.68E-02 | | | PDE4D,SCN3B |
| GO:BP | positive regulation of leukocyte activation | 3.74E-02 | | | IL4,IL1RL1,STAP1,CD24,YES1 |
| GO:BP | regulation of leukocyte proliferation | 3.84E-02 | | | IL4,IL5RA,CD24,CLC |
| GO:BP | regulation of leukocyte activation | 3.85E-02 | | | IL4,IL1RL1,STAP1,CD24,CLC,YES1 |
| GO:BP | ossification | 3.89E-02 | | | SMPD3,PHEX,GATA1,ALOX15,S1PR1 |
| GO:BP | positive regulation of response to stimulus | 3.89E-02 | | | IL4,PDE4D,GCSAML,IL1RL1,CCL26,STAP1,CD24,ALOX15,YES1,S1PR1,SOCS2,NDFIP2,NTRK1 |
| GO:BP | positive regulation of macrophage activation | 3.89E-02 | | | IL1RL1,STAP1 |
| GO:BP | regulation of protein modification process | 3.89E-02 | | | SMPD3,IL4,PDE4D,CCL26,STAP1,CD24,GATA1,ALOX15,YES1,NDFIP2,NTRK1 |
| GO:BP | intracellular signal transduction | 3.95E-02 | | | SMPD3,PDE4D,PRSS33,CCL26,IL5RA,CD24,CD200R1,RHOBTB3,ALOX15,LGALS12,TLE1,SOCS2,NDFIP2,NTRK1 |
| GO:BP | positive regulation of cell activation | 3.96E-02 | | | IL4,IL1RL1,STAP1,CD24,YES1 |
| GO:BP | regulation of B cell receptor signaling pathway | 4.02E-02 | | | GCSAML,STAP1 |
| GO:BP | localization | 4.08E-02 | | | KCNH2,SMPD3,IL4,PDE4D,GCSAML,IL1RL1,SYNE1,CCL26,STAP1,CD24,CD200R1,SLC16A14,RHOBTB3,ALOX15,FLVCR1,YES1,SCN3B,IGF2BP2,CCZ1B,DACH1,S1PR1,OSBPL6,SOCS2,NDFIP2 |
| GO:BP | regulation of locomotion | 4.21E-02 | | | SMPD3,IL4,GCSAML,CCL26,STAP1,CD200R1,DACH1,S1PR1 |
| GO:BP | immune response | 4.21E-02 | | | IL4,PDE4D,GCSAML,IL1RL1,CCL26,IL5RA,STAP1,CD24,CD200R1,CLC,ALOX15,YES1 |
| GO:BP | positive regulation of phosphorylation | 4.21E-02 | | | IL4,CCL26,STAP1,CD24,GATA1,ALOX15,YES1,NTRK1 |
| GO:BP | regulation of cell activation | 4.21E-02 | | | IL4,IL1RL1,STAP1,CD24,CLC,YES1 |
| GO:BP | actin-mediated cell contraction | 4.28E-02 | | | KCNH2,PDE4D,SCN3B |
| GO:BP | phagocytosis, engulfment | 4.28E-02 | | | STAP1,RHOBTB3,ALOX15 |
| GO:BP | erythrocyte development | 4.28E-02 | | | GATA1,FLVCR1 |
| GO:BP | chemotaxis | 4.31E-02 | | | PDE4D,CCL26,STAP1,RHOBTB3,S1PR1,NTRK1 |
| GO:BP | taxis | 4.33E-02 | | | PDE4D,CCL26,STAP1,RHOBTB3,S1PR1,NTRK1 |
| GO:BP | protein phosphorylation | 4.66E-02 | | | SMPD3,IL4,PDE4D,CCL26,IL5RA,STAP1,CD24,GATA1,ALOX15,YES1,NTRK1 |
| GO:BP | peptidyl-tyrosine autophosphorylation | 4.72E-02 | | | YES1,NTRK1 |
| GO:BP | sex differentiation | 4.72E-02 | | | GATA1,RHOBTB3,DACH1,NTRK1 |
| GO:BP | cell population proliferation | 4.72E-02 | | | SMPD3,IL4,CCL26,IL5RA,CD24,GATA1,CLC,YES1,DACH1,S1PR1,NTRK1 |
| GO:BP | positive regulation of cell-cell adhesion | 4.72E-02 | | | IL4,CD24,ALOX15,YES1 |
| GO:BP | membrane depolarization during action potential | 4.72E-02 | | | KCNH2,SCN3B |
| GO:BP | immune response-regulating signaling pathway | 4.75E-02 | | | PDE4D,GCSAML,STAP1,CD24,CD200R1,YES1 |
| GO:BP | plasma membrane invagination | 4.75E-02 | | | STAP1,RHOBTB3,ALOX15 |
| GO:BP | positive regulation of phosphate metabolic process | 4.82E-02 | | | IL4,CCL26,STAP1,CD24,GATA1,ALOX15,YES1,NTRK1 |
| GO:BP | response to organic substance | 4.82E-02 | | | SMPD3,IL4,PDE4D,IL1RL1,CCL26,PHEX,IL5RA,STAP1,CD24,GATA1,ALOX15,YES1,S1PR1,SOCS2,NTRK1 |
| GO:BP | positive regulation of phosphorus metabolic process | 4.82E-02 | | | IL4,CCL26,STAP1,CD24,GATA1,ALOX15,YES1,NTRK1 |
| GO:BP | regulation of heart rate by cardiac conduction | 4.88E-02 | | | KCNH2,SCN3B |
| GO:BP | ventricular cardiac muscle cell action potential | 4.88E-02 | | | KCNH2,SCN3B |
| GO:MF | cytokine receptor binding | 4.97E-02 | | | IL4,CCL26,STAP1,SOCS2,NTRK1 |
| GO:CC | side of membrane | 3.06E-02 | | | IL1RL1,IL5RA,CD24,CD200R1,ALOX15,YES1,S1PR1 |
| KEGG | Hematopoietic cell lineage | 2.79E-02 | | | IL4,IL5RA,CD24 |
| KEGG | Cytokine-cytokine receptor interaction | 3.45E-02 | | | IL4,IL1RL1,CCL26,IL5RA |
| KEGG | JAK-STAT signaling pathway | 4.35E-02 | | | IL4,IL5RA,SOCS2 |
| REACTOME | Signaling by Interleukins | 9.47E-03 | | | IL4,IL1RL1,IL5RA,ALOX15,YES1,S1PR1,SOCS2 |

***FIGURE S1*** *Differential expression analysis in* ***A.*** *whole blood and* ***B.*** *sputum cells between ex and current smokers (primary analysis; baseline samples). Volcano plot depicting all detected probe sets and coloured by fold change (FC) and adjusted p-value (pFDR): green, FC >|1.3| and pFDR <0.05; red, pFDR<0.05; orange, FC>|1.3 |.*

***
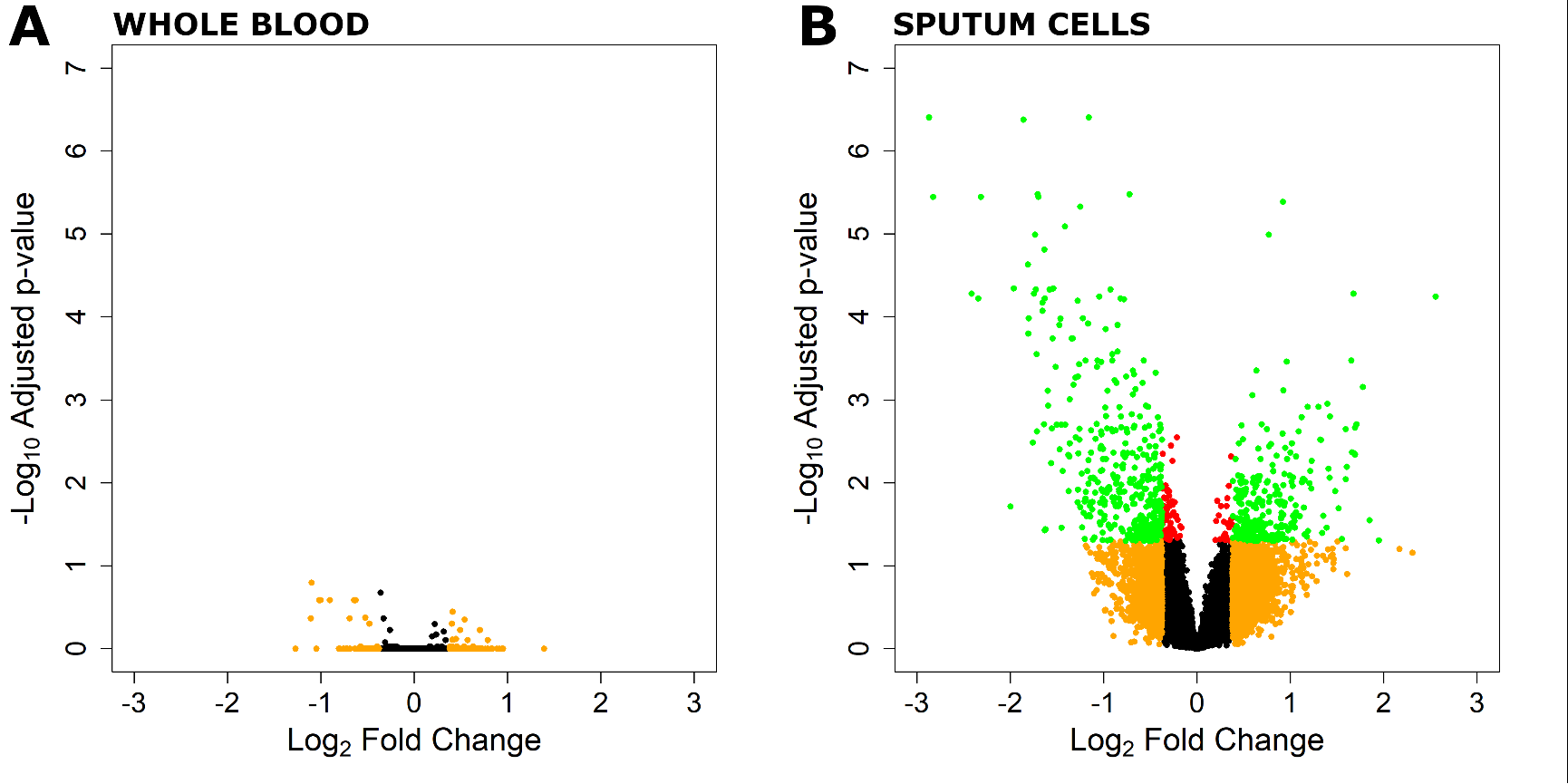
***

***TABLE S2*** *Probe sets significantly (pFDR<0.05) differentially expressed in sputum cells between ex and current smokers (primary analysis; baseline samples). pFDR, adjusted p-value*

| Probe set ID | Gene Symbol | pFDR value | Fold Change | Gene Title |
| --- | --- | --- | --- | --- |
| 239887_at | LOC101928554 | 3.92E-07 | -7.33 | uncharacterized LOC101928554 |
| 209468_at | LRP5 | 3.92E-07 | -2.24 | LDL receptor related protein 5 |
| 229055_at | GPR68 | 4.19E-07 | -3.63 | G protein-coupled receptor 68 |
| 205749_at | CYP1A1 | 3.32E-06 | -3.27 | cytochrome P450 family 1 subfamily A member 1 |
| 214582_at | PDE3B | 3.32E-06 | -1.66 | phosphodiesterase 3B |
| 219752_at | RASAL1 | 3.57E-06 | -4.98 | RAS protein activator like 1 |
| 1569909_at | KRT79 | 3.57E-06 | -3.25 | keratin 79 |
| 229354_at | AHRR | 3.57E-06 | -7.10 | aryl-hydrocarbon receptor repressor |
| 1560396_at | KLHL6 | 4.11E-06 | 1.89 | kelch like family member 6 |
| 213462_at | NPAS2 | 4.71E-06 | -2.38 | neuronal PAS domain protein 2 |
| 204964_s_at | SSPN | 8.11E-06 | -2.67 | sarcospan |
| 244652_at | CD101 | 1.02E-05 | -3.33 | CD101 molecule |
| 1560397_s_at | KLHL6 | 1.02E-05 | 1.70 | kelch like family member 6 |
| 230976_at | AK8 | 1.54E-05 | -3.12 | adenylate kinase 8 |
| 202436_s_at | CYP1B1 | 2.33E-05 | -3.51 | cytochrome P450 family 1 subfamily B member 1 |
| 207167_at | CD101 | 4.52E-05 | -2.91 | CD101 molecule |
| 202435_s_at | CYP1B1 | 4.52E-05 | -3.91 | cytochrome P450 family 1 subfamily B member 1 |
| 201468_s_at | NQO1 | 4.67E-05 | -3.31 | NAD(P)H quinone dehydrogenase 1 |
| 227336_at | DTX1 | 4.67E-05 | -1.90 | deltex E3 ubiquitin ligase 1 |
| 243783_at | --- | 4.67E-05 | -3.00 | --- |
| 226632_at | CYGB | 5.25E-05 | -3.36 | cytoglobin |
| 206569_at | IL24 | 5.25E-05 | -5.34 | interleukin 24 |
| 203868_s_at | VCAM1 | 5.25E-05 | 3.19 | vascular cell adhesion molecule 1 |
| 48106_at | SLC48A1 | 5.69E-05 | -2.07 | solute carrier family 48 member 1 |
| 201505_at | LAMB1 | 5.69E-05 | 5.87 | laminin subunit beta 1 |
| 228339_at | ECSCR | 6.02E-05 | -5.08 | endothelial cell surface expressed chemotaxis and apoptosis regulator |
| 241358_at | WFIKKN2 | 6.02E-05 | -3.11 | WAP, follistatin/kazal, immunoglobulin, kunitz and netrin domain containing 2 |
| 233088_at | --- | 6.02E-05 | -1.77 | --- |
| 1553572_a_at | CYGB | 6.15E-05 | -1.73 | cytoglobin |
| 229168_at | COL23A1 | 6.38E-05 | -2.43 | collagen type XXIII alpha 1 chain |
| 227780_s_at | ECSCR | 6.72E-05 | -3.16 | endothelial cell surface expressed chemotaxis and apoptosis regulator |
| 220301_at | CCDC102B | 8.46E-05 | -3.16 | coiled-coil domain containing 102B |
| 210519_s_at | NQO1 | 1.04E-04 | -3.50 | NAD(P)H quinone dehydrogenase 1 |
| 218417_s_at | SLC48A1 | 1.04E-04 | -2.34 | solute carrier family 48 member 1 |
| 227779_at | ECSCR | 1.05E-04 | -2.76 | endothelial cell surface expressed chemotaxis and apoptosis regulator |
| 234986_at | GCLM | 1.20E-04 | -2.25 | glutamate-cysteine ligase modifier subunit |
| 202437_s_at | CYP1B1 | 1.25E-04 | -2.78 | cytochrome P450 family 1 subfamily B member 1 |
| 222317_at | PDE3B | 1.26E-04 | -1.81 | phosphodiesterase 3B |
| 223635_s_at | SSBP3 | 1.40E-04 | -1.97 | single stranded DNA binding protein 3 |
| 219837_s_at | CYTL1 | 1.59E-04 | -3.50 | cytokine like 1 |
| 220745_at | IL19 | 1.82E-04 | -2.52 | interleukin 19 |
| 205623_at | ALDH3A1 | 1.82E-04 | -2.93 | aldehyde dehydrogenase 3 family member A1 |
| 207315_at | CD226 | 1.82E-04 | -2.54 | CD226 molecule |
| 206126_at | CXCR5 | 2.60E-04 | -1.81 | C-X-C motif chemokine receptor 5 |
| 201467_s_at | NQO1 | 2.82E-04 | -3.30 | NAD(P)H quinone dehydrogenase 1 |
| 217991_x_at | SSBP3 | 2.82E-04 | -1.88 | single stranded DNA binding protein 3 |
| 222033_s_at | FLT1 | 3.34E-04 | -2.10 | fms related tyrosine kinase 1 |
| 1556312_at | PCARE | 3.34E-04 | -1.49 | photoreceptor cilium actin regulator |
| 239501_at | --- | 3.34E-04 | -1.88 | --- |
| 241418_at | NMRAL2P | 3.34E-04 | -2.29 | NmrA like redox sensor 2, pseudogene |
| 235229_at | --- | 3.34E-04 | 3.14 | --- |
| 231776_at | EOMES | 3.45E-04 | 1.94 | eomesodermin |
| 213725_x_at | XYLT1 | 3.48E-04 | -2.04 | xylosyltransferase 1 |
| 236140_at | GCLM | 3.70E-04 | -2.41 | glutamate-cysteine ligase modifier subunit |
| 229137_at | FUCA1 | 3.98E-04 | -2.86 | alpha-L-fucosidase 1 |
| 219509_at | MYOZ1 | 3.98E-04 | -2.10 | myozenin 1 |
| 1555275_a_at | KLHL6 | 4.41E-04 | 1.55 | kelch like family member 6 |
| 209994_s_at | ABCB1 /// ABCB4 | 4.43E-04 | -1.61 | ATP binding cassette subfamily B member 1 /// ATP binding cassette subfamily B member 4 |
| 214128_at | DAGLA | 4.69E-04 | -1.36 | diacylglycerol lipase alpha |
| 220778_x_at | SEMA6B | 4.90E-04 | -1.60 | semaphorin 6B |
| 39549_at | NPAS2 | 5.21E-04 | -2.42 | neuronal PAS domain protein 2 |
| 204759_at | RCBTB2 | 5.21E-04 | -1.69 | RCC1 and BTB domain containing protein 2 |
| 239001_at | MGST1 | 5.38E-04 | -2.47 | microsomal glutathione S-transferase 1 |
| 204119_s_at | ADK | 5.78E-04 | -1.85 | adenosine kinase |
| 208373_s_at | P2RY6 | 6.22E-04 | -1.82 | pyrimidinergic receptor P2Y6 |
| 201118_at | PGD | 6.22E-04 | -1.50 | phosphogluconate dehydrogenase |
| 209541_at | IGF1 | 6.56E-04 | -2.51 | insulin like growth factor 1 |
| 238439_at | ANKRD22 | 7.00E-04 | 3.42 | ankyrin repeat domain 22 |
| 222482_at | SSBP3 | 7.47E-04 | -1.58 | single stranded DNA binding protein 3 |
| 206109_at | FUT1 | 7.66E-04 | 1.90 | fucosyltransferase 1 (H blood group) |
| 205743_at | STAC | 7.74E-04 | -3.03 | SH3 and cysteine rich domain |
| 203196_at | ABCC4 | 7.75E-04 | -1.95 | ATP binding cassette subfamily C member 4 |
| 202275_at | G6PD | 8.58E-04 | -1.61 | glucose-6-phosphate dehydrogenase |
| 1559582_at | RHOQ | 8.79E-04 | 1.50 | ras homolog family member Q |
| 221019_s_at | COLEC12 | 9.78E-04 | -2.58 | collectin subfamily member 12 |
| 220423_at | PLA2G2D | 1.11E-03 | 2.63 | phospholipase A2 group IID |
| 1556314_a_at | --- | 1.17E-03 | -3.02 | --- |
| 202588_at | AK1 | 1.17E-03 | -1.46 | adenylate kinase 1 |
| 223597_at | ITLN1 | 1.21E-03 | 2.46 | intelectin 1 |
| 225609_at | GSR | 1.21E-03 | -1.44 | glutathione-disulfide reductase |
| 211651_s_at | LAMB1 | 1.21E-03 | 2.27 | laminin subunit beta 1 |
| 227857_at | APMAP | 1.21E-03 | -1.45 | adipocyte plasma membrane associated protein |
| 225252_at | SRXN1 | 1.23E-03 | -1.78 | sulfiredoxin 1 |
| 206595_at | CST6 | 1.24E-03 | -1.98 | cystatin E/M |
| 208591_s_at | PDE3B | 1.49E-03 | -1.62 | phosphodiesterase 3B |
| 219475_at | OSGIN1 | 1.57E-03 | -1.97 | oxidative stress induced growth inhibitor 1 |
| 211165_x_at | EPHB2 | 1.58E-03 | -1.53 | EPH receptor B2 |
| 213825_at | OLIG2 | 1.58E-03 | 2.68 | oligodendrocyte transcription factor 2 |
| 223567_at | SEMA6B | 1.58E-03 | -1.76 | semaphorin 6B |
| 234994_at | TMEM200A | 1.62E-03 | 2.17 | transmembrane protein 200A |
| 233144_s_at | RASAL1 | 1.62E-03 | -1.34 | RAS protein activator like 1 |
| 224950_at | PTGFRN | 1.94E-03 | -2.11 | prostaglandin F2 receptor inhibitor |
| 205890_s_at | UBD /// GABBR1 | 1.98E-03 | 3.26 | ubiquitin D /// gamma-aminobutyric acid type B receptor subunit 1 |
| 241695_s_at | --- | 1.98E-03 | 1.61 | --- |
| 1556046_a_at | LINC00599 /// MIR124-1 | 1.98E-03 | -3.12 | long intergenic non-protein coding RNA 599 /// microRNA 124-1 |
| 214769_at | CLCN4 | 2.00E-03 | -2.84 | chloride voltage-gated channel 4 |
| 206656_s_at | APMAP | 2.00E-03 | -1.32 | adipocyte plasma membrane associated protein |
| 226534_at | KITLG | 2.00E-03 | -2.75 | KIT ligand |
| 207469_s_at | PIR | 2.00E-03 | -2.67 | pirin |
| 1569030_s_at | NUB1 | 2.03E-03 | 1.39 | negative regulator of ubiquitin like proteins 1 |
| 224480_s_at | GPAT3 | 2.05E-03 | -1.60 | glycerol-3-phosphate acyltransferase 3 |
| 209453_at | SLC9A1 | 2.05E-03 | -1.43 | solute carrier family 9 member A1 |
| 230910_s_at | LY6K /// LNCOC1 | 2.14E-03 | -1.76 | lymphocyte antigen 6 family member K /// lncRNA associated with ovarian cancer 1 |
| 228170_at | OLIG1 | 2.16E-03 | 3.23 | oligodendrocyte transcription factor 1 |
| 218416_s_at | SLC48A1 | 2.16E-03 | -1.61 | solute carrier family 48 member 1 |
| 205364_at | ACOX2 | 2.16E-03 | -1.48 | acyl-CoA oxidase 2 |
| 204720_s_at | DNAJC6 | 2.21E-03 | -2.94 | DnaJ heat shock protein family (Hsp40) member C6 |
| 209953_s_at | CDC37 | 2.21E-03 | -1.31 | cell division cycle 37 |
| 231120_x_at | PKIB | 2.21E-03 | -1.95 | cAMP-dependent protein kinase inhibitor beta |
| 205891_at | ADORA2B | 2.23E-03 | -2.40 | adenosine A2b receptor |
| 39548_at | NPAS2 | 2.25E-03 | -1.69 | neuronal PAS domain protein 2 |
| 206742_at | VEGFD /// PIR-FIGF | 2.25E-03 | -1.89 | vascular endothelial growth factor D /// PIR-FIGF readthrough |
| 242176_at | MEF2A | 2.25E-03 | 1.68 | myocyte enhancer factor 2A |
| 228890_at | ATOH8 | 2.25E-03 | 3.01 | atonal bHLH transcription factor 8 |
| 231736_x_at | MGST1 | 2.32E-03 | -2.20 | microsomal glutathione S-transferase 1 |
| 205741_s_at | DTNA | 2.32E-03 | -1.83 | dystrobrevin alpha |
| 206749_at | CD1B | 2.39E-03 | -3.29 | CD1b molecule |
| 224918_x_at | MGST1 | 2.39E-03 | -2.04 | microsomal glutathione S-transferase 1 |
| 233701_at | --- | 2.40E-03 | -1.33 | --- |
| 213337_s_at | SOCS1 | 2.40E-03 | 2.12 | suppressor of cytokine signaling 1 |
| 202831_at | GPX2 | 2.41E-03 | -1.87 | glutathione peroxidase 2 |
| 221601_s_at | FCMR | 2.48E-03 | -1.68 | Fc fragment of IgM receptor |
| 208389_s_at | SLC1A2 | 2.54E-03 | 1.89 | solute carrier family 1 member 2 |
| 204120_s_at | ADK | 2.61E-03 | -2.04 | adenosine kinase |
| 224182_x_at | SEMA6B | 2.71E-03 | -1.39 | semaphorin 6B |
| 220570_at | RETN | 2.82E-03 | -2.47 | resistin |
| 201463_s_at | TALDO1 | 2.83E-03 | -1.16 | transaldolase 1 |
| 34726_at | CACNB3 | 2.95E-03 | -2.13 | calcium voltage-gated channel auxiliary subunit beta 3 |
| 225626_at | PAG1 | 2.98E-03 | 1.41 | phosphoprotein membrane anchor with glycosphingolipid microdomains 1 |
| 213252_at | SH3PXD2A | 3.01E-03 | -1.30 | SH3 and PX domains 2A |
| 1555416_a_at | ALOX15B | 3.01E-03 | 2.49 | arachidonate 15-lipoxygenase type B |
| 223687_s_at | LY6K | 3.01E-03 | -2.40 | lymphocyte antigen 6 family member K |
| 210001_s_at | SOCS1 | 3.04E-03 | 2.51 | suppressor of cytokine signaling 1 |
| 225766_s_at | TNPO1 | 3.05E-03 | -1.48 | transportin 1 |
| 210325_at | CD1A | 3.26E-03 | -3.40 | CD1a molecule |
| 239005_at | --- | 3.34E-03 | 2.02 | --- |
| 214298_x_at | SEPTIN6 | 3.34E-03 | 1.36 | septin 6 |
| 209841_s_at | LRRN3 | 3.34E-03 | -1.71 | leucine rich repeat neuronal 3 |
| 223551_at | PKIB | 3.35E-03 | -2.58 | cAMP-dependent protein kinase inhibitor beta |
| 206633_at | CHRNA1 | 3.39E-03 | 1.73 | cholinergic receptor nicotinic alpha 1 subunit |
| 225118_at | KMT5A | 3.57E-03 | -1.22 | lysine methyltransferase 5A |
| 219904_at | ZSCAN5A | 3.58E-03 | -2.04 | zinc finger and SCAN domain containing 5A |
| 52975_at | MVB12B | 3.64E-03 | 1.71 | multivesicular body subunit 12B |
| 205131_x_at | CLEC11A | 3.64E-03 | -1.43 | C-type lectin domain containing 11A |
| 232353_s_at | STYXL1 | 3.64E-03 | -1.36 | serine/threonine/tyrosine interacting like 1 |
| 212558_at | SPRY1 | 3.77E-03 | 1.92 | sprouty RTK signaling antagonist 1 |
| 226454_at | MARCH9 | 3.86E-03 | 1.57 | membrane associated ring-CH-type finger 9 |
| 209921_at | SLC7A11 | 3.86E-03 | -2.05 | solute carrier family 7 member 11 |
| 203925_at | GCLM | 3.92E-03 | -2.01 | glutamate-cysteine ligase modifier subunit |
| 220741_s_at | PPA2 | 3.92E-03 | -1.53 | pyrophosphatase (inorganic) 2 |
| 214839_at | LINC00599 /// MIR124-1 | 3.93E-03 | -2.78 | long intergenic non-protein coding RNA 599 /// microRNA 124-1 |
| 206714_at | ALOX15B | 4.30E-03 | 3.15 | arachidonate 15-lipoxygenase type B |
| 1553141_at | LACC1 | 4.31E-03 | -1.73 | laccase domain containing 1 |
| 1562253_at | SLC7A11-AS1 | 4.32E-03 | -2.20 | SLC7A11 antisense RNA 1 |
| 220935_s_at | CDK5RAP2 | 4.32E-03 | -1.87 | CDK5 regulatory subunit associated protein 2 |
| 1562957_at | --- | 4.33E-03 | 2.00 | --- |
| 228937_at | LACC1 | 4.37E-03 | -1.62 | laccase domain containing 1 |
| 210354_at | IFNG | 4.37E-03 | 3.20 | interferon gamma |
| 207819_s_at | ABCB4 | 4.37E-03 | -1.68 | ATP binding cassette subfamily B member 4 |
| 1565162_s_at | MGST1 | 4.40E-03 | -2.42 | microsomal glutathione S-transferase 1 |
| 218321_x_at | STYXL1 | 4.40E-03 | -1.37 | serine/threonine/tyrosine interacting like 1 |
| 228309_at | APMAP | 4.47E-03 | -1.29 | adipocyte plasma membrane associated protein |
| 235010_at | ZBED5-AS1 | 4.54E-03 | -1.49 | ZBED5 antisense RNA 1 |
| 239122_at | --- | 4.54E-03 | -1.74 | --- |
| 208607_s_at | SAA2 /// SAA1 /// SAA2-SAA4 | 4.54E-03 | 3.23 | serum amyloid A2 /// serum amyloid A1 /// SAA2-SAA4 readthrough |
| 213706_at | GPD1 | 4.59E-03 | -2.61 | glycerol-3-phosphate dehydrogenase 1 |
| 230370_x_at | STYXL1 | 4.61E-03 | -1.38 | serine/threonine/tyrosine interacting like 1 |
| 204358_s_at | FLRT2 | 4.63E-03 | -1.46 | fibronectin leucine rich transmembrane protein 2 |
| 242974_at | CD47 | 4.72E-03 | 1.80 | CD47 molecule |
| 205596_s_at | SMURF2 | 4.78E-03 | 1.29 | SMAD specific E3 ubiquitin protein ligase 2 |
| 220491_at | HAMP | 4.78E-03 | 2.08 | hepcidin antimicrobial peptide |
| 228057_at | DDIT4L | 4.78E-03 | -2.59 | DNA damage inducible transcript 4 like |
| 238032_at | --- | 4.90E-03 | -1.71 | --- |
| 200681_at | GLO1 | 4.92E-03 | -1.57 | glyoxalase I |
| 220162_s_at | CARD9 /// DNLZ | 4.97E-03 | -1.37 | caspase recruitment domain family member 9 /// DNL-type zinc finger |
| 232615_at | --- | 5.15E-03 | 1.60 | --- |
| 205853_at | ZBTB7B | 5.15E-03 | 1.33 | zinc finger and BTB domain containing 7B |
| 226226_at | TMEM45B | 5.15E-03 | -1.97 | transmembrane protein 45B |
| 213482_at | DOCK3 | 5.15E-03 | -2.02 | dedicator of cytokinesis 3 |
| 221756_at | PIK3IP1 | 5.15E-03 | 1.95 | phosphoinositide-3-kinase interacting protein 1 |
| 1560553_at | TIAF1 | 5.25E-03 | -1.43 | TGFB1-induced anti-apoptotic factor 1 |
| 217678_at | SLC7A11 | 5.30E-03 | -2.24 | solute carrier family 7 member 11 |
| 203722_at | ALDH4A1 | 5.43E-03 | -1.20 | aldehyde dehydrogenase 4 family member A1 |
| 214456_x_at | SAA2 /// SAA1 | 5.43E-03 | 2.34 | serum amyloid A2 /// serum amyloid A1 |
| 1553151_at | ATP6V0D2 | 5.76E-03 | -2.96 | ATPase H+ transporting V0 subunit d2 |
| 212891_s_at | GADD45GIP1 | 6.04E-03 | -1.32 | GADD45G interacting protein 1 |
| 229144_at | KAZN | 6.05E-03 | -1.73 | kazrin, periplakin interacting protein |
| 227606_s_at | STAMBPL1 | 6.10E-03 | 1.75 | STAM binding protein like 1 |
| 219458_s_at | NSUN3 | 6.17E-03 | -1.31 | NOP2/Sun RNA methyltransferase 3 |
| 220346_at | MTHFD2L | 6.17E-03 | -1.83 | methylenetetrahydrofolate dehydrogenase (NADP+ dependent) 2 like |
| 242714_at | LOC101928429 | 6.22E-03 | -1.74 | uncharacterized LOC101928429 |
| 233982_x_at | STYXL1 | 6.22E-03 | -1.36 | serine/threonine/tyrosine interacting like 1 |
| 239196_at | ANKRD22 | 6.40E-03 | 3.03 | ankyrin repeat domain 22 |
| 213093_at | PRKCA | 6.53E-03 | -1.59 | protein kinase C alpha |
| 1562996_at | --- | 6.71E-03 | -1.74 | --- |
| 200992_at | IPO7 | 6.75E-03 | -1.38 | importin 7 |
| 225491_at | SLC1A2 | 6.76E-03 | 2.65 | solute carrier family 1 member 2 |
| 210651_s_at | EPHB2 | 6.92E-03 | -1.31 | EPH receptor B2 |
| 230266_at | RAB7B | 7.03E-03 | -2.03 | RAB7B, member RAS oncogene family |
| 209588_at | EPHB2 | 7.18E-03 | -2.26 | EPH receptor B2 |
| 204614_at | SERPINB2 | 7.18E-03 | -2.72 | serpin family B member 2 |
| 227629_at | PRLR | 7.18E-03 | 2.32 | prolactin receptor |
| 240397_x_at | --- | 7.18E-03 | -2.05 | --- |
| 212279_at | TMEM97 | 7.18E-03 | -1.44 | transmembrane protein 97 |
| 213856_at | CD47 | 7.20E-03 | 1.76 | CD47 molecule |
| 227491_at | ELOVL6 | 7.24E-03 | 1.76 | ELOVL fatty acid elongase 6 |
| 221463_at | CCL24 | 7.77E-03 | -2.36 | C-C motif chemokine ligand 24 |
| 202898_at | SDC3 | 7.78E-03 | 1.91 | syndecan 3 |
| 227144_at | KIAA0930 | 8.00E-03 | -1.87 | KIAA0930 |
| 231559_at | NNMT | 8.15E-03 | 1.98 | nicotinamide N-methyltransferase |
| 212057_at | GSE1 | 8.17E-03 | 1.35 | Gse1 coiled-coil protein |
| 1562955_at | --- | 8.28E-03 | 1.59 | --- |
| 207911_s_at | TGM5 | 8.28E-03 | -1.46 | transglutaminase 5 |
| 223321_s_at | FGFRL1 | 8.28E-03 | -1.43 | fibroblast growth factor receptor like 1 |
| 1562733_at | LINC00092 | 8.28E-03 | -1.50 | long intergenic non-protein coding RNA 92 |
| 229344_x_at | RIMKLB | 8.39E-03 | 1.39 | ribosomal modification protein rimK like family member B |
| 1553715_s_at | MCRIP2 | 8.39E-03 | -1.31 | MAPK regulated corepressor interacting protein 2 |
| 227384_s_at | LINC00869 | 8.41E-03 | 1.56 | long intergenic non-protein coding RNA 869 |
| 213125_at | OLFML2B | 8.54E-03 | 2.03 | olfactomedin like 2B |
| 233540_s_at | CDK5RAP2 | 8.57E-03 | -1.81 | CDK5 regulatory subunit associated protein 2 |
| 205148_s_at | CLCN4 | 8.69E-03 | -2.16 | chloride voltage-gated channel 4 |
| 228935_at | SLC4A8 | 8.69E-03 | -1.83 | solute carrier family 4 member 8 |
| 208747_s_at | C1S | 8.69E-03 | 2.67 | complement C1s |
| 230536_at | PBX4 | 8.69E-03 | 1.52 | PBX homeobox 4 |
| 212315_s_at | NUP210 | 8.71E-03 | -1.33 | nucleoporin 210 |
| 231472_at | FBXO15 | 8.71E-03 | -2.21 | F-box protein 15 |
| 57082_at | LDLRAP1 | 8.77E-03 | -1.85 | low density lipoprotein receptor adaptor protein 1 |
| 205676_at | CYP27B1 | 8.92E-03 | 2.20 | cytochrome P450 family 27 subfamily B member 1 |
| 1561365_at | NRP1 | 8.94E-03 | -1.43 | neuropilin 1 |
| 225765_at | TNPO1 | 8.95E-03 | -1.42 | transportin 1 |
| 212611_at | DTX4 | 8.95E-03 | -2.14 | deltex E3 ubiquitin ligase 4 |
| 230015_at | PRCD | 8.97E-03 | -1.38 | photoreceptor disc component |
| 1556285_s_at | PPA2 | 9.00E-03 | -1.64 | pyrophosphatase (inorganic) 2 |
| 203915_at | CXCL9 | 9.02E-03 | 3.01 | C-X-C motif chemokine ligand 9 |
| 209530_at | CACNB3 | 9.07E-03 | -1.60 | calcium voltage-gated channel auxiliary subunit beta 3 |
| 221602_s_at | FCMR | 9.18E-03 | -1.44 | Fc fragment of IgM receptor |
| 220014_at | PRR16 | 9.24E-03 | 2.22 | proline rich 16 |
| 213666_at | SEPTIN6 | 9.45E-03 | 1.58 | septin 6 |
| 240094_at | --- | 9.53E-03 | 1.68 | --- |
| 222750_s_at | SRD5A3 | 9.53E-03 | -1.82 | steroid 5 alpha-reductase 3 |
| 205020_s_at | ARL4A | 9.53E-03 | 1.40 | ADP ribosylation factor like GTPase 4A |
| 1553882_at | --- | 9.54E-03 | -1.51 | --- |
| 240593_x_at | --- | 9.54E-03 | 1.30 | --- |
| 203528_at | SEMA4D | 9.57E-03 | 1.38 | semaphorin 4D |
| 1560109_s_at | NUB1 | 9.76E-03 | 2.02 | negative regulator of ubiquitin like proteins 1 |
| 242437_at | --- | 9.79E-03 | 1.43 | --- |
| 227262_at | HAPLN3 | 9.79E-03 | 2.18 | hyaluronan and proteoglycan link protein 3 |
| 217556_at | CLCN4 | 9.84E-03 | -2.09 | chloride voltage-gated channel 4 |
| 204044_at | QPRT | 9.86E-03 | -1.32 | quinolinate phosphoribosyltransferase |
| 230001_at | MARCH9 | 9.86E-03 | 1.68 | membrane associated ring-CH-type finger 9 |
| 46665_at | SEMA4C | 9.86E-03 | -1.65 | semaphorin 4C |
| 225622_at | PAG1 | 9.87E-03 | 1.51 | phosphoprotein membrane anchor with glycosphingolipid microdomains 1 |
| 217838_s_at | EVL | 1.01E-02 | -1.63 | Enah/Vasp-like |
| 205016_at | TGFA | 1.02E-02 | -1.54 | transforming growth factor alpha |
| 229264_at | --- | 1.02E-02 | 1.38 | --- |
| 241154_x_at | --- | 1.02E-02 | 2.30 | --- |
| 221790_s_at | LDLRAP1 | 1.02E-02 | -1.83 | low density lipoprotein receptor adaptor protein 1 |
| 214763_at | ACOT11 | 1.02E-02 | -1.53 | acyl-CoA thioesterase 11 |
| 223281_s_at | COX15 | 1.03E-02 | -1.32 | cytochrome c oxidase assembly homolog COX15 |
| 1559361_at | MACC1 /// LOC101927668 | 1.03E-02 | -2.24 | MET transcriptional regulator MACC1 /// uncharacterized LOC101927668 |
| 212421_at | KIAA0930 | 1.05E-02 | -1.76 | KIAA0930 |
| 213915_at | NKG7 | 1.06E-02 | 1.89 | natural killer cell granule protein 7 |
| 238480_at | TTC39C | 1.06E-02 | -1.57 | tetratricopeptide repeat domain 39C |
| 239258_at | --- | 1.07E-02 | 1.55 | --- |
| 1555902_at | ARMCX5 | 1.08E-02 | -1.58 | armadillo repeat containing X-linked 5 |
| 208985_s_at | EIF3J | 1.08E-02 | -1.26 | eukaryotic translation initiation factor 3 subunit J |
| 1569054_at | SLC1A3 | 1.09E-02 | 2.03 | solute carrier family 1 member 3 |
| 230820_at | SMURF2 | 1.09E-02 | 1.26 | SMAD specific E3 ubiquitin protein ligase 2 |
| 227607_at | STAMBPL1 | 1.09E-02 | 1.79 | STAM binding protein like 1 |
| 210557_x_at | CSF1 | 1.09E-02 | -1.29 | colony stimulating factor 1 |
| 1555039_a_at | ABCC4 | 1.10E-02 | -1.53 | ATP binding cassette subfamily C member 4 |
| 219039_at | SEMA4C | 1.11E-02 | -1.55 | semaphorin 4C |
| 210887_s_at | EVC | 1.12E-02 | -1.30 | EvC ciliary complex subunit 1 |
| 219432_at | EVC | 1.12E-02 | -1.93 | EvC ciliary complex subunit 1 |
| 227354_at | PAG1 | 1.13E-02 | 1.54 | phosphoprotein membrane anchor with glycosphingolipid microdomains 1 |
| 207425_s_at | SEPTIN9 | 1.17E-02 | -1.29 | septin 9 |
| 212282_at | TMEM97 | 1.17E-02 | -1.95 | transmembrane protein 97 |
| 1554668_a_at | FAM151A | 1.17E-02 | -1.27 | family with sequence similarity 151 member A |
| 218665_at | FZD4 | 1.17E-02 | 2.34 | frizzled class receptor 4 |
| 239294_at | PIK3CG | 1.17E-02 | -1.33 | phosphatidylinositol-4,5-bisphosphate 3-kinase catalytic subunit gamma |
| 218279_s_at | HIST2H2AA3 | 1.18E-02 | 1.43 | histone cluster 2 H2A family member a3 |
| 221753_at | SSH1 | 1.18E-02 | -1.46 | slingshot protein phosphatase 1 |
| 223503_at | TMEM163 | 1.18E-02 | -1.94 | transmembrane protein 163 |
| 214895_s_at | ADAM10 | 1.18E-02 | -1.36 | ADAM metallopeptidase domain 10 |
| 1562801_at | LINC02201 | 1.18E-02 | -1.48 | long intergenic non-protein coding RNA 2201 |
| 204997_at | GPD1 | 1.19E-02 | -2.04 | glycerol-3-phosphate dehydrogenase 1 |
| 243605_at | --- | 1.20E-02 | 1.81 | --- |
| 239529_at | DCANP1 /// TIFAB | 1.20E-02 | -1.79 | dendritic cell associated nuclear protein /// TIFA inhibitor |
| 215354_s_at | PELP1 | 1.20E-02 | -1.25 | proline, glutamate and leucine rich protein 1 |
| 45749_at | RIPOR1 | 1.20E-02 | 1.33 | RHO family interacting cell polarization regulator 1 |
| 1553232_at | RMDN2 | 1.20E-02 | -1.95 | regulator of microtubule dynamics 2 |
| 209686_at | S100B | 1.21E-02 | -2.42 | S100 calcium binding protein B |
| 227084_at | DTNA | 1.21E-02 | -1.92 | dystrobrevin alpha |
| 241540_at | --- | 1.21E-02 | 1.41 | --- |
| 237171_at | --- | 1.23E-02 | -1.36 | --- |
| 228167_at | KLHL6 | 1.24E-02 | 1.37 | kelch like family member 6 |
| 242753_x_at | AP1AR | 1.26E-02 | 1.39 | adaptor related protein complex 1 associated regulatory protein |
| 202237_at | NNMT | 1.26E-02 | 2.79 | nicotinamide N-methyltransferase |
| 218800_at | SRD5A3 | 1.26E-02 | -2.02 | steroid 5 alpha-reductase 3 |
| 221266_s_at | DCSTAMP | 1.26E-02 | -2.60 | dendrocyte expressed seven transmembrane protein |
| 225755_at | KLHDC8B | 1.26E-02 | 1.65 | kelch domain containing 8B |
| 209589_s_at | EPHB2 | 1.27E-02 | -1.72 | EPH receptor B2 |
| 224991_at | CMIP | 1.27E-02 | -1.23 | c-Maf inducing protein |
| 227116_at | MON1B | 1.28E-02 | -1.25 | MON1 homolog B, secretory trafficking associated |
| 219093_at | PID1 | 1.28E-02 | -1.76 | phosphotyrosine interaction domain containing 1 |
| 227559_at | NDUFAF4 | 1.28E-02 | -1.79 | NADH:ubiquinone oxidoreductase complex assembly factor 4 |
| 242677_at | --- | 1.28E-02 | -2.00 | --- |
| 207528_s_at | SLC7A11 | 1.29E-02 | -2.29 | solute carrier family 7 member 11 |
| 218280_x_at | HIST2H2AA3 /// HIST2H2AA4 | 1.29E-02 | 1.43 | histone cluster 2 H2A family member a3 /// histone cluster 2 H2A family member a4 |
| 202838_at | FUCA1 | 1.31E-02 | -2.13 | alpha-L-fucosidase 1 |
| 224937_at | PTGFRN | 1.32E-02 | -1.96 | prostaglandin F2 receptor inhibitor |
| 213624_at | SMPDL3A | 1.33E-02 | 1.52 | sphingomyelin phosphodiesterase acid like 3A |
| 230780_at | LINC00886 | 1.38E-02 | -1.37 | long intergenic non-protein coding RNA 886 |
| 233813_at | PPP1R16B | 1.38E-02 | 1.78 | protein phosphatase 1 regulatory subunit 16B |
| 217118_s_at | KIAA0930 | 1.38E-02 | -1.57 | KIAA0930 |
| 1570552_at | --- | 1.38E-02 | -1.51 | --- |
| 1556033_at | LINC01138 | 1.38E-02 | 1.84 | long intergenic non-protein coding RNA 1138 |
| 210998_s_at | HGF | 1.38E-02 | 1.36 | hepatocyte growth factor |
| 218833_at | MAP3K20 | 1.38E-02 | -1.41 | mitogen-activated protein kinase kinase kinase 20 |
| 240279_at | --- | 1.38E-02 | 1.42 | --- |
| 242414_at | QPRT | 1.39E-02 | -1.40 | quinolinate phosphoribosyltransferase |
| 224817_at | SH3PXD2A | 1.42E-02 | -1.53 | SH3 and PX domains 2A |
| 213919_at | DNAJC4 | 1.44E-02 | -1.23 | DnaJ heat shock protein family (Hsp40) member C4 |
| 240824_at | --- | 1.47E-02 | 1.71 | --- |
| 242079_at | RGS12 | 1.49E-02 | -1.62 | regulator of G protein signaling 12 |
| 205293_x_at | BAIAP2 | 1.50E-02 | -1.28 | BAI1 associated protein 2 |
| 1553982_a_at | RAB7B | 1.50E-02 | -1.84 | RAB7B, member RAS oncogene family |
| 1552626_a_at | TMEM163 | 1.50E-02 | -1.88 | transmembrane protein 163 |
| 214290_s_at | HIST2H2AA3 /// HIST2H2AA4 | 1.51E-02 | 1.39 | histone cluster 2 H2A family member a3 /// histone cluster 2 H2A family member a4 |
| 203305_at | F13A1 | 1.53E-02 | -1.79 | coagulation factor XIII A chain |
| 209714_s_at | CDKN3 | 1.53E-02 | -1.62 | cyclin dependent kinase inhibitor 3 |
| 236578_at | --- | 1.53E-02 | -2.02 | --- |
| 225848_at | ZNF746 | 1.53E-02 | -1.52 | zinc finger protein 746 |
| 214449_s_at | RHOQ | 1.53E-02 | 1.25 | ras homolog family member Q |
| 209703_x_at | METTL7A | 1.53E-02 | 1.33 | methyltransferase like 7A |
| 238246_at | TESMIN | 1.54E-02 | 1.58 | testis expressed metallothionein like protein |
| 213397_x_at | ANG /// RNASE4 | 1.54E-02 | 2.00 | angiogenin /// ribonuclease A family member 4 |
| 220428_at | CD207 | 1.54E-02 | -2.30 | CD207 molecule |
| 226373_at | SFXN5 | 1.55E-02 | -1.27 | sideroflexin 5 |
| 209542_x_at | IGF1 | 1.60E-02 | -1.42 | insulin like growth factor 1 |
| 221875_x_at | HLA-F | 1.64E-02 | 1.16 | major histocompatibility complex, class I, F |
| 41220_at | SEPTIN9 | 1.64E-02 | -1.32 | septin 9 |
| 208657_s_at | SEPTIN9 | 1.64E-02 | -1.37 | septin 9 |
| 224730_at | DCAF7 | 1.66E-02 | -1.44 | DDB1 and CUL4 associated factor 7 |
| 220684_at | TBX21 | 1.66E-02 | 1.86 | T-box 21 |
| 209770_at | BTN3A1 | 1.66E-02 | 1.62 | butyrophilin subfamily 3 member A1 |
| 211217_s_at | KCNQ1 | 1.66E-02 | -1.20 | potassium voltage-gated channel subfamily Q member 1 |
| 226042_at | EDC3 | 1.68E-02 | -1.39 | enhancer of mRNA decapping 3 |
| 1566764_at | MACC1 | 1.68E-02 | -2.31 | MET transcriptional regulator MACC1 |
| 215925_s_at | CD72 | 1.68E-02 | 1.81 | CD72 molecule |
| 240770_at | TMEM171 | 1.68E-02 | 1.56 | transmembrane protein 171 |
| 222773_s_at | GALNT12 | 1.68E-02 | -1.99 | polypeptide N-acetylgalactosaminyltransferase 12 |
| 243444_at | SRD5A3 | 1.70E-02 | -1.86 | steroid 5 alpha-reductase 3 |
| 232556_at | --- | 1.70E-02 | 1.71 | --- |
| 39249_at | AQP3 | 1.70E-02 | -2.18 | aquaporin 3 (Gill blood group) |
| 211734_s_at | FCER1A | 1.71E-02 | -2.21 | Fc fragment of IgE receptor Ia |
| 210360_s_at | MTSS1 | 1.71E-02 | 1.56 | MTSS I-BAR domain containing 1 |
| 239183_at | ANGPTL1 | 1.72E-02 | 1.37 | angiopoietin like 1 |
| 1553155_x_at | ATP6V0D2 | 1.72E-02 | -2.43 | ATPase H+ transporting V0 subunit d2 |
| 203609_s_at | ALDH5A1 | 1.73E-02 | -1.18 | aldehyde dehydrogenase 5 family member A1 |
| 226907_at | PPP1R14C | 1.73E-02 | -2.06 | protein phosphatase 1 regulatory inhibitor subunit 14C |
| 242366_at | --- | 1.74E-02 | -1.23 | --- |
| 64064_at | GIMAP5 /// GIMAP1-GIMAP5 | 1.74E-02 | 1.92 | GTPase, IMAP family member 5 /// GIMAP1-GIMAP5 readthrough |
| 221002_s_at | TSPAN14 | 1.75E-02 | 1.46 | tetraspanin 14 |
| 208699_x_at | TKT | 1.75E-02 | -1.32 | transketolase |
| 233208_x_at | CPSF2 | 1.75E-02 | -1.35 | cleavage and polyadenylation specific factor 2 |
| 209637_s_at | RGS12 | 1.76E-02 | -1.77 | regulator of G protein signaling 12 |
| 213100_at | UNC5B | 1.77E-02 | -1.41 | unc-5 netrin receptor B |
| 236003_x_at | --- | 1.77E-02 | 1.48 | --- |
| 230323_s_at | TMEM45B | 1.77E-02 | -2.00 | transmembrane protein 45B |
| 228175_at | SLC4A8 | 1.79E-02 | -1.61 | solute carrier family 4 member 8 |
| 1568964_x_at | SPN | 1.79E-02 | -1.59 | sialophorin |
| 226136_at | GLIPR1 | 1.80E-02 | -1.32 | GLI pathogenesis related 1 |
| 228557_at | L3MBTL4 | 1.82E-02 | 1.85 | L3MBTL histone methyl-lysine binding protein 4 |
| 43977_at | TMEM161A | 1.82E-02 | -1.22 | transmembrane protein 161A |
| 237398_at | --- | 1.82E-02 | 1.39 | --- |
| 208943_s_at | SEC62 | 1.83E-02 | -1.20 | SEC62 homolog, preprotein translocation factor |
| 1553713_a_at | RHEBL1 | 1.84E-02 | 1.51 | RHEB like 1 |
| 226727_at | CISD3 | 1.84E-02 | -1.42 | CDGSH iron sulfur domain 3 |
| 212281_s_at | TMEM97 | 1.84E-02 | -1.99 | transmembrane protein 97 |
| 1563696_at | HSD17B4 | 1.86E-02 | 2.08 | hydroxysteroid 17-beta dehydrogenase 4 |
| 228622_s_at | DNAJC4 | 1.87E-02 | -1.31 | DnaJ heat shock protein family (Hsp40) member C4 |
| 231247_s_at | --- | 1.87E-02 | 1.62 | --- |
| 222764_at | ASRGL1 | 1.87E-02 | -1.80 | asparaginase like 1 |
| 218885_s_at | GALNT12 | 1.88E-02 | -2.22 | polypeptide N-acetylgalactosaminyltransferase 12 |
| 224063_at | NLN | 1.88E-02 | 1.90 | neurolysin |
| 1563034_at | GPD1 | 1.88E-02 | -1.28 | glycerol-3-phosphate dehydrogenase 1 |
| 206167_s_at | ARHGAP6 | 1.90E-02 | -1.83 | Rho GTPase activating protein 6 |
| 1553142_at | LACC1 | 1.90E-02 | -1.79 | laccase domain containing 1 |
| 203665_at | HMOX1 | 1.90E-02 | -1.80 | heme oxygenase 1 |
| 212099_at | RHOB | 1.90E-02 | 1.19 | ras homolog family member B |
| 226857_at | ARHGEF19 | 1.90E-02 | 1.24 | Rho guanine nucleotide exchange factor 19 |
| 212271_at | MAPK1 | 1.91E-02 | -1.23 | mitogen-activated protein kinase 1 |
| 228281_at | DDIAS | 1.93E-02 | -1.70 | DNA damage induced apoptosis suppressor |
| 214146_s_at | PPBP | 1.93E-02 | -4.01 | pro-platelet basic protein |
| 227383_at | LINC00869 | 1.93E-02 | 1.52 | long intergenic non-protein coding RNA 869 |
| 243687_at | LINC00881 | 1.93E-02 | -1.46 | long intergenic non-protein coding RNA 881 |
| 228775_at | EMC3 | 1.93E-02 | -1.37 | ER membrane protein complex subunit 3 |
| 1553153_at | ATP6V0D2 | 1.97E-02 | -2.38 | ATPase H+ transporting V0 subunit d2 |
| 214597_at | SSTR2 | 1.99E-02 | 2.21 | somatostatin receptor 2 |
| 206022_at | NDP | 2.02E-02 | 2.86 | norrin cystine knot growth factor NDP |
| 243210_at | --- | 2.03E-02 | 1.55 | --- |
| 237444_at | KIF13A | 2.08E-02 | -1.25 | kinesin family member 13A |
| 223971_at | OR2A7 /// OR2A20P /// OR2A9P | 2.09E-02 | -2.18 | olfactory receptor family 2 subfamily A member 7 /// olfactory receptor family 2 subfamily A member 20 pseudogene /// olfactory receptor family 2 subfamily A member 9 pseudogene |
| 211709_s_at | CLEC11A | 2.09E-02 | -1.69 | C-type lectin domain containing 11A |
| 50314_i_at | C20orf27 | 2.11E-02 | -1.33 | chromosome 20 open reading frame 27 |
| 212681_at | EPB41L3 | 2.11E-02 | -1.32 | erythrocyte membrane protein band 4.1 like 3 |
| 229067_at | --- | 2.14E-02 | 1.44 | --- |
| 204445_s_at | ALOX5 | 2.16E-02 | -1.35 | arachidonate 5-lipoxygenase |
| 216250_s_at | LPXN | 2.17E-02 | -1.32 | leupaxin |
| 239544_at | --- | 2.22E-02 | 1.70 | --- |
| 224774_s_at | NAV1 | 2.26E-02 | 1.38 | neuron navigator 1 |
| 207892_at | CD40LG | 2.26E-02 | 1.40 | CD40 ligand |
| 214469_at | HIST1H2AE | 2.26E-02 | 2.08 | histone cluster 1 H2A family member e |
| 202701_at | BMP1 | 2.26E-02 | -1.30 | bone morphogenetic protein 1 |
| 234342_at | FAM20C | 2.28E-02 | -1.19 | FAM20C golgi associated secretory pathway kinase |
| 203747_at | AQP3 | 2.28E-02 | -2.06 | aquaporin 3 (Gill blood group) |
| 1566766_a_at | MACC1 | 2.29E-02 | -2.33 | MET transcriptional regulator MACC1 |
| 204222_s_at | GLIPR1 | 2.29E-02 | -1.28 | GLI pathogenesis related 1 |
| 218581_at | ABHD4 | 2.29E-02 | -1.38 | abhydrolase domain containing 4 |
| 218805_at | GIMAP5 /// GIMAP1-GIMAP5 | 2.29E-02 | 1.87 | GTPase, IMAP family member 5 /// GIMAP1-GIMAP5 readthrough |
| 202039_at | TIAF1 /// MYO18A | 2.36E-02 | -1.31 | TGFB1-induced anti-apoptotic factor 1 /// myosin XVIIIA |
| 209015_s_at | DNAJB6 | 2.39E-02 | -1.20 | DnaJ heat shock protein family (Hsp40) member B6 |
| 237544_at | --- | 2.43E-02 | 1.89 | --- |
| 203619_s_at | FAIM2 | 2.43E-02 | -2.23 | Fas apoptotic inhibitory molecule 2 |
| 218745_x_at | TMEM161A | 2.45E-02 | -1.25 | transmembrane protein 161A |
| 221757_at | PIK3IP1 | 2.46E-02 | 1.63 | phosphoinositide-3-kinase interacting protein 1 |
| 225630_at | EEPD1 | 2.46E-02 | -1.52 | endonuclease/exonuclease/phosphatase family domain containing 1 |
| 38037_at | HBEGF | 2.46E-02 | -1.43 | heparin binding EGF like growth factor |
| 213384_x_at | PLCB3 | 2.46E-02 | -1.18 | phospholipase C beta 3 |
| 209827_s_at | IL16 | 2.46E-02 | -1.39 | interleukin 16 |
| 201344_at | UBE2D2 | 2.46E-02 | -1.27 | ubiquitin conjugating enzyme E2 D2 |
| 205692_s_at | CD38 | 2.46E-02 | 2.04 | CD38 molecule |
| 225136_at | PLEKHA2 | 2.46E-02 | 1.17 | pleckstrin homology domain containing A2 |
| 214040_s_at | GSN | 2.48E-02 | -1.51 | gelsolin |
| 238026_at | RPL35A | 2.49E-02 | -1.38 | ribosomal protein L35a |
| 228443_s_at | KMT5A | 2.49E-02 | -1.17 | lysine methyltransferase 5A |
| 232136_s_at | CTTNBP2 | 2.49E-02 | -1.99 | cortactin binding protein 2 |
| 1558706_a_at | ATOH8 | 2.49E-02 | 2.55 | atonal bHLH transcription factor 8 |
| 227840_at | C2orf76 | 2.50E-02 | -1.22 | chromosome 2 open reading frame 76 |
| 237890_at | --- | 2.51E-02 | 1.88 | --- |
| 236293_at | RHOH | 2.51E-02 | 2.14 | ras homolog family member H |
| 231192_at | LPAR3 | 2.51E-02 | 2.14 | lysophosphatidic acid receptor 3 |
| 235072_s_at | KIF13A | 2.51E-02 | -1.27 | kinesin family member 13A |
| 200993_at | IPO7 | 2.51E-02 | -1.31 | importin 7 |
| 230252_at | LPAR5 | 2.52E-02 | 2.04 | lysophosphatidic acid receptor 5 |
| 243037_at | --- | 2.52E-02 | 1.30 | --- |
| 202765_s_at | FBN1 | 2.53E-02 | 1.44 | fibrillin 1 |
| 232151_at | MACC1 | 2.53E-02 | -2.27 | MET transcriptional regulator MACC1 |
| 221752_at | SSH1 | 2.53E-02 | -1.30 | slingshot protein phosphatase 1 |
| 1562415_a_at | SPOCD1 | 2.55E-02 | -2.21 | SPOC domain containing 1 |
| 207375_s_at | IL15RA | 2.59E-02 | 1.86 | interleukin 15 receptor subunit alpha |
| 209344_at | TPM4 | 2.59E-02 | -1.17 | tropomyosin 4 |
| 235276_at | EPSTI1 | 2.59E-02 | 2.05 | epithelial stromal interaction 1 |
| 212298_at | NRP1 | 2.60E-02 | -1.83 | neuropilin 1 |
| 202434_s_at | CYP1B1 | 2.61E-02 | -1.25 | cytochrome P450 family 1 subfamily B member 1 |
| 206710_s_at | EPB41L3 | 2.63E-02 | -1.39 | erythrocyte membrane protein band 4.1 like 3 |
| 222530_s_at | MKKS | 2.64E-02 | -1.27 | McKusick-Kaufman syndrome |
| 202238_s_at | NNMT | 2.64E-02 | 1.86 | nicotinamide N-methyltransferase |
| 210783_x_at | CLEC11A | 2.65E-02 | -1.29 | C-type lectin domain containing 11A |
| 213488_at | SNED1 | 2.66E-02 | -1.28 | sushi, nidogen and EGF like domains 1 |
| 232757_at | --- | 2.67E-02 | 1.45 | --- |
| 237495_at | MPP7 | 2.68E-02 | 1.38 | membrane palmitoylated protein 7 |
| 223312_at | PRADC1 | 2.69E-02 | -1.49 | protease associated domain containing 1 |
| 227943_at | --- | 2.69E-02 | 1.87 | --- |
| 228922_at | SHF | 2.69E-02 | -1.37 | Src homology 2 domain containing F |
| 239469_at | --- | 2.69E-02 | 1.35 | --- |
| 225495_x_at | GADD45GIP1 | 2.71E-02 | -1.30 | GADD45G interacting protein 1 |
| 229417_at | --- | 2.72E-02 | -1.29 | --- |
| 206955_at | AQP7 /// LOC100509620 | 2.73E-02 | -1.55 | aquaporin 7 /// putative aquaporin-7-like protein 3 |
| 205149_s_at | CLCN4 | 2.75E-02 | -2.00 | chloride voltage-gated channel 4 |
| 238600_at | JAKMIP1 | 2.77E-02 | 1.29 | janus kinase and microtubule interacting protein 1 |
| 209926_at | BORCS8 | 2.79E-02 | -1.16 | BLOC-1 related complex subunit 8 |
| 1561666_a_at | PSMG3-AS1 | 2.79E-02 | -1.26 | PSMG3 antisense RNA 1 (head to head) |
| 229150_at | --- | 2.79E-02 | -1.46 | --- |
| 232784_at | --- | 2.79E-02 | 1.59 | --- |
| 237031_at | --- | 2.81E-02 | -1.29 | --- |
| 212617_at | ZNF609 | 2.81E-02 | -1.29 | zinc finger protein 609 |
| 225665_at | MAP3K20 | 2.81E-02 | -1.35 | mitogen-activated protein kinase kinase kinase 20 |
| 227399_at | VGLL3 | 2.82E-02 | 3.60 | vestigial like family member 3 |
| 239585_at | --- | 2.84E-02 | 1.53 | --- |
| 226164_x_at | RIMKLB | 2.86E-02 | 1.43 | ribosomal modification protein rimK like family member B |
| 227979_at | RBM4 | 2.86E-02 | 1.37 | RNA binding motif protein 4 |
| 1556352_at | --- | 2.87E-02 | 2.06 | --- |
| 218717_s_at | P3H2 | 2.87E-02 | -1.59 | prolyl 3-hydroxylase 2 |
| 204806_x_at | HLA-F | 2.88E-02 | 1.15 | major histocompatibility complex, class I, F |
| 219348_at | USE1 | 2.94E-02 | -1.31 | unconventional SNARE in the ER 1 |
| 233936_s_at | GGNBP2 | 2.94E-02 | 1.22 | gametogenetin binding protein 2 |
| 226541_at | FBXO30 | 2.94E-02 | -1.50 | F-box protein 30 |
| 205386_s_at | MDM2 | 2.99E-02 | 1.48 | MDM2 proto-oncogene |
| 1570253_a_at | RHEBL1 | 2.99E-02 | 1.52 | RHEB like 1 |
| 205987_at | CD1C | 2.99E-02 | -2.06 | CD1c molecule |
| 221511_x_at | CCPG1 /// DNAAF4-CCPG1 | 3.00E-02 | -1.33 | cell cycle progression 1 /// DNAAF4-CCPG1 readthrough (NMD candidate) |
| 236417_at | --- | 3.02E-02 | 1.49 | --- |
| 237099_at | BPIFA2 | 3.02E-02 | -1.22 | BPI fold containing family A member 2 |
| 203036_s_at | MTSS1 | 3.05E-02 | 1.72 | MTSS I-BAR domain containing 1 |
| 230188_at | NIPAL4 | 3.05E-02 | 2.04 | NIPA like domain containing 4 |
| 1561348_at | LOC339874 | 3.06E-02 | 1.52 | uncharacterized LOC339874 |
| 1560928_at | --- | 3.08E-02 | 1.62 | --- |
| 1554534_at | DPYD | 3.10E-02 | 1.55 | dihydropyrimidine dehydrogenase |
| 224916_at | TMEM173 | 3.10E-02 | -1.60 | transmembrane protein 173 |
| 226222_at | RIC1 | 3.10E-02 | 1.25 | RIC1 homolog, RAB6A GEF complex partner 1 |
| 205158_at | RNASE4 | 3.10E-02 | 2.01 | ribonuclease A family member 4 |
| 202796_at | SYNPO | 3.10E-02 | 1.63 | synaptopodin |
| 236067_at | --- | 3.10E-02 | 1.55 | --- |
| 220599_s_at | CARD14 | 3.11E-02 | -1.36 | caspase recruitment domain family member 14 |
| 225715_at | RPTOR | 3.11E-02 | -1.31 | regulatory associated protein of MTOR complex 1 |
| 222067_x_at | HIST1H2BD | 3.13E-02 | 1.67 | histone cluster 1 H2B family member d |
| 202417_at | KEAP1 | 3.14E-02 | -1.40 | kelch like ECH associated protein 1 |
| 230524_at | MPI | 3.15E-02 | -1.29 | mannose phosphate isomerase |
| 226658_at | PDPN | 3.17E-02 | -2.05 | podoplanin |
| 1570121_at | ZNF365 | 3.17E-02 | -1.46 | zinc finger protein 365 |
| 1555758_a_at | CDKN3 | 3.18E-02 | -1.85 | cyclin dependent kinase inhibitor 3 |
| 232994_s_at | ARHGEF28 | 3.18E-02 | -1.93 | Rho guanine nucleotide exchange factor 28 |
| 230599_at | --- | 3.18E-02 | 1.42 | --- |
| 238482_at | KLF7 | 3.18E-02 | 1.46 | Kruppel like factor 7 |
| 233538_s_at | CYBB | 3.19E-02 | -1.31 | cytochrome b-245 beta chain |
| 205315_s_at | SNTB2 | 3.19E-02 | 1.30 | syntrophin beta 2 |
| 243006_at | --- | 3.22E-02 | 1.59 | --- |
| 1568957_x_at | SRGAP2 /// SRGAP2B /// SRGAP2C | 3.22E-02 | 1.44 | SLIT-ROBO Rho GTPase activating protein 2 /// SLIT-ROBO Rho GTPase activating protein 2B /// SLIT-ROBO Rho GTPase activating protein 2C |
| 222156_x_at | CCPG1 /// DNAAF4-CCPG1 | 3.22E-02 | -1.37 | cell cycle progression 1 /// DNAAF4-CCPG1 readthrough (NMD candidate) |
| 226301_at | SLC18B1 | 3.24E-02 | 1.87 | solute carrier family 18 member B1 |
| 228586_at | ENG | 3.24E-02 | -1.40 | endoglin |
| 205823_at | RGS12 | 3.24E-02 | -1.52 | regulator of G protein signaling 12 |
| 206682_at | CLEC10A | 3.24E-02 | -1.49 | C-type lectin domain containing 10A |
| 202708_s_at | HIST2H2BE | 3.25E-02 | 1.42 | histone cluster 2 H2B family member e |
| 221706_s_at | USE1 | 3.25E-02 | -1.32 | unconventional SNARE in the ER 1 |
| 235417_at | SPOCD1 | 3.25E-02 | -1.93 | SPOC domain containing 1 |
| 212316_at | NUP210 | 3.28E-02 | -1.44 | nucleoporin 210 |
| 1555022_at | RGS12 | 3.32E-02 | -1.58 | regulator of G protein signaling 12 |
| 222591_at | STYXL1 | 3.32E-02 | -1.31 | serine/threonine/tyrosine interacting like 1 |
| 202923_s_at | GCLC | 3.32E-02 | -1.58 | glutamate-cysteine ligase catalytic subunit |
| 205141_at | ANG | 3.33E-02 | 1.92 | angiogenin |
| 235568_at | MCEMP1 | 3.33E-02 | -1.33 | mast cell expressed membrane protein 1 |
| 223445_at | DTNBP1 | 3.34E-02 | 1.34 | dystrobrevin binding protein 1 |
| 208983_s_at | PECAM1 | 3.34E-02 | -1.33 | platelet and endothelial cell adhesion molecule 1 |
| 204297_at | PIK3C3 | 3.35E-02 | -1.13 | phosphatidylinositol 3-kinase catalytic subunit type 3 |
| 220558_x_at | TSPAN32 | 3.35E-02 | -1.31 | tetraspanin 32 |
| 235953_at | ZNF610 | 3.36E-02 | -1.40 | zinc finger protein 610 |
| 213868_s_at | DHRS7 | 3.36E-02 | -1.22 | dehydrogenase/reductase 7 |
| 236921_at | --- | 3.36E-02 | 1.80 | --- |
| 237173_at | LOC100132057 | 3.40E-02 | 1.51 | phosphodiesterase 4D interacting protein pseudogene |
| 237033_at | SHISAL2A | 3.40E-02 | 2.01 | shisa like 2A |
| 208916_at | SLC1A5 | 3.41E-02 | -1.41 | solute carrier family 1 member 5 |
| 203498_at | RCAN2 | 3.43E-02 | 1.87 | regulator of calcineurin 2 |
| 212020_s_at | MKI67 | 3.45E-02 | -1.42 | marker of proliferation Ki-67 |
| 203608_at | ALDH5A1 | 3.45E-02 | -2.35 | aldehyde dehydrogenase 5 family member A1 |
| 209420_s_at | SMPD1 | 3.45E-02 | 1.27 | sphingomyelin phosphodiesterase 1 |
| 209249_s_at | GHITM | 3.46E-02 | -1.12 | growth hormone inducible transmembrane protein |
| 202411_at | IFI27 | 3.47E-02 | 2.61 | interferon alpha inducible protein 27 |
| 205037_at | IFT27 | 3.48E-02 | -1.50 | intraflagellar transport 27 |
| 203037_s_at | MTSS1 | 3.48E-02 | 1.84 | MTSS I-BAR domain containing 1 |
| 210321_at | GZMH | 3.48E-02 | 2.06 | granzyme H |
| 235213_at | ITPKB | 3.48E-02 | 1.63 | inositol-trisphosphate 3-kinase B |
| 203821_at | HBEGF | 3.48E-02 | -1.39 | heparin binding EGF like growth factor |
| 223246_s_at | STRBP | 3.48E-02 | -1.37 | spermatid perinuclear RNA binding protein |
| 208109_s_at | LINC00597 | 3.48E-02 | 1.59 | long intergenic non-protein coding RNA 597 |
| 202307_s_at | TAP1 | 3.48E-02 | 1.51 | transporter 1, ATP binding cassette subfamily B member |
| 221898_at | PDPN | 3.49E-02 | -2.74 | podoplanin |
| 202587_s_at | AK1 | 3.54E-02 | -1.59 | adenylate kinase 1 |
| 215728_s_at | ACOT7 | 3.54E-02 | -1.44 | acyl-CoA thioesterase 7 |
| 210264_at | GPR35 | 3.54E-02 | -1.43 | G protein-coupled receptor 35 |
| 210743_s_at | CDC14A | 3.55E-02 | 1.40 | cell division cycle 14A |
| 238307_at | --- | 3.55E-02 | -1.91 | --- |
| 219686_at | STK32B | 3.55E-02 | -1.43 | serine/threonine kinase 32B |
| 212958_x_at | PAM | 3.56E-02 | -1.32 | peptidylglycine alpha-amidating monooxygenase |
| 204341_at | TRIM16 | 3.56E-02 | -1.76 | tripartite motif containing 16 |
| 38398_at | MADD | 3.56E-02 | 1.35 | MAP kinase activating death domain |
| 227805_at | METAP1D | 3.58E-02 | -1.54 | methionyl aminopeptidase type 1D, mitochondrial |
| 220153_at | ENTPD7 | 3.60E-02 | -1.46 | ectonucleoside triphosphate diphosphohydrolase 7 |
| 203740_at | MPHOSPH6 | 3.60E-02 | -1.39 | M-phase phosphoprotein 6 |
| 201507_at | PFDN1 | 3.60E-02 | -1.40 | prefoldin subunit 1 |
| 219451_at | MSRB2 | 3.62E-02 | 1.45 | methionine sulfoxide reductase B2 |
| 1553454_at | RPTN | 3.63E-02 | -3.08 | repetin |
| 213877_x_at | ELOB | 3.66E-02 | -1.25 | elongin B |
| 209716_at | CSF1 | 3.66E-02 | -1.65 | colony stimulating factor 1 |
| 209595_at | GTF2F2 | 3.66E-02 | -1.20 | general transcription factor IIF subunit 2 |
| 221087_s_at | APOL3 | 3.66E-02 | 1.97 | apolipoprotein L3 |
| 1555852_at | PSMB8-AS1 | 3.71E-02 | 1.57 | PSMB8 antisense RNA 1 (head to head) |
| 204497_at | ADCY9 | 3.71E-02 | -1.38 | adenylate cyclase 9 |
| 229872_s_at | LOC100996740 | 3.71E-02 | 1.32 | uncharacterized LOC100996740 |
| 223422_s_at | ARHGAP24 | 3.72E-02 | 1.42 | Rho GTPase activating protein 24 |
| 224507_s_at | MGC12916 | 3.72E-02 | 1.97 | uncharacterized protein MGC12916 |
| 243153_at | CDK5RAP2 | 3.72E-02 | -1.27 | CDK5 regulatory subunit associated protein 2 |
| 222571_at | ST6GALNAC6 | 3.73E-02 | -1.45 | ST6 N-acetylgalactosaminide alpha-2,6-sialyltransferase 6 |
| 221773_at | ELK3 | 3.74E-02 | 1.51 | ETS transcription factor ELK3 |
| 236489_at | ADGRF1 | 3.74E-02 | -3.11 | adhesion G protein-coupled receptor F1 |
| 222585_x_at | KRCC1 | 3.74E-02 | -1.25 | lysine rich coiled-coil 1 |
| 218300_at | PAGR1 | 3.76E-02 | -1.30 | PAXIP1 associated glutamate rich protein 1 |
| 223918_at | ACSL6 | 3.77E-02 | -1.57 | acyl-CoA synthetase long chain family member 6 |
| 205468_s_at | IRF5 | 3.77E-02 | -1.30 | interferon regulatory factor 5 |
| 1553644_at | SYNE3 | 3.79E-02 | 1.33 | spectrin repeat containing nuclear envelope family member 3 |
| 209544_at | RIPK2 | 3.79E-02 | 1.46 | receptor interacting serine/threonine kinase 2 |
| 242732_at | --- | 3.82E-02 | 2.28 | --- |
| 237308_at | --- | 3.84E-02 | -1.20 | --- |
| 202540_s_at | HMGCR | 3.84E-02 | -1.23 | 3-hydroxy-3-methylglutaryl-CoA reductase |
| 229581_at | ELFN1 | 3.85E-02 | -1.74 | extracellular leucine rich repeat and fibronectin type III domain containing 1 |
| 213947_s_at | NUP210 | 3.86E-02 | -1.46 | nucleoporin 210 |
| 210558_at | AKR1C4 | 3.88E-02 | -1.19 | aldo-keto reductase family 1 member C4 |
| 1552485_at | LACTB | 3.90E-02 | 1.39 | lactamase beta |
| 220288_at | MYO15A | 3.90E-02 | -1.29 | myosin XVA |
| 232053_x_at | RHBDD2 | 3.92E-02 | -1.35 | rhomboid domain containing 2 |
| 241751_at | OFD1 | 3.96E-02 | 1.41 | OFD1 centriole and centriolar satellite protein |
| 215398_at | --- | 3.97E-02 | 1.48 | --- |
| 1555967_at | --- | 3.97E-02 | 1.61 | --- |
| 1568955_at | SRGAP2 /// SRGAP2B /// SRGAP2C | 3.97E-02 | 1.43 | SLIT-ROBO Rho GTPase activating protein 2 /// SLIT-ROBO Rho GTPase activating protein 2B /// SLIT-ROBO Rho GTPase activating protein 2C |
| 227782_at | ZBTB7C | 3.97E-02 | -2.02 | zinc finger and BTB domain containing 7C |
| 242488_at | CHRM3 | 3.97E-02 | -1.52 | cholinergic receptor muscarinic 3 |
| 238638_at | SLC37A2 | 3.97E-02 | -1.53 | solute carrier family 37 member 2 |
| 236791_at | --- | 3.97E-02 | 1.43 | --- |
| 221030_s_at | ARHGAP24 | 3.97E-02 | 1.51 | Rho GTPase activating protein 24 |
| 204188_s_at | RARG | 3.99E-02 | -1.19 | retinoic acid receptor gamma |
| 1565743_at | N4BP2L2 | 3.99E-02 | 1.47 | NEDD4 binding protein 2 like 2 |
| 1552790_a_at | SEC62 | 4.00E-02 | -1.49 | SEC62 homolog, preprotein translocation factor |
| 226474_at | NLRC5 | 4.01E-02 | 1.65 | NLR family CARD domain containing 5 |
| 210252_s_at | MADD | 4.01E-02 | 1.38 | MAP kinase activating death domain |
| 239720_at | ZGLP1 | 4.01E-02 | -1.32 | zinc finger GATA like protein 1 |
| 219761_at | CLEC1A | 4.01E-02 | 1.63 | C-type lectin domain family 1 member A |
| 241905_at | PIK3C2A | 4.01E-02 | 1.44 | phosphatidylinositol-4-phosphate 3-kinase catalytic subunit type 2 alpha |
| 244697_at | --- | 4.01E-02 | 2.53 | --- |
| 221978_at | HLA-F | 4.08E-02 | 1.82 | major histocompatibility complex, class I, F |
| 209502_s_at | BAIAP2 | 4.08E-02 | -1.35 | BAI1 associated protein 2 |
| 227463_at | ACE | 4.08E-02 | -1.57 | angiotensin I converting enzyme |
| 239731_at | --- | 4.11E-02 | -1.44 | --- |
| 221828_s_at | MVB12B | 4.11E-02 | 1.23 | multivesicular body subunit 12B |
| 211188_at | CD84 | 4.12E-02 | 1.48 | CD84 molecule |
| 235076_at | CALCOCO2 | 4.12E-02 | 1.35 | calcium binding and coiled-coil domain 2 |
| 236533_at | ASAP1 | 4.18E-02 | -1.33 | ArfGAP with SH3 domain, ankyrin repeat and PH domain 1 |
| 209324_s_at | RGS16 | 4.18E-02 | 1.44 | regulator of G protein signaling 16 |
| 210442_at | IL1RL1 | 4.18E-02 | 2.22 | interleukin 1 receptor like 1 |
| 209301_at | CA2 | 4.18E-02 | -1.67 | carbonic anhydrase 2 |
| 212702_s_at | BICD2 | 4.18E-02 | -1.24 | BICD cargo adaptor 2 |
| 206366_x_at | XCL1 | 4.18E-02 | 1.86 | X-C motif chemokine ligand 1 |
| 216733_s_at | GATM | 4.18E-02 | -1.49 | glycine amidinotransferase |
| 226771_at | ATP8B2 | 4.20E-02 | 1.42 | ATPase phospholipid transporting 8B2 |
| 204043_at | TCN2 | 4.20E-02 | 1.88 | transcobalamin 2 |
| 207426_s_at | TNFSF4 | 4.21E-02 | 1.67 | TNF superfamily member 4 |
| 233626_at | --- | 4.24E-02 | -1.49 | --- |
| 224433_s_at | DDX54 | 4.24E-02 | -1.24 | DEAD-box helicase 54 |
| 207085_x_at | CSF2RA | 4.24E-02 | -1.32 | colony stimulating factor 2 receptor alpha subunit |
| 201627_s_at | INSIG1 | 4.24E-02 | -1.45 | insulin induced gene 1 |
| 202042_at | HARS | 4.24E-02 | -1.18 | histidyl-tRNA synthetase |
| 225656_at | EFHC1 | 4.24E-02 | 1.39 | EF-hand domain containing 1 |
| 220012_at | ERO1B | 4.24E-02 | 1.35 | endoplasmic reticulum oxidoreductase 1 beta |
| 202481_at | DHRS3 | 4.24E-02 | -1.64 | dehydrogenase/reductase 3 |
| 236380_at | RPP14 | 4.24E-02 | -1.42 | ribonuclease P/MRP subunit p14 |
| 1564656_at | --- | 4.26E-02 | 1.74 | --- |
| 209237_s_at | SLC23A2 | 4.26E-02 | -1.44 | solute carrier family 23 member 2 |
| 228945_s_at | SLC39A8 | 4.26E-02 | 1.60 | solute carrier family 39 member 8 |
| 239064_at | --- | 4.26E-02 | -1.29 | --- |
| 211577_s_at | IGF1 | 4.26E-02 | -1.30 | insulin like growth factor 1 |
| 243631_at | --- | 4.26E-02 | 1.34 | --- |
| 226844_at | MOB3B | 4.27E-02 | -1.55 | MOB kinase activator 3B |
| 225214_at | LOC100129034 | 4.28E-02 | -1.29 | uncharacterized LOC100129034 |
| 210615_at | NRP1 | 4.29E-02 | -1.26 | neuropilin 1 |
| 228205_at | TKT | 4.29E-02 | -1.35 | transketolase |
| 41856_at | UNC5B | 4.29E-02 | -1.22 | unc-5 netrin receptor B |
| 221521_s_at | GINS2 | 4.29E-02 | -1.46 | GINS complex subunit 2 |
| 205579_at | HRH1 | 4.29E-02 | -1.41 | histamine receptor H1 |
| 230315_at | --- | 4.29E-02 | 1.76 | --- |
| 230096_at | --- | 4.29E-02 | -1.68 | --- |
| 1553679_s_at | VKORC1L1 | 4.29E-02 | -1.42 | vitamin K epoxide reductase complex subunit 1 like 1 |
| 209225_x_at | TNPO1 | 4.30E-02 | -1.22 | transportin 1 |
| 228716_at | THRB | 4.30E-02 | 1.77 | thyroid hormone receptor beta |
| 211812_s_at | B3GALNT1 | 4.30E-02 | -1.59 | beta-1,3-N-acetylgalactosaminyltransferase 1 (globoside blood group) |
| 238031_at | LOC101928707 | 4.30E-02 | -1.31 | uncharacterized LOC101928707 |
| 225485_at | CEP41 | 4.31E-02 | -1.53 | centrosomal protein 41 |
| 207996_s_at | LDLRAD4 | 4.31E-02 | -1.58 | low density lipoprotein receptor class A domain containing 4 |
| 219890_at | CLEC5A | 4.34E-02 | -1.45 | C-type lectin domain containing 5A |
| 1566870_at | --- | 4.34E-02 | 1.22 | --- |
| 226020_s_at | OMA1 /// DAB1 | 4.34E-02 | -1.35 | OMA1 zinc metallopeptidase /// DAB adaptor protein 1 |
| 222757_s_at | MAP3K20 | 4.35E-02 | -1.48 | mitogen-activated protein kinase kinase kinase 20 |
| 232687_at | GPRIN3 | 4.36E-02 | -1.49 | GPRIN family member 3 |
| 225069_at | PCYT1A | 4.38E-02 | -1.14 | phosphate cytidylyltransferase 1, choline, alpha |
| 230283_at | NEURL2 | 4.38E-02 | 1.43 | neuralized E3 ubiquitin protein ligase 2 |
| 233630_at | CDS2 | 4.41E-02 | 1.47 | CDP-diacylglycerol synthase 2 |
| 203221_at | TLE1 | 4.42E-02 | 1.44 | TLE family member 1, transcriptional corepressor |
| 213565_s_at | SMAD6 | 4.43E-02 | -1.29 | SMAD family member 6 |
| 215802_at | --- | 4.43E-02 | 1.69 | --- |
| 237867_s_at | PID1 | 4.43E-02 | -1.66 | phosphotyrosine interaction domain containing 1 |
| 38241_at | BTN3A3 | 4.43E-02 | 1.55 | butyrophilin subfamily 3 member A3 |
| 235019_at | CPM | 4.43E-02 | 1.60 | carboxypeptidase M |
| 202075_s_at | PLTP | 4.44E-02 | 2.26 | phospholipid transfer protein |
| 223492_s_at | LRRFIP1 | 4.47E-02 | 1.37 | LRR binding FLII interacting protein 1 |
| 210817_s_at | CALCOCO2 | 4.49E-02 | 1.24 | calcium binding and coiled-coil domain 2 |
| 1554045_at | ZNF24 | 4.49E-02 | 1.39 | zinc finger protein 24 |
| 225156_at | ELOF1 | 4.49E-02 | -1.22 | elongation factor 1 homolog |
| 226134_s_at | MSI2 | 4.49E-02 | 1.53 | musashi RNA binding protein 2 |
| 228362_s_at | CALHM6 | 4.54E-02 | 1.83 | calcium homeostasis modulator family member 6 |
| 224646_x_at | H19 /// MIR675 | 4.55E-02 | -2.14 | H19 imprinted maternally expressed transcript /// microRNA 675 |
| 227560_at | SFXN2 | 4.55E-02 | -1.57 | sideroflexin 2 |
| 230795_at | --- | 4.56E-02 | 1.55 | --- |
| 243888_at | --- | 4.56E-02 | 1.61 | --- |
| 224200_s_at | RAD18 | 4.58E-02 | -1.29 | RAD18 E3 ubiquitin protein ligase |
| 202600_s_at | NRIP1 | 4.59E-02 | -1.52 | nuclear receptor interacting protein 1 |
| 218153_at | CARS2 | 4.59E-02 | -1.26 | cysteinyl-tRNA synthetase 2, mitochondrial |
| 202606_s_at | TLK1 | 4.59E-02 | -1.25 | tousled like kinase 1 |
| 206200_s_at | ANXA11 | 4.60E-02 | -1.16 | annexin A11 |
| 227228_s_at | CCDC88C | 4.60E-02 | 1.30 | coiled-coil domain containing 88C |
| 238379_x_at | --- | 4.60E-02 | -1.62 | --- |
| 227609_at | EPSTI1 | 4.61E-02 | 1.76 | epithelial stromal interaction 1 |
| 232670_at | --- | 4.62E-02 | 1.62 | --- |
| 207761_s_at | METTL7A | 4.66E-02 | 1.60 | methyltransferase like 7A |
| 1560348_at | ARHGEF28 | 4.66E-02 | -1.55 | Rho guanine nucleotide exchange factor 28 |
| 207849_at | IL2 | 4.69E-02 | 1.36 | interleukin 2 |
| 230108_at | ERCC6 | 4.70E-02 | -1.38 | ERCC excision repair 6, chromatin remodeling factor |
| 201266_at | TXNRD1 | 4.72E-02 | -1.25 | thioredoxin reductase 1 |
| 231277_x_at | DTWD2 | 4.72E-02 | -1.47 | DTW domain containing 2 |
| 205071_x_at | XRCC4 | 4.73E-02 | -1.25 | X-ray repair cross complementing 4 |
| 236341_at | CTLA4 | 4.73E-02 | 1.94 | cytotoxic T-lymphocyte associated protein 4 |
| 1562529_s_at | --- | 4.73E-02 | 1.68 | --- |
| 214567_s_at | XCL2 /// XCL1 | 4.74E-02 | 1.90 | X-C motif chemokine ligand 2 /// X-C motif chemokine ligand 1 |
| 234227_at | --- | 4.74E-02 | 1.58 | --- |
| 227762_at | --- | 4.74E-02 | 2.93 | --- |
| 1554486_a_at | GFOD1 | 4.74E-02 | 1.66 | glucose-fructose oxidoreductase domain containing 1 |
| 209993_at | ABCB1 | 4.75E-02 | -1.40 | ATP binding cassette subfamily B member 1 |
| 1556382_a_at | NAA15 | 4.76E-02 | 1.50 | N(alpha)-acetyltransferase 15, NatA auxiliary subunit |
| 222279_at | HLA-F-AS1 | 4.76E-02 | 1.44 | HLA-F antisense RNA 1 |
| 224348_s_at | HOTS | 4.76E-02 | -2.31 | H19 opposite tumor suppressor |
| 243885_x_at | --- | 4.77E-02 | 1.43 | --- |
| 219869_s_at | SLC39A8 | 4.77E-02 | 1.69 | solute carrier family 39 member 8 |
| 225999_at | RIMKLB | 4.77E-02 | 1.41 | ribosomal modification protein rimK like family member B |
| 212929_s_at | WASHC2C /// WASHC2A | 4.80E-02 | 1.19 | WASH complex subunit 2C /// WASH complex subunit 2A |
| 234011_at | --- | 4.80E-02 | 1.75 | --- |
| 205889_s_at | JAKMIP2 | 4.82E-02 | -2.17 | janus kinase and microtubule interacting protein 2 |
| 229543_at | CALHM6 | 4.83E-02 | 1.83 | calcium homeostasis modulator family member 6 |
| 1562250_at | --- | 4.83E-02 | 2.02 | --- |
| 209696_at | FBP1 | 4.86E-02 | -1.62 | fructose-bisphosphatase 1 |
| 228438_at | MSC-AS1 | 4.86E-02 | -1.91 | MSC antisense RNA 1 |
| 202503_s_at | PCLAF | 4.87E-02 | -2.02 | PCNA clamp associated factor |
| 220892_s_at | PSAT1 | 4.88E-02 | -1.23 | phosphoserine aminotransferase 1 |
| 218342_s_at | ERMP1 | 4.89E-02 | -1.60 | endoplasmic reticulum metallopeptidase 1 |
| 244461_at | SPECC1 | 4.89E-02 | -1.44 | sperm antigen with calponin homology and coiled-coil domains 1 |
| 219789_at | NPR3 | 4.89E-02 | 1.50 | natriuretic peptide receptor 3 |
| 222728_s_at | TAF1D /// SNORA8 /// SNORA1 /// SNORA18 /// SNORA40 /// SNORA32 /// SNORD5 /// MIR1304 | 4.89E-02 | 1.14 | TATA-box binding protein associated factor, RNA polymerase I subunit D /// small nucleolar RNA, H/ACA box 8 /// small nucleolar RNA, H/ACA box 1 /// small nucleolar RNA, H/ACA box 18 /// small nucleolar RNA, H/ACA box 40 /// small nucleolar RNA, H/ACA box 32 /// small nucleolar RNA, C/D box 5 /// microRNA 1304 |
| 218738_s_at | RNF138 | 4.89E-02 | 1.23 | ring finger protein 138 |
| 225662_at | MAP3K20 | 4.90E-02 | -1.35 | mitogen-activated protein kinase kinase kinase 20 |
| 238897_at | LRRC28 | 4.92E-02 | -1.95 | leucine rich repeat containing 28 |
| 229396_at | OVOL1 | 4.93E-02 | -1.58 | ovo like transcriptional repressor 1 |
| 210163_at | CXCL11 | 4.93E-02 | 3.85 | C-X-C motif chemokine ligand 11 |
| 208523_x_at | HIST1H2BI /// HIST1H2BG /// HIST1H2BF /// HIST1H2BE /// HIST1H2BC | 4.95E-02 | 1.45 | histone cluster 1 H2B family member i /// histone cluster 1 H2B family member g /// histone cluster 1 H2B family member f /// histone cluster 1 H2B family member e /// histone cluster 1 H2B family member c |
| 203791_at | DMXL1 | 4.97E-02 | -1.41 | Dmx like 1 |
| 229390_at | CALHM6 | 4.97E-02 | 1.74 | calcium homeostasis modulator family member 6 |
| 1556567_at | NAP1L4 | 4.97E-02 | 1.28 | nucleosome assembly protein 1 like 4 |
| 208278_s_at | --- | 4.97E-02 | 1.53 | --- |
| 229780_at | MTSS1 | 4.97E-02 | 1.57 | MTSS I-BAR domain containing 1 |
| 243221_at | FAM20A | 4.97E-02 | 1.77 | FAM20A golgi associated secretory pathway pseudokinase |
| 204857_at | MAD1L1 | 4.97E-02 | -1.43 | mitotic arrest deficient 1 like 1 |
| 205469_s_at | IRF5 | 4.99E-02 | -1.70 | interferon regulatory factor 5 |
| 212665_at | TIPARP | 4.99E-02 | -1.48 | TCDD inducible poly(ADP-ribose) polymerase |

***TABLE S3*** *Top 20 significant Gene Ontology (GO) biological processes (BP), GO molecular functions (MF), GO cellular components (CC), KEGG pathways and Wiki pathways identified by functional enrichment analysis of the significant (pFDR<0.05) differentially expressed genes (DEGs) in sputum cells between ex and current smokers (primary analysis; baseline samples). *Molecules from the Gene list that are annotated to the functional group. Abbreviation: pFDR, adjusted p-value*

| Entity | Description | | pFDR | *Genes |
| --- | --- | --- | --- | --- |
| GO:BP | cellular response to chemical stimulus | 3.10E-05 | | LRP5,GPR68,CYP1A1,PDE3B,RASAL1,AHRR,CYP1B1,NQO1,DTX1,CYGB,IL24,VCAM1,WFIKKN2,GCLM,IL19,ALDH3A1,CXCR5,FLT1,MGST1,P2RY6,IGF1,G6PD,RHOQ,COLEC12,GSR,SRXN1,SLC9A1,CDC37,VEGFD,MEF2A,SOCS1,GPX2,TALDO1,CACNB3,SPRY1,SLC7A11,IFNG,ABCB4,SAA2,GPD1,FLRT2,CD47,SMURF2,HAMP,ZBTB7B,ATP6V0D2,PRKCA,RAB7B,SERPINB2,PRLR,CCL24,FGFRL1,LDLRAP1,CYP27B1,NRP1,CXCL9,EVL,CSF1,FZD4,PIK3CG,SSH1,ADAM10,PELP1,S100B,DCSTAMP,PID1,HGF,BAIAP2,F13A1,HLAF,TBX21,KCNQ1,AQP3,TMEM161A,HMOX1,RHOB,MAPK1,PPBP,SSTR2,ALOX5,CD40LG,FAM20C,HBEGF,IL16,GSN,FBN1,IL15RA,MKKS,MDM2,TMEM173,CARD14,RPTOR,KEAP1,KLF7,CYBB,ENG,GCLC,DTNBP1,IFI27,ITPKB,GPR35,MADD,ELOB,GTF2F2,ADCY9,PAGR1,IRF5,RIPK2,HMGCR,AKR1C4,RHBDD2,CHRM3,RARG,NLRC5,PIK3C2A,IL1RL1,CA2,XCL1,TNFSF4,DDX54,CSF2RA,INSIG1,HRH1,VKORC1L1,THRB,LDLRAD4,SMAD6,NRIP1,IL2,TXNRD1,XRCC4,XCL2,FBP1,RNF138,CXCL11,TIPARP |
| GO:BP | positive regulation of multicellular organismal process | | 3.33E-05 | LRP5,GPR68,RASAL1,NPAS2,CD101,CYP1B1,CD226,FLT1,IGF1,EPHB2,OLIG2,KITLG,SLC9A1,ADORA2B,VEGFD,MEF2A,ATOH8,SOCS1,RETN,CACNB3,ALOX15B,LRRN3,STYXL1,SPRY1,IFNG,FLRT2,SMURF2,HAMP,CARD9,ZBTB7B,PRKCA,RAB7B,PRLR,ELOVL6,CCL24,LDLRAP1,CYP27B1,NRP1,SEMA4D,CSF1,FZD4,SSH1,S100B,DCSTAMP,PPP1R16B,HGF,BAIAP2,HLAF,TBX21,KCNQ1,SPN,HMOX1,RHOB,MAPK1,CD40LG,BMP1,FAM20C,HBEGF,CD38,RHOH,LPAR3,MKKS,ZNF609,TMEM173,PDPN,ZNF365,CYBB,ENG,ITPKB,TRIM16,IRF5,RIPK2,HMGCR,CHRM3,N4BP2L2,PIK3C2A,IL1RL1,CA2,XCL1,GATM,TNFSF4,HRH1,CLEC5A,OMA1,LRRFIP1,IL2 |
| GO:BP | response to organic substance | | 5.52E-05 | LRP5,GPR68,CYP1A1,PDE3B,CYP1B1,NQO1,DTX1,IL24,VCAM1,WFIKKN2,GCLM,IL19,ALDH3A1,CXCR5,FLT1,MGST1,P2RY6,IGF1,ABCC4,G6PD,RHOQ,COLEC12,UBD,NUB1,SLC9A1,CDC37,VEGFD,SOCS1,SLC1A2,RETN,TALDO1,SPRY1,SLC7A11,IFNG,ABCB4,GPD1,FLRT2,CD47,SMURF2,HAMP,CARD9,ZBTB7B,ATP6V0D2,PRKCA,RAB7B,SERPINB2,PRLR,CCL24,NNMT,FGFRL1,LDLRAP1,CYP27B1,NRP1,CXCL9,EVL,CSF1,FZD4,PIK3CG,SSH1,ADAM10,S100B,DCSTAMP,PID1,HGF,DNAJC4,BAIAP2,F13A1,ANG,HLAF,TBX21,KCNQ1,AQP3,TMEM161A,HMOX1,MAPK1,PPBP,SSTR2,ALOX5,CD40LG,FAM20C,IL16,CD38,GSN,FBN1,IL15RA,MKKS,MDM2,TMEM173,CARD14,RPTOR,KEAP1,KLF7,CYBB,ENG,GCLC,DTNBP1,PIK3C3,SMPD1,IFI27,GPR35,PAM,TRIM16,MADD,GTF2F2,ADCY9,PAGR1,IRF5,RIPK2,HMGCR,AKR1C4,RHBDD2,CHRM3,RARG,NLRC5,PIK3C2A,CALCOCO2,IL1RL1,CA2,XCL1,TNFSF4,DDX54,CSF2RA,INSIG1,HRH1,THRB,LDLRAD4,SMAD6,NRIP1,IL2,XCL2,RNF138,CXCL11,TIPARP |
| GO:BP | positive regulation of biological process | | 5.52E-05 | LRP5,GPR68,CYP1A1,RASAL1,AHRR,KLHL6,NPAS2,CD101,CYP1B1,NQO1,DTX1,IL24,VCAM1,LAMB1,ECSCR,SSBP3,CYTL1,IL19,ALDH3A1,CD226,CXCR5,FLT1,EOMES,ABCB1,SEMA6B,P2RY6,IGF1,STAC,ABCC4,G6PD,RHOQ,COLEC12,ITLN1,OSGIN1,EPHB2,OLIG2,UBD,KITLG,NUB1,SLC9A1,ACOX2,CDC37,PKIB,ADORA2B,VEGFD,MEF2A,ATOH8,CD1B,SOCS1,SLC1A2,RETN,CACNB3,PAG1,ALOX15B,CD1A,LRRN3,CLEC11A,STYXL1,SPRY1,CDK5RAP2,IFNG,ABCB4,GPD1,FLRT2,CD47,SMURF2,HAMP,CARD9,ZBTB7B,DOCK3,PRKCA,RAB7B,PRLR,ELOVL6,CCL24,C1S,PBX4,LDLRAP1,CYP27B1,NRP1,CXCL9,PRR16,SEMA4D,SEMA4C,EVL,TGFA,MACC1,SLC1A3,CSF1,EVC,SEPTIN9,FZD4,PIK3CG,SSH1,ADAM10,PELP1,RIPOR1,S100B,AP1AR,DCSTAMP,PID1,PPP1R16B,HGF,MAP3K20,BAIAP2,ZNF746,ANG,HLAF,TBX21,BTN3A1,KCNQ1,AQP3,TSPAN14,UNC5B,SPN,TMEM161A,RHEBL1,ARHGAP6,HMOX1,RHOB,ARHGEF19,MAPK1,PPBP,NDP,LPXN,CD40LG,BMP1,FAM20C,HBEGF,IL16,UBE2D2,CD38,GSN,RHOH,LPAR3,LPAR5,IL15RA,MKKS,MPP7,ZNF609,RBM4,MDM2,CD1C,CCPG1,TMEM173,SYNPO,CARD14,RPTOR,KEAP1,PDPN,ZNF365,KLF7,CYBB,ENG,CLEC10A,GCLC,DTNBP1,PECAM1,PIK3C3,SMPD1,ITPKB,GPR35,CDC14A,TRIM16,MADD,GTF2F2,APOL3,ADCY9,ELK3,ADGRF1,PAGR1,IRF5,RIPK2,HMGCR,ZBTB7C,CHRM3,RARG,N4BP2L2,NLRC5,PIK3C2A,ACE,CD84,CALCOCO2,ASAP1,IL1RL1,CA2,XCL1,GATM,TNFSF4,INSIG1,HRH1,THRB,CLEC5A,OMA1,TLE1,SMAD6,BTN3A3,PLTP,LRRFIP1,ZNF24,ELOF1,RAD18,NRIP1,IL2,ERCC6,CTLA4,XCL2,NAA15,WASHC2C,NPR3,TAF1D,OVOL1,CXCL11,FAM20A,TIPARP |
| GO:BP | response to cytokine | | 1.06E-04 | DTX1,IL24,VCAM1,GCLM,IL19,CXCR5,UBD,NUB1,CDC37,SOCS1,TALDO1,IFNG,GPD1,CD47,HAMP,PRKCA,RAB7B,SERPINB2,PRLR,CCL24,LDLRAP1,CYP27B1,CXCL9,EVL,CSF1,FZD4,ADAM10,DCSTAMP,PID1,HGF,F13A1,HLAF,HMOX1,MAPK1,PPBP,ALOX5,CD40LG,IL16,CD38,GSN,IL15RA,MKKS,TMEM173,CARD14,KEAP1,GCLC,IFI27,GPR35,MADD,IRF5,RIPK2,RARG,NLRC5,CALCOCO2,IL1RL1,XCL1,TNFSF4,CSF2RA,IL2,XCL2,RNF138,CXCL11 |
| GO:BP | pentose biosynthetic process | | 3.28E-04 | PGD,G6PD,TALDO1,TKT |
| GO:BP | response to chemical | | 3.28E-04 | LRP5,GPR68,CYP1A1,PDE3B,RASAL1,AHRR,NPAS2,CYP1B1,NQO1,DTX1,CYGB,IL24,VCAM1,ECSCR,WFIKKN2,GCLM,IL19,ALDH3A1,CXCR5,FLT1,ABCB1,SEMA6B,MGST1,P2RY6,IGF1,ABCC4,G6PD,RHOQ,COLEC12,GSR,SRXN1,EPHB2,UBD,NUB1,SLC9A1,CDC37,VEGFD,MEF2A,SOCS1,GPX2,SLC1A2,RETN,TALDO1,CACNB3,CHRNA1,SPRY1,SLC7A11,IFNG,ABCB4,SAA2,GPD1,FLRT2,CD47,SMURF2,HAMP,CARD9,ZBTB7B,ATP6V0D2,PRKCA,RAB7B,SERPINB2,PRLR,CCL24,NNMT,FGFRL1,LDLRAP1,CYP27B1,NRP1,CXCL9,SEMA4D,SEMA4C,EVL,TGFA,SLC1A3,CSF1,FZD4,PIK3CG,SSH1,ADAM10,PELP1,S100B,DCSTAMP,PID1,HGF,DNAJC4,BAIAP2,F13A1,TESMIN,ANG,HLAF,TBX21,KCNQ1,AQP3,UNC5B,SPN,TMEM161A,HMOX1,RHOB,MAPK1,PPBP,SSTR2,OR2A7,ALOX5,CD40LG,FAM20C,HBEGF,IL16,CD38,GSN,FBN1,IL15RA,MKKS,RBM4,MDM2,TMEM173,CARD14,RPTOR,KEAP1,PDPN,KLF7,CYBB,ENG,GCLC,DTNBP1,PIK3C3,SLC1A5,SMPD1,IFI27,ITPKB,GPR35,PAM,TRIM16,MADD,ELOB,GTF2F2,ADCY9,PAGR1,IRF5,RIPK2,HMGCR,AKR1C4,RHBDD2,CHRM3,RARG,NLRC5,PIK3C2A,CALCOCO2,IL1RL1,CA2,XCL1,TCN2,TNFSF4,DDX54,CSF2RA,INSIG1,HRH1,VKORC1L1,THRB,LDLRAD4,SMAD6,NRIP1,ANXA11,IL2,ERCC6,TXNRD1,XRCC4,XCL2,FBP1,RNF138,CXCL11,TIPARP |
| GO:BP | regulation of multicellular organismal process | | 5.25E-04 | LRP5,GPR68,PDE3B,RASAL1,NPAS2,CD101,CYP1B1,DTX1,ECSCR,IL19,CD226,FLT1,MYOZ1,SEMA6B,IGF1,G6PD,EPHB2,OLIG2,KITLG,SLC9A1,ADORA2B,VEGFD,MEF2A,ATOH8,SOCS1,RETN,CACNB3,ALOX15B,LRRN3,STYXL1,SPRY1,CDK5RAP2,IFNG,FLRT2,CD47,SMURF2,HAMP,CARD9,ZBTB7B,PRKCA,RAB7B,SERPINB2,PRLR,ELOVL6,CCL24,LDLRAP1,CYP27B1,NRP1,DTX4,SEMA4D,SEMA4C,EVL,ACOT11,CSF1,FZD4,PIK3CG,SSH1,ADAM10,DCANP1,S100B,DCSTAMP,PPP1R16B,HGF,BAIAP2,HLAF,TBX21,BTN3A1,KCNQ1,AQP3,SPN,NLN,HMOX1,RHOB,ARHGEF19,MAPK1,SSTR2,CD40LG,BMP1,FAM20C,HBEGF,CD38,RHOH,LPAR3,FBN1,MKKS,ZNF609,MDM2,TMEM173,KEAP1,PDPN,ZNF365,KLF7,CYBB,SRGAP2,ENG,ITPKB,GPR35,TRIM16,IRF5,RIPK2,HMGCR,CHRM3,RARG,N4BP2L2,NLRC5,PIK3C2A,ACE,CD84,ASAP1,IL1RL1,CA2,XCL1,GATM,TNFSF4,DHRS3,HRH1,THRB,LDLRAD4,CLEC5A,OMA1,SMAD6,BTN3A3,LRRFIP1,IL2,CTLA4,NPR3 |
| GO:BP | cell surface receptor signaling pathway | | 5.65E-04 | LRP5,KLHL6,CD101,CYP1B1,DTX1,IL24,VCAM1,WFIKKN2,GCLM,IL19,CD226,CXCR5,FLT1,DAGLA,SEMA6B,P2RY6,IGF1,RHOQ,EPHB2,KITLG,CDC37,ADORA2B,VEGFD,ATOH8,SOCS1,TALDO1,CACNB3,PAG1,CHRNA1,MVB12B,SPRY1,IFNG,FLRT2,CD47,SMURF2,CARD9,ZBTB7B,ATP6V0D2,PRKCA,SERPINB2,PRLR,CCL24,FGFRL1,NRP1,DTX4,CXCL9,SEMA4D,SEMA4C,EVL,TGFA,CSF1,EVC,FZD4,SSH1,ADAM10,PID1,HGF,BAIAP2,F13A1,HLAF,BTN3A1,FCER1A,MTSS1,ANGPTL1,TSPAN14,UNC5B,HMOX1,ARHGEF19,MAPK1,PPBP,NDP,ALOX5,LPXN,CD40LG,FAM20C,FAIM2,HBEGF,PLCB3,IL16,CD38,FBN1,IL15RA,MKKS,GGNBP2,CARD14,ARHGEF28,CYBB,ENG,CLEC10A,GCLC,PECAM1,TSPAN32,IFI27,IFT27,ITPKB,GPR35,MADD,GTF2F2,ADGRF1,IRF5,RIPK2,RARG,NLRC5,CLEC1A,PIK3C2A,IL1RL1,XCL1,TNFSF4,CSF2RA,LDLRAD4,TLE1,SMAD6,BTN3A3,CCDC88C,IL2,ERCC6,CTLA4,XCL2,RNF138,CXCL11,TIPARP |
| GO:BP | cellular response to organic substance | | 7.22E-04 | LRP5,GPR68,CYP1A1,PDE3B,CYP1B1,DTX1,IL24,VCAM1,WFIKKN2,GCLM,IL19,CXCR5,FLT1,P2RY6,IGF1,RHOQ,COLEC12,SLC9A1,CDC37,VEGFD,SOCS1,TALDO1,SPRY1,IFNG,ABCB4,GPD1,FLRT2,CD47,SMURF2,HAMP,ZBTB7B,ATP6V0D2,PRKCA,RAB7B,SERPINB2,PRLR,CCL24,FGFRL1,LDLRAP1,CYP27B1,NRP1,CXCL9,EVL,CSF1,FZD4,PIK3CG,SSH1,DCSTAMP,PID1,HGF,BAIAP2,F13A1,HLAF,TBX21,KCNQ1,HMOX1,MAPK1,PPBP,SSTR2,ALOX5,CD40LG,FAM20C,IL16,GSN,FBN1,IL15RA,MKKS,MDM2,TMEM173,CARD14,RPTOR,KEAP1,KLF7,CYBB,ENG,GCLC,DTNBP1,IFI27,GPR35,MADD,GTF2F2,ADCY9,PAGR1,IRF5,RIPK2,HMGCR,AKR1C4,RHBDD2,CHRM3,RARG,NLRC5,PIK3C2A,IL1RL1,CA2,XCL1,TNFSF4,DDX54,CSF2RA,INSIG1,HRH1,THRB,LDLRAD4,SMAD6,NRIP1,IL2,XCL2,RNF138,CXCL11,TIPARP |
| GO:BP | regulation of response to stimulus | | 7.94E-04 | LRP5,GPR68,PDE3B,RASAL1,KLHL6,NPAS2,CYP1B1,DTX1,IL24,VCAM1,WFIKKN2,GCLM,IL19,CD226,FLT1,MYOZ1,ABCB1,SEMA6B,P2RY6,IGF1,G6PD,RHOQ,COLEC12,EPHB2,UBD,KITLG,GPAT3,SLC9A1,ACOX2,CDC37,ADORA2B,VEGFD,MEF2A,CD1B,SOCS1,RETN,CACNB3,PAG1,ALOX15B,CD1A,KMT5A,MVB12B,STYXL1,SPRY1,SLC7A11,IFNG,CD47,SMURF2,HAMP,DDIT4L,CARD9,ZBTB7B,PIK3IP1,PRKCA,RAB7B,SERPINB2,PRLR,CCL24,C1S,NUP210,LDLRAP1,CYP27B1,NRP1,SEMA4D,SEMA4C,TGFA,CSF1,EVC,FZD4,PIK3CG,SSH1,ADAM10,S100B,PID1,PPP1R16B,HGF,MAP3K20,RGS12,BAIAP2,HLAF,TBX21,BTN3A1,FCER1A,TSPAN14,UNC5B,TMEM161A,ARHGAP6,HMOX1,RHOB,ARHGEF19,MAPK1,DDIAS,LPXN,CD40LG,FAM20C,DNAJB6,FAIM2,HBEGF,IL16,UBE2D2,CD38,GSN,RHOH,LPAR3,FBN1,MKKS,MPP7,GGNBP2,MDM2,CD1C,TMEM173,CARD14,RPTOR,PDPN,ZNF365,ARHGEF28,KLF7,SRGAP2,ENG,CLEC10A,GCLC,DTNBP1,PECAM1,PIK3C3,TSPAN32,RCAN2,SMPD1,ITPKB,GPR35,TRIM16,MADD,APOL3,ARHGAP24,PAGR1,RIPK2,HMGCR,NLRC5,CD84,RGS16,IL1RL1,XCL1,TNFSF4,INSIG1,DHRS3,MOB3B,LDLRAD4,TLE1,SMAD6,BTN3A3,CCDC88C,IL2,ERCC6,CTLA4,XCL2,FBP1 |
| GO:BP | signal transduction | | 1.09E-03 | LRP5,GPR68,PDE3B,RASAL1,KLHL6,CD101,CYP1B1,DTX1,IL24,VCAM1,WFIKKN2,GCLM,CYTL1,IL19,CD226,CXCR5,FLT1,MYOZ1,DAGLA,SEMA6B,P2RY6,IGF1,STAC,RHOQ,COLEC12,OSGIN1,EPHB2,UBD,KITLG,GPAT3,SLC9A1,CDC37,ADORA2B,VEGFD,MEF2A,ATOH8,DTNA,SOCS1,RETN,TALDO1,CACNB3,PAG1,ALOX15B,CHRNA1,KMT5A,MVB12B,CLEC11A,STYXL1,SPRY1,IFNG,FLRT2,CD47,SMURF2,HAMP,DDIT4L,CARD9,ZBTB7B,DOCK3,PIK3IP1,TIAF1,ATP6V0D2,PRKCA,IPO7,RAB7B,SERPINB2,PRLR,CCL24,FGFRL1,LDLRAP1,CYP27B1,NRP1,DTX4,CXCL9,SEMA4D,SEMA4C,EVL,TGFA,ACOT11,MACC1,CSF1,EVC,FZD4,PIK3CG,SSH1,ADAM10,RIPOR1,S100B,PID1,PPP1R16B,HGF,MAP3K20,RGS12,BAIAP2,F13A1,HLAF,BTN3A1,FCER1A,MTSS1,ANGPTL1,TSPAN14,UNC5B,SPN,TMEM161A,RHEBL1,ARHGAP6,HMOX1,RHOB,ARHGEF19,MAPK1,DDIAS,PPBP,SSTR2,NDP,OR2A7,ALOX5,LPXN,CD40LG,BMP1,FAM20C,FAIM2,HBEGF,PLCB3,IL16,UBE2D2,CD38,GSN,RHOH,LPAR3,LPAR5,FBN1,IL15RA,MKKS,MPP7,GGNBP2,MDM2,CARD14,RPTOR,PDPN,ARHGEF28,CYBB,SRGAP2,ENG,CLEC10A,GCLC,DTNBP1,PECAM1,PIK3C3,TSPAN32,RCAN2,SMPD1,IFI27,IFT27,GZMH,ITPKB,GPR35,STK32B,TRIM16,MADD,GTF2F2,APOL3,ADCY9,ARHGAP24,ELK3,ADGRF1,PAGR1,IRF5,RIPK2,HMGCR,RHBDD2,CHRM3,RARG,NLRC5,CLEC1A,PIK3C2A,RGS16,IL1RL1,CA2,XCL1,TNFSF4,DDX54,CSF2RA,INSIG1,DHRS3,MOB3B,HRH1,THRB,LDLRAD4,CLEC5A,NEURL2,TLE1,SMAD6,BTN3A3,NRIP1,TLK1,CCDC88C,IL2,ERCC6,TXNRD1,CTLA4,XCL2,FBP1,NPR3,RNF138,CXCL11,TIPARP |
| GO:BP | response to stimulus | | 1.09E-03 | LRP5,GPR68,CYP1A1,PDE3B,RASAL1,AHRR,KLHL6,NPAS2,CD101,CYP1B1,NQO1,DTX1,CYGB,IL24,VCAM1,ECSCR,WFIKKN2,GCLM,CYTL1,IL19,ALDH3A1,CD226,CXCR5,FLT1,PCARE,EOMES,FUCA1,MYOZ1,ABCB1,DAGLA,SEMA6B,MGST1,P2RY6,IGF1,STAC,ABCC4,G6PD,RHOQ,COLEC12,PLA2G2D,ITLN1,GSR,SRXN1,OSGIN1,EPHB2,PTGFRN,UBD,KITLG,NUB1,GPAT3,SLC9A1,ACOX2,CDC37,ADORA2B,VEGFD,MEF2A,ATOH8,DTNA,CD1B,SOCS1,GPX2,FCMR,SLC1A2,RETN,TALDO1,CACNB3,PAG1,ALOX15B,CD1A,CHRNA1,KMT5A,MVB12B,CLEC11A,STYXL1,SPRY1,SLC7A11,IFNG,ABCB4,SAA2,GPD1,FLRT2,CD47,SMURF2,HAMP,DDIT4L,CARD9,ZBTB7B,DOCK3,PIK3IP1,TIAF1,ATP6V0D2,PRKCA,IPO7,RAB7B,SERPINB2,PRLR,CCL24,NNMT,FGFRL1,C1S,NUP210,LDLRAP1,CYP27B1,NRP1,DTX4,PRCD,CXCL9,SEMA4D,SEMA4C,EVL,TGFA,ACOT11,MACC1,SLC1A3,CSF1,EVC,FZD4,PIK3CG,SSH1,ADAM10,DCANP1,PELP1,RIPOR1,S100B,DCSTAMP,PID1,PPP1R16B,HGF,MAP3K20,DNAJC4,RGS12,BAIAP2,F13A1,METTL7A,TESMIN,ANG,CD207,HLAF,TBX21,BTN3A1,KCNQ1,AQP3,FCER1A,MTSS1,ANGPTL1,TSPAN14,UNC5B,SPN,GLIPR1,TMEM161A,RHEBL1,ARHGAP6,HMOX1,RHOB,ARHGEF19,MAPK1,DDIAS,PPBP,SSTR2,NDP,OR2A7,ALOX5,LPXN,CD40LG,BMP1,FAM20C,DNAJB6,FAIM2,EEPD1,HBEGF,PLCB3,IL16,UBE2D2,CD38,GSN,RHOH,LPAR3,LPAR5,FBN1,IL15RA,MKKS,MPP7,RBM4,GGNBP2,MDM2,CD1C,BPIFA2,TMEM173,SYNPO,CARD14,RPTOR,KEAP1,PDPN,ZNF365,ARHGEF28,KLF7,CYBB,SRGAP2,ENG,CLEC10A,HIST2H2BE,GCLC,MCEMP1,DTNBP1,PECAM1,PIK3C3,TSPAN32,SLC1A5,RCAN2,SMPD1,IFI27,IFT27,GZMH,ITPKB,STRBP,TAP1,GPR35,STK32B,PAM,TRIM16,MADD,MSRB2,ELOB,GTF2F2,APOL3,ADCY9,ARHGAP24,ELK3,ADGRF1,PAGR1,IRF5,RIPK2,HMGCR,AKR1C4,RHBDD2,CHRM3,RARG,NLRC5,CLEC1A,PIK3C2A,CD84,CALCOCO2,RGS16,IL1RL1,CA2,XCL1,GATM,TCN2,TNFSF4,DDX54,CSF2RA,INSIG1,DHRS3,SLC23A2,MOB3B,GINS2,HRH1,VKORC1L1,THRB,LDLRAD4,CLEC5A,OMA1,NEURL2,TLE1,SMAD6,BTN3A3,RAD18,NRIP1,TLK1,ANXA11,CCDC88C,IL2,ERCC6,TXNRD1,XRCC4,CTLA4,XCL2,FBP1,PCLAF,NPR3,RNF138,CXCL11,FAM20A,TIPARP,H2BC10 |
| GO:BP | response to interferon-gamma | | 1.21E-03 | VCAM1,UBD,NUB1,CDC37,SOCS1,IFNG,CD47,RAB7B,CCL24,CYP27B1,EVL,HLAF,GSN,IRF5,NLRC5,CALCOCO2,XCL1,XCL2 |
| GO:BP | response to stress | | 1.21E-03 | GPR68,CYP1A1,NPAS2,CYP1B1,NQO1,CYGB,IL24,VCAM1,GCLM,ALDH3A1,CD226,MYOZ1,ABCB1,MGST1,IGF1,STAC,G6PD,COLEC12,PLA2G2D,GSR,SRXN1,EPHB2,PTGFRN,UBD,NUB1,SLC9A1,ACOX2,CDC37,ADORA2B,VEGFD,SOCS1,GPX2,FCMR,SLC1A2,KMT5A,SLC7A11,IFNG,SAA2,CD47,HAMP,CARD9,ZBTB7B,PRKCA,IPO7,RAB7B,SERPINB2,CCL24,C1S,NUP210,CYP27B1,NRP1,CXCL9,SEMA4C,EVL,TGFA,ACOT11,SLC1A3,CSF1,FZD4,PIK3CG,RIPOR1,S100B,HGF,MAP3K20,DNAJC4,F13A1,ANG,CD207,HLAF,AQP3,MTSS1,SPN,TMEM161A,HMOX1,RHOB,ARHGEF19,MAPK1,DDIAS,PPBP,SSTR2,ALOX5,CD40LG,DNAJB6,FAIM2,EEPD1,HBEGF,UBE2D2,CD38,GSN,LPAR5,RBM4,MDM2,BPIFA2,TMEM173,RPTOR,KEAP1,PDPN,ZNF365,CYBB,CLEC10A,HIST2H2BE,GCLC,DTNBP1,PECAM1,PIK3C3,TSPAN32,IFI27,TAP1,PAM,MSRB2,ELOB,APOL3,ELK3,PAGR1,IRF5,RIPK2,HMGCR,RHBDD2,NLRC5,CLEC1A,CD84,CALCOCO2,IL1RL1,CA2,XCL1,TNFSF4,INSIG1,SLC23A2,GINS2,HRH1,VKORC1L1,CLEC5A,SMAD6,RAD18,TLK1,CCDC88C,IL2,ERCC6,TXNRD1,XRCC4,CTLA4,XCL2,PCLAF,RNF138,CXCL11,H2BC10 |
| GO:BP | positive regulation of developmental process | | 1.29E-03 | LRP5,GPR68,RASAL1,CD101,CYP1B1,SSBP3,FLT1,IGF1,EPHB2,OLIG2,KITLG,VEGFD,MEF2A,ATOH8,SOCS1,RETN,ALOX15B,LRRN3,STYXL1,SPRY1,IFNG,FLRT2,SMURF2,HAMP,ZBTB7B,PRKCA,RAB7B,CCL24,CYP27B1,NRP1,CXCL9,SEMA4D,CSF1,FZD4,S100B,DCSTAMP,PPP1R16B,HGF,BAIAP2,TBX21,HMOX1,RHOB,MAPK1,BMP1,FAM20C,RHOH,LPAR3,MKKS,ZNF609,RBM4,MDM2,PDPN,ZNF365,CYBB,ENG,ITPKB,TRIM16,RIPK2,HMGCR,ZBTB7C,N4BP2L2,PIK3C2A,CA2,TNFSF4,IL2 |
| GO:BP | cellular response to cytokine stimulus | | 1.29E-03 | DTX1,IL24,VCAM1,GCLM,IL19,CXCR5,CDC37,SOCS1,TALDO1,IFNG,GPD1,CD47,HAMP,RAB7B,SERPINB2,PRLR,CCL24,LDLRAP1,CXCL9,EVL,CSF1,FZD4,DCSTAMP,PID1,HGF,F13A1,HLAF,HMOX1,MAPK1,PPBP,ALOX5,CD40LG,IL16,GSN,IL15RA,MKKS,TMEM173,CARD14,KEAP1,IFI27,GPR35,MADD,IRF5,RIPK2,RARG,NLRC5,IL1RL1,XCL1,TNFSF4,CSF2RA,IL2,XCL2,RNF138,CXCL11 |
| GO:BP | retina vasculature morphogenesis in camera-type eye | | 1.29E-03 | LRP5,CYP1B1,NRP1,FZD4,NDP |
| GO:BP | immune system process | | 1.37E-03 | LRP5,GPR68,KLHL6,CD101,DTX1,VCAM1,SSBP3,IL19,CD226,CXCR5,FLT1,EOMES,FUCA1,MGST1,IGF1,G6PD,COLEC12,PLA2G2D,ITLN1,UBD,KITLG,PIR,NUB1,CDC37,ADORA2B,VEGFD,CD1B,SOCS1,FCMR,RETN,CACNB3,PAG1,SH3PXD2A,CD1A,SLC7A11,IFNG,CD47,HAMP,GLO1,CARD9,ZBTB7B,PRKCA,IPO7,RAB7B,CCL24,SDC3,C1S,CYP27B1,CXCL9,SEMA4D,EVL,CSF1,PIK3CG,ADAM10,S100B,DCSTAMP,BAIAP2,METTL7A,CD207,HLAF,TBX21,BTN3A1,AQP3,FCER1A,TSPAN14,SPN,GLIPR1,HMOX1,MAPK1,PPBP,ALOX5,LPXN,CD40LG,FAM20C,IL16,UBE2D2,CD38,GSN,RHOH,FBN1,CD1C,BPIFA2,TMEM173,PDPN,CYBB,CLEC10A,HIST2H2BE,MCEMP1,PECAM1,PIK3C3,TSPAN32,IFI27,ITPKB,TAP1,IRF5,RIPK2,RARG,N4BP2L2,NLRC5,ACE,CD84,CALCOCO2,IL1RL1,CA2,XCL1,TNFSF4,HRH1,CLEC5A,SMAD6,BTN3A3,IL2,ERCC6,XRCC4,CTLA4,XCL2,CXCL11,MAD1L1,TIPARP,H2BC10 |
| GO:BP | positive regulation of immune system process | | 1.37E-03 | GPR68,KLHL6,CD101,VCAM1,CD226,IGF1,COLEC12,KITLG,ADORA2B,VEGFD,CD1B,SOCS1,CACNB3,PAG1,CD1A,IFNG,CD47,CARD9,ZBTB7B,PRKCA,RAB7B,CCL24,C1S,CSF1,ADAM10,DCSTAMP,BAIAP2,HLAF,TBX21,BTN3A1,AQP3,SPN,HMOX1,MAPK1,LPXN,CD40LG,UBE2D2,CD38,RHOH,CD1C,TMEM173,CLEC10A,PIK3C3,ITPKB,RIPK2,N4BP2L2,NLRC5,CD84,IL1RL1,CA2,XCL1,TNFSF4,BTN3A3,IL2,CTLA4,XCL2 |
| GO:MF | receptor ligand activity | | 1.72E-04 | IL24,IL19,SEMA6B,IGF1,OSGIN1,KITLG,VEGFD,RETN,CLEC11A,IFNG,SAA2,FLRT2,HAMP,CCL24,CXCL9,SEMA4D,SEMA4C,TGFA,MACC1,CSF1,HGF,PPBP,NDP,CD40LG,BMP1,HBEGF,IL16,FBN1,XCL1,TNFSF4,IL2,XCL2,CXCL11 |
| GO:MF | signaling receptor activator activity | | 1.72E-04 | IL24,IL19,SEMA6B,IGF1,OSGIN1,KITLG,VEGFD,RETN,CLEC11A,IFNG,SAA2,FLRT2,HAMP,CCL24,CXCL9,SEMA4D,SEMA4C,TGFA,MACC1,CSF1,HGF,PPBP,NDP,CD40LG,BMP1,HBEGF,IL16,FBN1,XCL1,TNFSF4,IL2,XCL2,CXCL11 |
| GO:MF | receptor regulator activity | | 2.62E-04 | IL24,WFIKKN2,IL19,SEMA6B,IGF1,OSGIN1,KITLG,VEGFD,RETN,CLEC11A,IFNG,SAA2,FLRT2,HAMP,CCL24,CXCL9,SEMA4D,SEMA4C,TGFA,MACC1,CSF1,HGF,PPBP,NDP,CD40LG,BMP1,HBEGF,IL16,FBN1,XCL1,TNFSF4,IL2,XCL2,CXCL11 |
| GO:MF | oxidoreductase activity | | 5.33E-03 | CYP1A1,CYP1B1,NQO1,CYGB,VCAM1,ALDH3A1,MGST1,PGD,ABCC4,G6PD,GSR,SRXN1,PIR,ACOX2,GPX2,ALOX15B,GPD1,ALDH4A1,MTHFD2L,CYP27B1,SRD5A3,COX15,ALDH5A1,HSD17B4,HMOX1,ALOX5,P3H2,DPYD,CYBB,DHRS7,PAM,MSRB2,HMGCR,AKR1C4,ERO1B,DHRS3,VKORC1L1,TXNRD1,GFOD1 |
| GO:MF | signaling receptor binding | | 5.33E-03 | DTX1,IL24,VCAM1,LAMB1,WFIKKN2,CYTL1,IL19,CD226,SEMA6B,IGF1,OSGIN1,EPHB2,KITLG,VEGFD,SOCS1,RETN,CLEC11A,IFNG,SAA2,FLRT2,SMURF2,HAMP,PRKCA,CCL24,LDLRAP1,CXCL9,SEMA4D,SEMA4C,TGFA,MACC1,CSF1,PIK3CG,ADAM10,S100B,HGF,ANG,BTN3A1,CD72,MTSS1,ANGPTL1,PPBP,NDP,CD40LG,BMP1,HBEGF,IL16,FBN1,SNED1,JAKMIP1,MDM2,PDPN,ENG,TAP1,MADD,PAGR1,RIPK2,RARG,ACE,XCL1,TNFSF4,DDX54,SMAD6,BTN3A3,NRIP1,CCDC88C,IL2,XCL2,CXCL11 |
| GO:MF | cytokine activity | | 6.87E-03 | IL24,IL19,KITLG,IFNG,CCL24,CXCL9,CSF1,PPBP,NDP,CD40LG,BMP1,IL16,XCL1,TNFSF4,IL2,XCL2,CXCL11 |
| GO:MF | endogenous lipid antigen binding | | 2.09E-02 | CD1B,CD1A,CD1C |
| GO:MF | exogenous lipid antigen binding | | 2.09E-02 | CD1B,CD1A,CD1C |
| GO:MF | growth factor activity | | 2.97E-02 | IGF1,OSGIN1,KITLG,VEGFD,CLEC11A,TGFA,MACC1,CSF1,HGF,PPBP,BMP1,HBEGF,IL2 |
| GO:MF | identical protein binding | | 3.22E-02 | NQO1,MGST1,G6PD,ITLN1,EPHB2,OLIG2,ACOX2,VEGFD,GPD1,SMURF2,CARD9,ZBTB7B,TIAF1,ALDH4A1,SDC3,C1S,QPRT,CSF1,FZD4,PIK3CG,ADAM10,S100B,HGF,BAIAP2,ANG,EDC3,MTSS1,TKT,HSD17B4,HMOX1,MAPK1,NDP,BMP1,CD38,FBN1,TPM4,MDM2,DPYD,TMEM173,KEAP1,SRGAP2,ENG,PECAM1,IFI27,TAP1,ACOT7,PAM,ACSL6,IRF5,RIPK2,HMGCR,LACTB,OFD1,CD84,CALCOCO2,XCL1,HARS,PCYT1A,TLE1,SMAD6,LRRFIP1,RAD18,XRCC4,FBP1,NPR3,MAD1L1,H2BC10 |
| GO:MF | lipid antigen binding | | 3.22E-02 | CD1B,CD1A,CD1C |
| GO:MF | molecular function regulator | | 3.47E-02 | RASAL1,IL24,WFIKKN2,GCLM,IL19,MYOZ1,SEMA6B,RCBTB2,IGF1,CST6,OSGIN1,KITLG,CDC37,PKIB,VEGFD,SOCS1,RETN,CACNB3,SH3PXD2A,CLEC11A,STYXL1,IFNG,SAA2,FLRT2,HAMP,DOCK3,IPO7,SERPINB2,CCL24,NRP1,CXCL9,SEMA4D,SEMA4C,TGFA,MACC1,CSF1,PPP1R16B,HGF,RGS12,PPP1R14C,ARHGAP6,ARHGEF19,PPBP,NDP,CD40LG,BMP1,DNAJB6,HBEGF,IL16,RHOH,FBN1,RIC1,RPTOR,ARHGEF28,SRGAP2,RCAN2,MADD,ARHGAP24,ELFN1,ASAP1,RGS16,XCL1,TNFSF4,CCDC88C,IL2,ERCC6,XCL2,CXCL11,FAM20A |
| GO:MF | cytokine receptor binding | | 3.47E-02 | KITLG,VEGFD,IFNG,SMURF2,CCL24,CXCL9,CSF1,PPBP,CD40LG,ENG,MADD,XCL1,TNFSF4,SMAD6,IL2,XCL2,CXCL11 |
| GO:MF | cofactor binding | | 4.42E-02 | CYP1A1,CYP1B1,CYGB,SLC48A1,MGST1,PGD,G6PD,GSR,ACOX2,GPD1,CYP27B1,ACOT11,COX15,TKT,CISD3,HMOX1,P3H2,DPYD,CYBB,GCLC,ACOT7,PAM,HMGCR,TCN2,VKORC1L1,TXNRD1 |
| GO:MF | monosaccharide binding | | 4.42E-02 | G6PD,COLEC12,TALDO1,CD207,P3H2,ENG,PAM,FBP1 |
| GO:MF | glutamate-cysteine ligase activity | | 4.42E-02 | GCLM,GCLC |
| GO:CC | vesicle | | 4.23E-06 | KRT79,SSPN,CD101,VCAM1,SLC48A1,LAMB1,FLT1,FUCA1,ABCB1,RCBTB2,MGST1,PGD,IGF1,ABCC4,G6PD,RHOQ,COLEC12,AK1,ITLN1,GSR,CST6,CLCN4,SLC9A1,LY6K,DNAJC6,CDC37,VEGFD,CD1B,SOCS1,RETN,TALDO1,ALOX15B,TNPO1,CD1A,SEPTIN6,MVB12B,CDK5RAP2,ABCB4,SAA2,GPD1,FLRT2,CD47,GLO1,ATP6V0D2,STAMBPL1,PRKCA,RAB7B,PRLR,FGFRL1,LDLRAP1,NRP1,QPRT,SEMA4C,EVL,TGFA,ACOT11,FAM151A,FZD4,HIST2H2AA3,TMEM163,ADAM10,RIPOR1,AP1AR,DCSTAMP,MON1B,SMPDL3A,HGF,BAIAP2,F13A1,METTL7A,ANG,CD207,HLA-F,KCNQ1,MTSS1,ANGPTL1,GIMAP5,TSPAN14,TKT,SPN,GLIPR1,RHOB,MAPK1,PPBP,KIF13A,ALOX5,BMP1,FAM20C,HBEGF,UBE2D2,CD38,GSN,RPL35A,CTTNBP2,IL15RA,TPM4,AQP7,USE1,GGNBP2,MDM2,CD1C,BPIFA2,TMEM173,HIST1H2BD,MPI,PDPN,CYBB,SNTB2,SRGAP2,HIST2H2BE,MCEMP1,DTNBP1,PECAM1,PIK3C3,SLC1A5,SMPD1,GHITM,TAP1,ACOT7,PAM,ENTPD7,RIPK2,AKR1C4,MYO15A,SLC37A2,N4BP2L2,PIK3C2A,ACE,CALCOCO2,CA2,BICD2,GATM,TCN2,LDLRAD4,CLEC5A,CPM,ANXA11,TXNRD1,CTLA4,WASHC2C,FBP1,PSAT1,NPR3,FAM20A,H2AC8,H2BC10 |
| GO:CC | cytoplasm | | 6.11E-05 | LRP5,CYP1A1,PDE3B,RASAL1,KRT79,AHRR,NPAS2,SSPN,AK8,CYP1B1,NQO1,DTX1,CYGB,VCAM1,SLC48A1,LAMB1,ECSCR,COL23A1,GCLM,ALDH3A1,FLT1,XYLT1,FUCA1,MYOZ1,RCBTB2,MGST1,ADK,PGD,IGF1,FUT1,STAC,ABCC4,G6PD,RHOQ,COLEC12,AK1,GSR,APMAP,SRXN1,EPHB2,OLIG2,PTGFRN,UBD,CLCN4,KITLG,PIR,NUB1,GPAT3,SLC9A1,LY6K,ACOX2,DNAJC6,CDC37,PKIB,VEGFD,MEF2A,ATOH8,DTNA,CD1B,SOCS1,GPX2,RETN,TALDO1,CACNB3,SH3PXD2A,ALOX15B,TNPO1,CD1A,SEPTIN6,KMT5A,MVB12B,CLEC11A,STYXL1,SPRY1,MARCH9,SLC7A11,PPA2,LACC1,CDK5RAP2,ABCB4,GPD1,FLRT2,CD47,SMURF2,HAMP,DDIT4L,GLO1,CARD9,DOCK3,ALDH4A1,ATP6V0D2,GADD45GIP1,KAZN,STAMBPL1,NSUN3,MTHFD2L,PRKCA,IPO7,RAB7B,SERPINB2,PRLR,TMEM97,ELOVL6,SDC3,NNMT,TGM5,FGFRL1,RIMKLB,MCRIP2,NUP210,FBXO15,LDLRAP1,CYP27B1,NRP1,DTX4,PRCD,SRD5A3,ARL4A,QPRT,SEMA4C,EVL,TGFA,ACOT11,COX15,MACC1,EIF3J,CSF1,EVC,SEPTIN9,FZD4,PIK3CG,SSH1,TMEM163,ADAM10,DCANP1,PELP1,RIPOR1,RMDN2,S100B,AP1AR,DCSTAMP,KLHDC8B,CMIP,MON1B,PID1,NDUFAF4,PPP1R16B,HGF,MAP3K20,RGS12,BAIAP2,F13A1,CDKN3,ZNF746,METTL7A,TESMIN,ANG,CD207,SFXN5,HLAF,DCAF7,KCNQ1,EDC3,GALNT12,AQP3,MTSS1,ALDH5A1,PPP1R14C,GIMAP5,TSPAN14,TKT,GLIPR1,SEC62,RHEBL1,CISD3,HSD17B4,ASRGL1,NLN,ARHGAP6,HMOX1,RHOB,ARHGEF19,MAPK1,DDIAS,PPBP,SSTR2,KIF13A,EPB41L3,ALOX5,LPXN,NAV1,BMP1,FAM20C,DNAJB6,FAIM2,HBEGF,PLCB3,IL16,UBE2D2,CD38,GSN,RPL35A,CTTNBP2,RHOH,FBN1,IL15RA,TPM4,MKKS,AQP7,JAKMIP1,BORCS8,RBM4,P3H2,USE1,GGNBP2,FBXO30,MDM2,CD1C,DPYD,TMEM173,RIC1,SYNPO,CARD14,RPTOR,HIST1H2BD,KEAP1,MPI,PDPN,ZNF365,ARHGEF28,CYBB,SNTB2,SRGAP2,HIST2H2BE,GCLC,MCEMP1,DTNBP1,PECAM1,PIK3C3,SLC1A5,RCAN2,SMPD1,GHITM,IFI27,IFT27,GZMH,ITPKB,STRBP,TAP1,ACOT7,CDC14A,PAM,TRIM16,MADD,METAP1D,ENTPD7,MPHOSPH6,MSRB2,RPTN,ELOB,APOL3,ARHGAP24,ST6GALNAC6,ELK3,ACSL6,IRF5,SYNE3,RIPK2,HMGCR,AKR1C4,LACTB,MYO15A,RHBDD2,OFD1,SLC37A2,RARG,NLRC5,PIK3C2A,ACE,CALCOCO2,ASAP1,RGS16,IL1RL1,CA2,BICD2,GATM,ATP8B2,TCN2,DDX54,INSIG1,HARS,EFHC1,ERO1B,RPP14,SLC23A2,HRH1,VKORC1L1,B3GALNT1,CEP41,LDLRAD4,CLEC5A,OMA1,PCYT1A,NEURL2,CDS2,TLE1,SMAD6,LRRFIP1,MSI2,SFXN2,RAD18,NRIP1,CARS2,ANXA11,CCDC88C,TXNRD1,XRCC4,CTLA4,NAA15,WASHC2C,JAKMIP2,FBP1,PCLAF,PSAT1,ERMP1,SPECC1,TAF1D,DMXL1,NAP1L4,FAM20A,MAD1L1,H2BC10 |
| GO:CC | extracellular space | | 6.88E-05 | KRT79,CD101,IL24,VCAM1,LAMB1,WFIKKN2,COL23A1,CYTL1,IL19,ALDH3A1,FLT1,XYLT1,FUCA1,ABCB1,SEMA6B,PGD,IGF1,G6PD,RHOQ,COLEC12,AK1,ITLN1,GSR,CST6,KITLG,SLC9A1,CDC37,VEGFD,CD1B,RETN,TALDO1,ALOX15B,TNPO1,CD1A,LRRN3,MVB12B,CLEC11A,CDK5RAP2,IFNG,ABCB4,SAA2,GPD1,FLRT2,CD47,HAMP,GLO1,ATP6V0D2,PRKCA,SERPINB2,CCL24,C1S,NRP1,CXCL9,SEMA4D,HAPLN3,QPRT,SEMA4C,TGFA,ACOT11,CSF1,FAM151A,HIST2H2AA3,ADAM10,RIPOR1,S100B,SMPDL3A,HGF,BAIAP2,F13A1,ANG,HLA-F,ANGPTL1,TKT,SPN,GLIPR1,HMOX1,RHOB,PPBP,NDP,ALOX5,CD40LG,BMP1,FAM20C,HBEGF,IL16,UBE2D2,CD38,GSN,RPL35A,FBN1,IL15RA,TPM4,CD1C,BPIFA2,HIST1H2BD,MPI,ENG,HIST2H2BE,PECAM1,SLC1A5,SMPD1,GHITM,ACOT7,PAM,ELFN1,AKR1C4,MYO15A,SLC37A2,N4BP2L2,PIK3C2A,ACE,CA2,XCL1,GATM,TCN2,TNFSF4,CPM,PLTP,ANXA11,IL2,TXNRD1,XCL2,FBP1,PSAT1,NPR3,CXCL11,FAM20A,H2AC8,H2BC10 |
| GO:CC | cell surface | | 1.51E-04 | VCAM1,CD226,CXCR5,ABCB1,ABCC4,GSR,APMAP,PTGFRN,SLC9A1,CD1B,SLC1A2,CD1A,CHRNA1,SLC7A11,CD47,PRLR,SDC3,NRP1,CXCL9,TGFA,SLC1A3,FZD4,ADAM10,DCSTAMP,HLA-F,BTN3A1,FCER1A,TSPAN14,SPN,NDP,CD40LG,HBEGF,CD38,IL15RA,CD1C,ENG,PECAM1,TSPAN32,PAM,ACE,IL1RL1,TNFSF4,CSF2RA,CLEC5A,BTN3A3,CPM,CTLA4 |
| GO:CC | extracellular region | | 4.64E-04 | KRT79,CD101,IL24,VCAM1,LAMB1,WFIKKN2,COL23A1,CYTL1,IL19,ALDH3A1,FLT1,XYLT1,FUCA1,ABCB1,SEMA6B,PGD,IGF1,G6PD,RHOQ,COLEC12,PLA2G2D,AK1,ITLN1,GSR,CST6,EPHB2,KITLG,SLC9A1,LY6K,CDC37,VEGFD,CD1B,FCMR,RETN,TALDO1,ALOX15B,TNPO1,CD1A,LRRN3,MVB12B,CLEC11A,CDK5RAP2,IFNG,ABCB4,SAA2,GPD1,FLRT2,CD47,HAMP,GLO1,ATP6V0D2,PRKCA,SERPINB2,PRLR,CCL24,SDC3,OLFML2B,C1S,NRP1,PRCD,CXCL9,SEMA4D,HAPLN3,QPRT,SEMA4C,TGFA,ACOT11,CSF1,FAM151A,HIST2H2AA3,ADAM10,RIPOR1,S100B,KLHDC8B,SMPDL3A,HGF,BAIAP2,F13A1,METTL7A,ANG,HLA-F,ANGPTL1,TKT,SPN,GLIPR1,NLN,HMOX1,RHOB,MAPK1,PPBP,NDP,ALOX5,CD40LG,BMP1,FAM20C,HBEGF,IL16,UBE2D2,CD38,GSN,RPL35A,FBN1,IL15RA,TPM4,SNED1,PRADC1,P3H2,CD1C,BPIFA2,RNASE4,HIST1H2BD,MPI,ENG,HIST2H2BE,PECAM1,SLC1A5,SMPD1,GHITM,ACOT7,PAM,RPTN,APOL3,ADGRF1,ELFN1,AKR1C4,MYO15A,OFD1,SLC37A2,N4BP2L2,PIK3C2A,ACE,IL1RL1,CA2,XCL1,GATM,TCN2,TNFSF4,CSF2RA,CPM,PLTP,ANXA11,IL2,TXNRD1,XCL2,GFOD1,FBP1,PSAT1,NPR3,CXCL11,FAM20A,H2AC8,H2BC10 |
| GO:CC | cytoplasmic vesicle | | 1.16E-03 | SSPN,VCAM1,SLC48A1,FLT1,FUCA1,RCBTB2,MGST1,IGF1,ABCC4,RHOQ,COLEC12,CLCN4,LY6K,VEGFD,CD1B,SOCS1,RETN,CD1A,SEPTIN6,MVB12B,ABCB4,CD47,ATP6V0D2,STAMBPL1,RAB7B,PRLR,FGFRL1,LDLRAP1,NRP1,SEMA4C,EVL,TGFA,FZD4,TMEM163,ADAM10,AP1AR,DCSTAMP,MON1B,HGF,BAIAP2,F13A1,METTL7A,ANG,CD207,HLA-F,KCNQ1,MTSS1,GIMAP5,TSPAN14,GLIPR1,RHOB,MAPK1,PPBP,KIF13A,ALOX5,HBEGF,CD38,GSN,CTTNBP2,IL15RA,AQP7,USE1,GGNBP2,MDM2,CD1C,TMEM173,PDPN,CYBB,SNTB2,SRGAP2,MCEMP1,DTNBP1,PECAM1,PIK3C3,SLC1A5,SMPD1,TAP1,PAM,ENTPD7,PIK3C2A,ACE,CALCOCO2,BICD2,TCN2,LDLRAD4,CLEC5A,ANXA11,CTLA4,WASHC2C |
| GO:CC | intracellular vesicle | | 1.16E-03 | SSPN,VCAM1,SLC48A1,FLT1,FUCA1,RCBTB2,MGST1,IGF1,ABCC4,RHOQ,COLEC12,CLCN4,LY6K,VEGFD,CD1B,SOCS1,RETN,CD1A,SEPTIN6,MVB12B,ABCB4,CD47,ATP6V0D2,STAMBPL1,RAB7B,PRLR,FGFRL1,LDLRAP1,NRP1,SEMA4C,EVL,TGFA,FZD4,TMEM163,ADAM10,AP1AR,DCSTAMP,MON1B,HGF,BAIAP2,F13A1,METTL7A,ANG,CD207,HLA-F,KCNQ1,MTSS1,GIMAP5,TSPAN14,GLIPR1,RHOB,MAPK1,PPBP,KIF13A,ALOX5,HBEGF,CD38,GSN,CTTNBP2,IL15RA,AQP7,USE1,GGNBP2,MDM2,CD1C,TMEM173,PDPN,CYBB,SNTB2,SRGAP2,MCEMP1,DTNBP1,PECAM1,PIK3C3,SLC1A5,SMPD1,TAP1,PAM,ENTPD7,PIK3C2A,ACE,CALCOCO2,BICD2,TCN2,LDLRAD4,CLEC5A,ANXA11,CTLA4,WASHC2C |
| GO:CC | whole membrane | | 2.86E-03 | SLC48A1,CD226,MGST1,ABCC4,RHOQ,COLEC12,ITLN1,CLCN4,SLC9A1,LY6K,CD1B,PAG1,CD1A,MVB12B,MARCH9,ABCB4,CD47,SMURF2,ATP6V0D2,RAB7B,LDLRAP1,CYP27B1,SEMA4C,TGFA,FZD4,TMEM163,ADAM10,DCSTAMP,CD207,HLAF,KCNQ1,GIMAP5,TSPAN14,UNC5B,GLIPR1,HSD17B4,HMOX1,RHOB,MAPK1,KIF13A,FAIM2,HBEGF,CD38,BORCS8,MDM2,CD1C,TMEM173,RPTOR,PDPN,CYBB,SNTB2,MCEMP1,DTNBP1,PECAM1,PIK3C3,IFI27,TAP1,PAM,ENTPD7,ACSL6,SYNE3,HMGCR,CALCOCO2,BICD2,LDLRAD4,CLEC5A,WASHC2C |
| GO:CC | cell periphery | | 1.63E-02 | LRP5,GPR68,RASAL1,SSPN,CD101,VCAM1,SLC48A1,ECSCR,COL23A1,ALDH3A1,CD226,CXCR5,FLT1,ABCB1,DAGLA,SEMA6B,MGST1,P2RY6,IGF1,FUT1,STAC,ABCC4,G6PD,RHOQ,COLEC12,AK1,ITLN1,GSR,CST6,EPHB2,CLCN4,KITLG,SLC9A1,LY6K,ADORA2B,DTNA,CD1B,SLC1A2,CACNB3,PAG1,ALOX15B,CD1A,SEPTIN6,CHRNA1,MVB12B,SPRY1,SLC7A11,ABCB4,FLRT2,CD47,SMURF2,HAMP,GLO1,CARD9,PIK3IP1,ATP6V0D2,KAZN,PRKCA,SERPINB2,PRLR,TMEM97,SDC3,TGM5,FGFRL1,SLC4A8,LDLRAP1,NRP1,PRCD,CXCL9,ARL4A,SEMA4D,SEMA4C,TGFA,NKG7,SLC1A3,CSF1,EVC,SEPTIN9,FZD4,PIK3CG,SSH1,ADAM10,DCSTAMP,KLHDC8B,PPP1R16B,RGS12,BAIAP2,CD207,HLAF,BTN3A1,KCNQ1,CD72,AQP3,FCER1A,TSPAN14,UNC5B,SPN,GLIPR1,RHEBL1,NLN,HMOX1,RHOB,MAPK1,SSTR2,OR2A7,EPB41L3,LPXN,CD40LG,FAIM2,HBEGF,IL16,CD38,GSN,CTTNBP2,RHOH,LPAR3,LPAR5,IL15RA,MPP7,AQP7,USE1,MDM2,CD1C,TMEM173,CARD14,PDPN,ARHGEF28,CYBB,SNTB2,SRGAP2,ENG,CLEC10A,MCEMP1,DTNBP1,PECAM1,TSPAN32,SLC1A5,SMPD1,GPR35,PAM,TRIM16,MADD,RPTN,ADCY9,ST6GALNAC6,ADGRF1,ACSL6,CHRM3,CLEC1A,PIK3C2A,ACE,CD84,ASAP1,RGS16,IL1RL1,CA2,BICD2,ATP8B2,TNFSF4,CSF2RA,DHRS3,SLC23A2,SLC39A8,HRH1,CLEC5A,PCYT1A,BTN3A3,CPM,LRRFIP1,CALHM6,CTLA4,WASHC2C,NPR3 |
| GO:CC | plasma membrane | | 1.63E-02 | LRP5,GPR68,RASAL1,SSPN,CD101,VCAM1,SLC48A1,ECSCR,COL23A1,ALDH3A1,CD226,CXCR5,FLT1,ABCB1,DAGLA,SEMA6B,MGST1,P2RY6,IGF1,FUT1,STAC,ABCC4,G6PD,RHOQ,COLEC12,AK1,ITLN1,GSR,CST6,EPHB2,CLCN4,KITLG,SLC9A1,LY6K,ADORA2B,DTNA,CD1B,SLC1A2,CACNB3,PAG1,ALOX15B,CD1A,SEPTIN6,CHRNA1,MVB12B,SPRY1,SLC7A11,ABCB4,FLRT2,CD47,SMURF2,GLO1,CARD9,PIK3IP1,ATP6V0D2,KAZN,PRKCA,SERPINB2,PRLR,TMEM97,SDC3,TGM5,FGFRL1,SLC4A8,LDLRAP1,NRP1,PRCD,CXCL9,ARL4A,SEMA4D,SEMA4C,TGFA,NKG7,SLC1A3,CSF1,EVC,FZD4,PIK3CG,SSH1,ADAM10,DCSTAMP,KLHDC8B,PPP1R16B,RGS12,BAIAP2,CD207,HLAF,BTN3A1,KCNQ1,CD72,AQP3,FCER1A,TSPAN14,UNC5B,SPN,GLIPR1,RHEBL1,NLN,HMOX1,RHOB,MAPK1,SSTR2,OR2A7,EPB41L3,LPXN,CD40LG,FAIM2,HBEGF,IL16,CD38,GSN,RHOH,LPAR3,LPAR5,IL15RA,MPP7,AQP7,USE1,MDM2,CD1C,TMEM173,CARD14,PDPN,ARHGEF28,CYBB,SNTB2,SRGAP2,ENG,CLEC10A,MCEMP1,DTNBP1,PECAM1,TSPAN32,SLC1A5,SMPD1,GPR35,PAM,TRIM16,MADD,RPTN,ADCY9,ST6GALNAC6,ADGRF1,ACSL6,CHRM3,CLEC1A,PIK3C2A,ACE,CD84,ASAP1,RGS16,IL1RL1,CA2,BICD2,ATP8B2,TNFSF4,CSF2RA,DHRS3,SLC23A2,SLC39A8,HRH1,CLEC5A,PCYT1A,BTN3A3,CPM,LRRFIP1,CALHM6,CTLA4,WASHC2C,NPR3 |
| GO:CC | endocytic vesicle | | 1.99E-02 | COLEC12,ATP6V0D2,RAB7B,EVL,FZD4,CD207,HLAF,MTSS1,HBEGF,GSN,MDM2,CYBB,SRGAP2,PIK3C3,TAP1,ENTPD7,ANXA11,CTLA4 |
| GO:CC | cytosol | | 2.06E-02 | PDE3B,RASAL1,KRT79,AHRR,NPAS2,AK8,NQO1,DTX1,CYGB,ECSCR,GCLM,ALDH3A1,ADK,PGD,STAC,G6PD,RHOQ,AK1,GSR,SRXN1,EPHB2,UBD,PIR,NUB1,ACOX2,DNAJC6,CDC37,MEF2A,SOCS1,GPX2,TALDO1,CACNB3,SH3PXD2A,ALOX15B,TNPO1,KMT5A,MVB12B,SPRY1,CDK5RAP2,ABCB4,GPD1,SMURF2,GLO1,CARD9,DOCK3,KAZN,STAMBPL1,PRKCA,IPO7,NNMT,RIMKLB,FBXO15,LDLRAP1,NRP1,DTX4,ARL4A,QPRT,EVL,ACOT11,EIF3J,PIK3CG,SSH1,RMDN2,S100B,AP1AR,KLHDC8B,CMIP,MAP3K20,RGS12,BAIAP2,CDKN3,DCAF7,EDC3,TKT,SEC62,HSD17B4,ASRGL1,ARHGAP6,HMOX1,RHOB,ARHGEF19,MAPK1,SSTR2,EPB41L3,ALOX5,LPXN,DNAJB6,PLCB3,IL16,UBE2D2,GSN,RPL35A,RHOH,TPM4,MKKS,BORCS8,RBM4,FBXO30,MDM2,DPYD,TMEM173,RIC1,SYNPO,RPTOR,HIST1H2BD,KEAP1,MPI,PDPN,ARHGEF28,SRGAP2,HIST2H2BE,GCLC,DTNBP1,PIK3C3,ITPKB,ACOT7,CDC14A,TRIM16,MADD,MPHOSPH6,MSRB2,RPTN,ELOB,ARHGAP24,IRF5,RIPK2,AKR1C4,LACTB,OFD1,NLRC5,PIK3C2A,CALCOCO2,ASAP1,IL1RL1,CA2,BICD2,HARS,HRH1,CEP41,CLEC5A,PCYT1A,NEURL2,TLE1,SMAD6,LRRFIP1,NRIP1,ANXA11,TXNRD1,XRCC4,NAA15,WASHC2C,FBP1,PSAT1,SPECC1,TAF1D,MAD1L1,H2BC10 |
| GO:CC | endocytic vesicle membrane | | 2.31E-02 | COLEC12,ATP6V0D2,RAB7B,FZD4,CD207,HLA-F,HBEGF,MDM2,CYBB,PIK3C3,TAP1,ENTPD7 |
| GO:CC | podosome | | 2.41E-02 | VCAM1,SH3PXD2A,LPXN,GSN,ASAP1 |
| GO:CC | glutamate-cysteine ligase complex | | 2.41E-02 | GCLM,GCLC |
| GO:CC | extracellular exosome | | 3.42E-02 | KRT79,CD101,VCAM1,LAMB1,FUCA1,ABCB1,PGD,G6PD,RHOQ,AK1,ITLN1,GSR,CST6,SLC9A1,CDC37,RETN,TALDO1,ALOX15B,TNPO1,MVB12B,CDK5RAP2,ABCB4,SAA2,GPD1,FLRT2,CD47,GLO1,ATP6V0D2,PRKCA,QPRT,ACOT11,FAM151A,HIST2H2AA3,ADAM10,RIPOR1,SMPDL3A,BAIAP2,ANGPTL1,TKT,SPN,RHOB,FAM20C,UBE2D2,CD38,GSN,RPL35A,TPM4,BPIFA2,HIST1H2BD,MPI,HIST2H2BE,PECAM1,SLC1A5,SMPD1,GHITM,ACOT7,PAM,AKR1C4,MYO15A,SLC37A2,N4BP2L2,PIK3C2A,ACE,CA2,GATM,CPM,ANXA11,TXNRD1,FBP1,PSAT1,NPR3,FAM20A,H2AC8,H2BC10 |
| GO:CC | external side of plasma membrane | | 3.42E-02 | VCAM1,CD226,CXCR5,ABCB1,ABCC4,GSR,CD1B,CD1A,PRLR,CXCL9,HLA-F,BTN3A1,CD40LG,CD1C,ENG,PECAM1,ACE,IL1RL1,CSF2RA,BTN3A3,CTLA4 |
| GO:CC | vesicle membrane | | 3.64E-02 | MGST1,ABCC4,COLEC12,CD47,ATP6V0D2,RAB7B,LDLRAP1,SEMA4C,TGFA,FZD4,TMEM163,ADAM10,RIPOR1,CD207,HLAF,KCNQ1,TSPAN14,GLIPR1,HBEGF,CD38,IL15RA,AQP7,MDM2,TMEM173,CYBB,SNTB2,MCEMP1,DTNBP1,PECAM1,PIK3C3,TAP1,PAM,ENTPD7,CLEC5A |
| GO:CC | phagocytic vesicle | | 3.84E-02 | ATP6V0D2,RAB7B,EVL,HLA-F,GSN,CYBB,SRGAP2,PIK3C3,TAP1,ANXA11 |
| GO:CC | extracellular vesicle | | 3.84E-02 | KRT79,CD101,VCAM1,LAMB1,FUCA1,ABCB1,PGD,G6PD,RHOQ,AK1,ITLN1,GSR,CST6,SLC9A1,CDC37,RETN,TALDO1,ALOX15B,TNPO1,MVB12B,CDK5RAP2,ABCB4,SAA2,GPD1,FLRT2,CD47,GLO1,ATP6V0D2,PRKCA,QPRT,ACOT11,FAM151A,HIST2H2AA3,ADAM10,RIPOR1,SMPDL3A,BAIAP2,ANGPTL1,TKT,SPN,RHOB,FAM20C,UBE2D2,CD38,GSN,RPL35A,TPM4,BPIFA2,HIST1H2BD,MPI,HIST2H2BE,PECAM1,SLC1A5,SMPD1,GHITM,ACOT7,PAM,AKR1C4,MYO15A,SLC37A2,N4BP2L2,PIK3C2A,ACE,CA2,GATM,CPM,ANXA11,TXNRD1,FBP1,PSAT1,NPR3,FAM20A,H2AC8,H2BC10 |
| GO:CC | extracellular organelle | | 3.84E-02 | KRT79,CD101,VCAM1,LAMB1,FUCA1,ABCB1,PGD,G6PD,RHOQ,AK1,ITLN1,GSR,CST6,SLC9A1,CDC37,RETN,TALDO1,ALOX15B,TNPO1,MVB12B,CDK5RAP2,ABCB4,SAA2,GPD1,FLRT2,CD47,GLO1,ATP6V0D2,PRKCA,QPRT,ACOT11,FAM151A,HIST2H2AA3,ADAM10,RIPOR1,SMPDL3A,BAIAP2,ANGPTL1,TKT,SPN,RHOB,FAM20C,UBE2D2,CD38,GSN,RPL35A,TPM4,BPIFA2,HIST1H2BD,MPI,HIST2H2BE,PECAM1,SLC1A5,SMPD1,GHITM,ACOT7,PAM,AKR1C4,MYO15A,SLC37A2,N4BP2L2,PIK3C2A,ACE,CA2,GATM,CPM,ANXA11,TXNRD1,FBP1,PSAT1,NPR3,FAM20A,H2AC8,H2BC10 |
| GO:CC | cytoplasmic vesicle membrane | | 3.84E-02 | MGST1,ABCC4,COLEC12,CD47,ATP6V0D2,RAB7B,LDLRAP1,SEMA4C,TGFA,FZD4,TMEM163,ADAM10,CD207,HLA-F,KCNQ1,TSPAN14,GLIPR1,HBEGF,CD38,IL15RA,AQP7,MDM2,TMEM173,CYBB,SNTB2,MCEMP1,DTNBP1,PECAM1,PIK3C3,TAP1,PAM,ENTPD7,CLEC5A |
| GO:CC | tetraspanin-enriched microdomain | | 3.93E-02 | ADAM10,TSPAN14,PDPN |
| GO:CC | kinociliary basal body | | 4.21E-02 | MKKS,CDC14A |
| GO:CC | endomembrane system | | 4.21E-02 | LRP5,CYP1A1,PDE3B,SSPN,CYP1B1,VCAM1,SLC48A1,LAMB1,COL23A1,ALDH3A1,FLT1,XYLT1,FUCA1,RCBTB2,MGST1,IGF1,FUT1,ABCC4,APMAP,PTGFRN,CLCN4,GPAT3,SLC9A1,LY6K,VEGFD,CD1B,RETN,CD1A,SEPTIN6,MVB12B,SPRY1,MARCH9,SLC7A11,CDK5RAP2,FLRT2,CD47,ATP6V0D2,STAMBPL1,PRKCA,IPO7,RAB7B,PRLR,TMEM97,ELOVL6,SDC3,FGFRL1,NUP210,LDLRAP1,NRP1,PRCD,SRD5A3,SEMA4C,TGFA,CSF1,TMEM163,ADAM10,RIPOR1,RMDN2,AP1AR,DCSTAMP,MON1B,HGF,BAIAP2,F13A1,METTL7A,CD207,HLAF,KCNQ1,GALNT12,GIMAP5,TSPAN14,GLIPR1,SEC62,RHEBL1,HMOX1,RHOB,MAPK1,PPBP,EMC3,KIF13A,ALOX5,BMP1,FAM20C,ABHD4,FAIM2,CD38,GSN,CTTNBP2,FBN1,IL15RA,P3H2,USE1,CD1C,TMEM173,RIC1,SYNPO,KEAP1,CYBB,SNTB2,MCEMP1,DTNBP1,PECAM1,PIK3C3,SMPD1,IFI27,IFT27,TAP1,PAM,ST6GALNAC6,ACSL6,SYNE3,HMGCR,RHBDD2,SLC37A2,PIK3C2A,ACE,BICD2,ATP8B2,TCN2,DDX54,INSIG1,ERO1B,DHRS3,VKORC1L1,B3GALNT1,LDLRAD4,CLEC5A,PCYT1A,CDS2,SMAD6,ANXA11,CTLA4,WASHC2C,JAKMIP2,ERMP1,FAM20A,MAD1L1 |
| KEGG | Ferroptosis | | 1.95E-02 | GCLM,SLC7A11,HMOX1,CYBB,GCLC,ACSL6,SLC39A8 |
| KEGG | Viral protein interaction with cytokine and cytokine receptor | | 1.95E-02 | IL24,IL19,CXCR5,CCL24,CXCL9,CSF1,PPBP,XCL1,IL2,XCL2,CXCL11 |
| WIKI | Phytochemical activity on NRF2 transcriptional activation | | 3.36E-06 | NQO1,GCLM,EPHB2,SLC7A11,PRKCA,HMOX1,KEAP1,GCLC |
| WIKI | Nuclear Receptors Meta-Pathway | | 4.93E-06 | CYP1A1,AHRR,CYP1B1,NQO1,GCLM,ALDH3A1,ABCB1,MGST1,PGD,ABCC4,G6PD,GSR,SRXN1,CDC37,GPX2,SPRY1,SLC7A11,IFNG,ABCB4,SERPINB2,TGFA,HGF,PPP1R14C,HMOX1,HBEGF,KEAP1,GCLC,SLC39A8,PLTP,NRIP1,IL2,TXNRD1 |
| WIKI | NRF2-ARE regulation | | 6.74E-05 | NQO1,GCLM,EPHB2,SLC7A11,PRKCA,HMOX1,KEAP1,GCLC |
| WIKI | NRF2 pathway | | 1.59E-04 | NQO1,GCLM,ALDH3A1,PGD,ABCC4,G6PD,GSR,SRXN1,GPX2,SLC7A11,TGFA,HGF,HMOX1,HBEGF,KEAP1,GCLC,SLC39A8,TXNRD1 |
| WIKI | Aryl Hydrocarbon Receptor Pathway | | 2.37E-04 | CYP1A1,AHRR,CYP1B1,NQO1,ALDH3A1,MGST1,CDC37,IFNG,SERPINB2,IL2 |
| WIKI | Oxidative Stress | | 4.74E-04 | CYP1A1,NQO1,MGST1,GSR,HMOX1,CYBB,GCLC,TXNRD1 |
| WIKI | Photodynamic therapy-induced NFE2L2 (NRF2) survival signaling | | 4.74E-04 | NQO1,GCLM,ABCC4,SRXN1,HMOX1,KEAP1,GCLC |
| WIKI | Pentose Phosphate Metabolism | | 2.10E-03 | PGD,G6PD,TALDO1,TKT |
| WIKI | Aryl Hydrocarbon Receptor Netpath | | 7.09E-03 | CYP1A1,AHRR,CYP1B1,NQO1,CDC37,MAPK1,GCLC,NRIP1 |
| WIKI | Ferroptosis | | 1.38E-02 | GCLM,SLC7A11,HMOX1,CYBB,GCLC,ACSL6,SLC39A8 |
| WIKI | Glutathione metabolism | | 2.06E-02 | GCLM,G6PD,GSR,GPX2,GCLC |
| WIKI | Allograft Rejection | | 2.53E-02 | CXCR5,ABCB1,IFNG,CXCL9,HLA-F,CD40LG,HARS,IL2,CTLA4,CXCL11 |

***FIGURE S2*** *Differential expression analysis in* ***A.*** *whole blood and* ***B.*** *sputum cells between male and female patients (primary analysis; baseline samples). Volcano plot depicting all detected probe sets and coloured by fold change (FC) and adjusted p-value (pFDR): green, FC >|1.3| and pFDR <0.05; red, pFDR<0.05; orange, FC>|1.3 |. Abbreviations: FC, fold-change; pFDR, adjusted p-value*

***
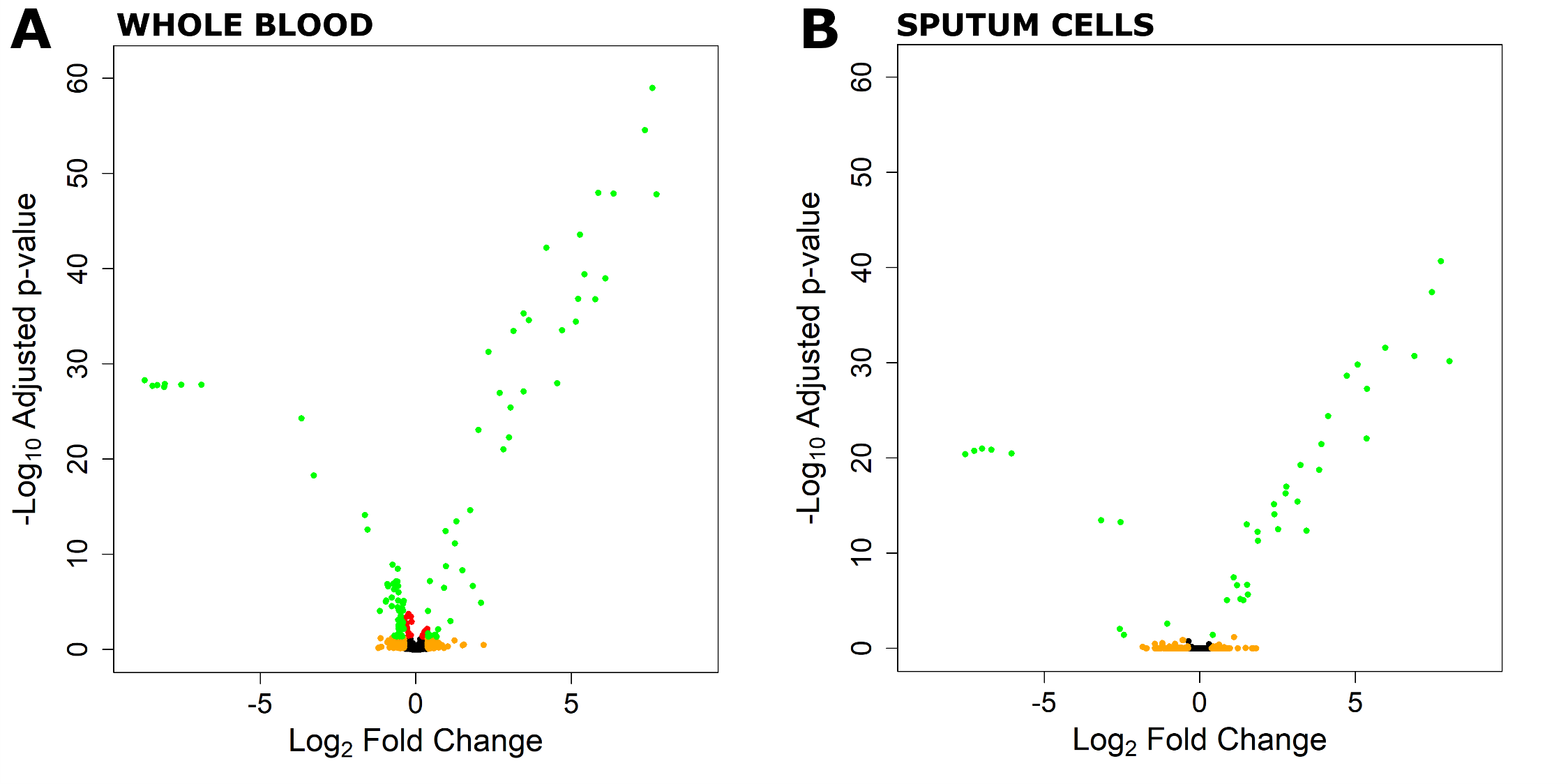
***

***TABLE S4*** *Probe sets significantly (pFDR<0.05) differentially expressed in sputum cells between male and female patients (primary analysis; baseline samples). pFDR, adjusted p-value*

| Probe set ID | Gene Symbol | pFDR value | Fold Change | Gene Title |
| --- | --- | --- | --- | --- |
| 201909_at | RPS4Y1 | 2.06E-41 | 215.16 | ribosomal protein S4 Y-linked 1 |
| 205000_at | DDX3Y | 3.94E-38 | 175.91 | DEAD-box helicase 3 Y-linked |
| 206700_s_at | KDM5D | 2.56E-32 | 62.28 | lysine demethylase 5D |
| 205001_s_at | DDX3Y | 1.88E-31 | 118.53 | DEAD-box helicase 3 Y-linked |
| 204409_s_at | EIF1AY | 6.96E-31 | 260.19 | eukaryotic translation initiation factor 1A Y-linked |
| 228492_at | USP9Y | 1.48E-30 | 33.50 | ubiquitin specific peptidase 9 Y-linked |
| 211149_at | UTY | 2.40E-29 | 26.35 | ubiquitously transcribed tetratricopeptide repeat containing, Y-linked |
| 230760_at | ZFY | 5.21E-28 | 41.19 | zinc finger protein Y-linked |
| 232618_at | TXLNGY | 3.88E-25 | 17.44 | taxilin gamma pseudogene, Y-linked |
| 204410_at | EIF1AY | 8.70E-23 | 41.11 | eukaryotic translation initiation factor 1A Y-linked |
| 223645_s_at | TXLNGY | 3.32E-22 | 14.99 | taxilin gamma pseudogene, Y-linked |
| 224588_at | XIST | 1.00E-21 | -127.95 | X inactive specific transcript |
| 227671_at | XIST | 1.33E-21 | -104.02 | X inactive specific transcript |
| 214218_s_at | XIST | 1.83E-21 | -152.44 | X inactive specific transcript |
| 231592_at | XIST /// TSIX | 3.20E-21 | -66.46 | X inactive specific transcript /// TSIX transcript, XIST antisense RNA |
| 221728_x_at | XIST | 4.04E-21 | -186.40 | X inactive specific transcript |
| 1560800_at | --- | 5.67E-20 | 9.41 | --- |
| 223646_s_at | TXLNGY | 1.73E-19 | 14.30 | taxilin gamma pseudogene, Y-linked |
| 237225_at | --- | 1.06E-17 | 6.85 | --- |
| 214131_at | TXLNGY | 5.36E-17 | 6.72 | taxilin gamma pseudogene, Y-linked |
| 1570360_s_at | DDX3Y | 3.87E-16 | 8.81 | DEAD-box helicase 3 Y-linked |
| 214983_at | TTTY15 | 7.29E-16 | 5.23 | testis-specific transcript, Y-linked 15 |
| 207063_at | TTTY14 | 8.12E-15 | 5.25 | testis-specific transcript, Y-linked 14 |
| 235446_at | XIST | 3.59E-14 | -9.06 | X inactive specific transcript |
| 228919_at | --- | 5.35E-14 | -5.85 | --- |
| 208067_x_at | UTY | 9.73E-14 | 2.85 | ubiquitously transcribed tetratricopeptide repeat containing, Y-linked |
| 236694_at | TXLNGY | 2.96E-13 | 5.73 | taxilin gamma pseudogene, Y-linked |
| 244482_at | --- | 4.19E-13 | 10.79 | --- |
| 210322_x_at | UTY | 5.78E-13 | 3.61 | ubiquitously transcribed tetratricopeptide repeat containing, Y-linked |
| 206624_at | USP9Y | 5.01E-12 | 3.66 | ubiquitin specific peptidase 9 Y-linked |
| 239677_at | --- | 3.60E-08 | 2.13 | --- |
| 1556677_at | --- | 2.07E-07 | 2.86 | --- |
| 207246_at | ZFY | 2.25E-07 | 2.29 | zinc finger protein Y-linked |
| 224293_at | TTTY10 | 2.16E-06 | 2.90 | testis-specific transcript, Y-linked 10 |
| 1560395_at | --- | 6.67E-06 | 2.48 | --- |
| 206279_at | PRKY | 8.18E-06 | 1.83 | protein kinase Y-linked (pseudogene) |
| 1564510_at | --- | 8.22E-06 | 2.63 | --- |
| 203903_s_at | HEPH | 2.60E-03 | -2.07 | hephaestin |
| 1567913_at | --- | 9.00E-03 | -5.94 | --- |
| 1567912_s_at | CT45A3 /// CT45A5 /// CT45A6 /// CT45A1 /// CT45A2 /// CT45A7 /// CT45A10 /// CT45A9 /// CT45A8 | 3.67E-02 | -5.44 | cancer/testis antigen family 45 member A3 /// cancer/testis antigen family 45 member A5 /// cancer/testis antigen family 45 member A6 /// cancer/testis antigen family 45 member A1 /// cancer/testis antigen family 45 member A2 /// cancer/testis antigen family 45 member A7 /// cancer/testis antigen family 45 member A10 /// cancer/testis antigen family 45 member A9 /// cancer/testis antigen family 45 member A8 |
| 1569787_at | RFTN1 | 3.67E-02 | 1.33 | raftlin, lipid raft linker 1 |

***TABLE S5*** *Probe sets significantly (pFDR<0.05) differentially expressed in whole blood between male and female patients (primary analysis; baseline samples). pFDR, adjusted p-value*

| Probe set ID | Gene Symbol | pFDR value | Fold Change | Gene title |
| --- | --- | --- | --- | --- |
| 201909_at | RPS4Y1 | 9.91E-60 | 194.22 | ribosomal protein S4 Y-linked 1 |
| 206700_s_at | KDM5D | 2.72E-55 | 164.46 | lysine demethylase 5D |
| 214131_at | TXLNGY | 1.05E-48 | 58.15 | taxilin gamma pseudogene, Y-linked |
| 205000_at | DDX3Y | 1.28E-48 | 81.65 | DEAD-box helicase 3 Y-linked |
| 204409_s_at | EIF1AY | 1.56E-48 | 212.65 | eukaryotic translation initiation factor 1A Y-linked |
| 244482_at | --- | 2.60E-44 | 38.74 | --- |
| 205001_s_at | DDX3Y | 6.02E-43 | 18.23 | DEAD-box helicase 3 Y-linked |
| 232618_at | TXLNGY | 3.84E-40 | 42.59 | taxilin gamma pseudogene, Y-linked |
| 228492_at | USP9Y | 9.88E-40 | 68.24 | ubiquitin specific peptidase 9 Y-linked |
| 211149_at | UTY | 1.42E-37 | 37.07 | ubiquitously transcribed tetratricopeptide repeat containing, Y-linked |
| 204410_at | EIF1AY | 1.62E-37 | 54.55 | eukaryotic translation initiation factor 1A Y-linked |
| 224293_at | TTTY10 | 5.10E-36 | 11.03 | testis-specific transcript, Y-linked 10 |
| 207063_at | TTTY14 | 2.63E-35 | 12.34 | testis-specific transcript, Y-linked 14 |
| 223646_s_at | TXLNGY | 3.69E-35 | 35.23 | taxilin gamma pseudogene, Y-linked |
| 223645_s_at | TXLNGY | 2.79E-34 | 25.95 | taxilin gamma pseudogene, Y-linked |
| 206279_at | PRKY | 3.51E-34 | 8.79 | protein kinase Y-linked (pseudogene) |
| 208067_x_at | UTY | 5.57E-32 | 5.04 | ubiquitously transcribed tetratricopeptide repeat containing, Y-linked |
| 227671_at | XIST | 5.29E-29 | -419.71 | X inactive specific transcript |
| 230760_at | ZFY | 1.10E-28 | 23.32 | zinc finger protein Y-linked |
| 224588_at | XIST | 1.24E-28 | -268.66 | X inactive specific transcript |
| 235446_at | XIST | 1.52E-28 | -118.89 | X inactive specific transcript |
| 231592_at | XIST /// TSIX | 1.52E-28 | -185.95 | X inactive specific transcript /// TSIX transcript, XIST antisense RNA |
| 224590_at | XIST | 1.70E-28 | -317.05 | X inactive specific transcript |
| 221728_x_at | XIST | 2.01E-28 | -352.63 | X inactive specific transcript |
| 214218_s_at | XIST | 2.74E-28 | -273.00 | X inactive specific transcript |
| 236694_at | TXLNGY | 7.62E-28 | 11.00 | taxilin gamma pseudogene, Y-linked |
| 214983_at | TTTY15 | 1.09E-27 | 6.47 | testis-specific transcript, Y-linked 15 |
| 1556677_at | --- | 3.80E-26 | 8.23 | --- |
| 224589_at | XIST | 5.01E-25 | -12.81 | X inactive specific transcript |
| 210322_x_at | UTY | 8.27E-24 | 4.04 | ubiquitously transcribed tetratricopeptide repeat containing, Y-linked |
| 206624_at | USP9Y | 5.16E-23 | 7.98 | ubiquitin specific peptidase 9 Y-linked |
| 1560800_at | --- | 9.10E-22 | 7.03 | --- |
| 228919_at | --- | 5.08E-19 | -9.70 | --- |
| 237225_at | --- | 2.23E-15 | 3.34 | --- |
| 207617_at | DDX3X | 7.24E-15 | -3.10 | DEAD-box helicase 3 X-linked |
| 239677_at | --- | 3.57E-14 | 2.46 | --- |
| 228262_at | MAP7D2 | 2.42E-13 | -2.93 | MAP7 domain containing 2 |
| 1570360_s_at | DDX3Y | 3.72E-13 | 1.93 | DEAD-box helicase 3 Y-linked |
| 1562313_at | BCORP1 | 7.35E-12 | 2.38 | BCL6 corepressor pseudogene 1 |
| 243712_at | XIST | 1.25E-09 | -1.68 | X inactive specific transcript |
| 1566854_at | --- | 1.83E-09 | 1.95 | --- |
| 201019_s_at | EIF1AX | 3.40E-09 | -1.49 | eukaryotic translation initiation factor 1A X-linked |
| 205206_at | ANOS1 | 4.85E-09 | 2.83 | anosmin 1 |
| 206769_at | TMSB4Y | 6.37E-08 | 1.36 | thymosin beta 4 Y-linked |
| 240438_at | --- | 6.99E-08 | -1.50 | --- |
| 204061_at | PRKX | 6.99E-08 | -1.56 | protein kinase X-linked |
| 232974_at | --- | 7.24E-08 | -1.56 | --- |
| 201017_at | EIF1AX | 1.22E-07 | -1.65 | eukaryotic translation initiation factor 1A X-linked |
| 222031_at | LOC389906 | 1.36E-07 | -1.89 | zinc finger protein 839 pseudogene |
| 235942_at | FAM224B /// FAM224A | 2.02E-07 | 3.57 | family with sequence similarity 224 member B /// family with sequence similarity 224 member A |
| 214678_x_at | ZFX | 2.09E-07 | -1.48 | zinc finger protein X-linked |
| 237908_at | --- | 2.24E-07 | -1.85 | --- |
| 1564510_at | --- | 3.44E-07 | 1.87 | --- |
| 201018_at | EIF1AX | 4.95E-07 | -1.62 | eukaryotic translation initiation factor 1A X-linked |
| 227520_at | TXLNG | 9.98E-07 | -1.47 | taxilin gamma |
| 201016_at | EIF1AX | 3.59E-06 | -1.71 | eukaryotic translation initiation factor 1A X-linked |
| 1564232_at | --- | 7.36E-06 | -1.94 | --- |
| 1556102_x_at | LOC389906 | 7.36E-06 | -1.48 | zinc finger protein 839 pseudogene |
| 203974_at | PUDP | 8.00E-06 | -1.31 | pseudouridine 5'-phosphatase |
| 1564639_at | --- | 9.75E-06 | -1.96 | --- |
| 1560395_at | --- | 1.18E-05 | 4.25 | --- |
| 212514_x_at | DDX3X | 1.57E-05 | -1.33 | DEAD-box helicase 3 X-linked |
| 229315_at | --- | 2.80E-05 | -1.70 | --- |
| 1557954_at | TXLNG | 3.71E-05 | -1.49 | taxilin gamma |
| 213666_at | SEPTIN6 | 5.14E-05 | -1.36 | septin 6 |
| 203992_s_at | KDM6A | 8.38E-05 | -1.46 | lysine demethylase 6A |
| 209739_s_at | PNPLA4 | 8.38E-05 | -1.33 | patatin like phospholipase domain containing 4 |
| 203903_s_at | HEPH | 8.86E-05 | -2.23 | hephaestin |
| 227575_s_at | NRDE2 | 8.99E-05 | 1.31 | NRDE-2, necessary for RNA interference, domain containing |
| 224936_at | EIF2S3 | 1.91E-04 | -1.17 | eukaryotic translation initiation factor 2 subunit gamma |
| 229022_at | ZFX | 2.95E-04 | -1.40 | zinc finger protein X-linked |
| 200933_x_at | RPS4X | 3.31E-04 | -1.12 | ribosomal protein S4 X-linked |
| 201589_at | SMC1A | 3.41E-04 | -1.21 | structural maintenance of chromosomes 1A |
| 201211_s_at | DDX3X | 3.77E-04 | -1.40 | DEAD-box helicase 3 X-linked |
| 239207_at | KDM5C | 5.65E-04 | -1.30 | lysine demethylase 5C |
| 203991_s_at | KDM6A | 7.59E-04 | -1.48 | lysine demethylase 6A |
| 1554448_at | JPX | 9.96E-04 | -1.35 | JPX transcript, XIST activator |
| 238194_at | DGKK | 1.04E-03 | 2.16 | diacylglycerol kinase kappa |
| 213347_x_at | RPS4X | 1.23E-03 | -1.10 | ribosomal protein S4 X-linked |
| 212413_at | SEPTIN6 | 1.51E-03 | -1.34 | septin 6 |
| 214298_x_at | SEPTIN6 | 1.70E-03 | -1.27 | septin 6 |
| 216342_x_at | --- | 1.72E-03 | -1.23 | --- |
| 212414_s_at | SEPTIN6 /// GLYR1 | 2.80E-03 | -1.23 | septin 6 /// glyoxylate reductase 1 homolog |
| 1558046_x_at | LOC389906 | 2.80E-03 | -1.47 | zinc finger protein 839 pseudogene |
| 207920_x_at | ZFX | 3.63E-03 | -1.47 | zinc finger protein X-linked |
| 213876_x_at | ZRSR2 | 4.29E-03 | -1.22 | zinc finger CCCH-type, RNA binding motif and serine/arginine rich 2 |
| 219969_at | TXLNG | 5.38E-03 | -1.32 | taxilin gamma |
| 205321_at | EIF2S3 | 5.40E-03 | -1.26 | eukaryotic translation initiation factor 2 subunit gamma |
| 1558045_a_at | LOC389906 | 5.97E-03 | -1.47 | zinc finger protein 839 pseudogene |
| 215230_x_at | EIF3C /// EIF3CL | 6.84E-03 | 1.28 | eukaryotic translation initiation factor 3 subunit C /// eukaryotic translation initiation factor 3 subunit C like |
| 208174_x_at | ZRSR2 | 6.84E-03 | -1.22 | zinc finger CCCH-type, RNA binding motif and serine/arginine rich 2 |
| 217019_at | --- | 7.10E-03 | -1.31 | --- |
| 208577_at | HIST1H3C | 7.33E-03 | 1.64 | histone cluster 1 H3 family member c |
| 1558237_x_at | DR1 | 9.03E-03 | 1.25 | down-regulator of transcription 1 |
| 201689_s_at | TPD52 | 9.99E-03 | -1.43 | tumor protein D52 |
| 1554447_at | JPX | 1.26E-02 | -1.45 | JPX transcript, XIST activator |
| 230349_at | XKRX | 1.26E-02 | -1.42 | XK related X-linked |
| 217818_s_at | ARPC4 | 1.26E-02 | 1.22 | actin related protein 2/3 complex subunit 4 |
| 200647_x_at | EIF3C /// EIF3CL | 1.31E-02 | 1.28 | eukaryotic translation initiation factor 3 subunit C /// eukaryotic translation initiation factor 3 subunit C like |
| 229808_at | CHAF1A | 1.48E-02 | 1.27 | chromatin assembly factor 1 subunit A |
| 204375_at | CLSTN3 | 1.58E-02 | 1.26 | calsyntenin 3 |
| 241677_x_at | --- | 1.90E-02 | -1.18 | --- |
| 235825_at | LOC101927330 | 2.02E-02 | 1.31 | uncharacterized LOC101927330 |
| 209531_at | GSTZ1 | 2.18E-02 | 1.26 | glutathione S-transferase zeta 1 |
| 223473_at | MPV17L2 | 2.77E-02 | 1.17 | MPV17 mitochondrial inner membrane protein like 2 |
| 207194_s_at | ICAM4 | 2.78E-02 | 1.55 | intercellular adhesion molecule 4 (Landsteiner-Wiener blood group) |
| 1557051_s_at | HOTAIRM1 | 2.83E-02 | 1.52 | HOXA transcript antisense RNA, myeloid-specific 1 |
| 211672_s_at | ARPC4 /// ARPC4-TTLL3 | 2.90E-02 | 1.19 | actin related protein 2/3 complex subunit 4 /// ARPC4-TTLL3 readthrough |
| 210949_s_at | EIF3C /// EIF3CL | 2.92E-02 | 1.27 | eukaryotic translation initiation factor 3 subunit C /// eukaryotic translation initiation factor 3 subunit C like |
| 1557260_a_at | ZNF382 | 2.92E-02 | -1.52 | zinc finger protein 382 |
| 1564150_a_at | LINC01619 | 3.14E-02 | -1.45 | long intergenic non-protein coding RNA 1619 |
| 224935_at | EIF2S3 | 3.21E-02 | -1.12 | eukaryotic translation initiation factor 2 subunit gamma |
| 1563453_at | --- | 3.32E-02 | 1.39 | --- |
| 207247_s_at | ZFY /// ZFX | 3.40E-02 | -1.61 | zinc finger protein Y-linked /// zinc finger protein X-linked |
| 217107_at | --- | 3.40E-02 | -1.18 | --- |
| 210835_s_at | CTBP2 | 3.46E-02 | 1.21 | C-terminal binding protein 2 |
| 205584_at | ALG13 | 3.46E-02 | -1.36 | ALG13 UDP-N-acetylglucosaminyltransferase subunit |
| 204986_s_at | TAOK2 | 3.77E-02 | 1.30 | TAO kinase 2 |
| 215064_at | SC5D | 3.97E-02 | -1.36 | sterol-C5-desaturase |
| 230546_at | VASH1 | 4.01E-02 | 1.32 | vasohibin 1 |
| 221234_s_at | BACH2 | 4.17E-02 | -1.53 | BTB domain and CNC homolog 2 |
| 227388_at | TUSC1 | 4.17E-02 | -1.51 | tumor suppressor candidate 1 |
| 204608_at | ASL | 4.21E-02 | 1.31 | argininosuccinate lyase |
| 1560695_at | QTRT2 | 4.21E-02 | 1.16 | queuine tRNA-ribosyltransferase accessory subunit 2 |
| 226710_at | C8orf82 | 4.21E-02 | 1.23 | chromosome 8 open reading frame 82 |
| 219557_s_at | NRIP3 | 4.27E-02 | 1.34 | nuclear receptor interacting protein 3 |
| 205259_at | NR3C2 | 4.28E-02 | -1.42 | nuclear receptor subfamily 3 group C member 2 |
| 212736_at | BMERB1 | 4.40E-02 | 1.20 | bMERB domain containing 1 |
| 229149_at | HOTAIRM1 | 4.57E-02 | 1.58 | HOXA transcript antisense RNA, myeloid-specific 1 |

***TABLE S6*** *Significant Gene Ontology (GO) biological processes (BP), GO molecular functions (MF), GO cellular components (CC), Reactome and Wiki pathways identified by functional enrichment analysis of the significant (pFDR<0.05) differentially expressed genes (DEGs) in whole blood between males and females patients (primary analysis; baseline samples). *Molecules from the Gene list that are annotated to the functional group. pFDR, adjusted p-value*

| Entity | Description | | pFDR | *Genes |
| --- | --- | --- | --- | --- |
| GO:BP | protein dealkylation | 5.28E-04 | | KDM5D,UTY,KDM6A,KDM5C |
| GO:BP | histone demethylation | 5.28E-04 | | KDM5D,UTY,KDM6A,KDM5C |
| GO:BP | histone lysine demethylation | 5.28E-04 | | KDM5D,UTY,KDM6A,KDM5C |
| GO:BP | protein demethylation | 5.28E-04 | | KDM5D,UTY,KDM6A,KDM5C |
| GO:BP | translational initiation | 5.28E-04 | | RPS4Y1,EIF1AY,DDX3X,EIF1AX,EIF2S3,RPS4X,EIF3C |
| GO:BP | positive regulation of translation | 7.74E-03 | | DDX3X,EIF2S3,RPS4X,EIF3C,MPV17L2 |
| GO:BP | demethylation | 9.24E-03 | | KDM5D,UTY,KDM6A,KDM5C |
| GO:BP | histone H3-K27 demethylation | 1.08E-02 | | UTY,KDM6A |
| GO:BP | histone H3-K4 demethylation, trimethyl-H3-K4-specific | 1.08E-02 | | KDM5D,KDM5C |
| GO:BP | positive regulation of cellular amide metabolic process | 1.11E-02 | | DDX3X,EIF2S3,RPS4X,EIF3C,MPV17L2 |
| GO:BP | amide biosynthetic process | 2.07E-02 | | RPS4Y1,EIF1AY,DDX3X,EIF1AX,EIF2S3,RPS4X,EIF3C,GSTZ1,MPV17L2,ASL |
| GO:BP | histone H3-K4 demethylation | 2.88E-02 | | KDM5D,KDM5C |
| GO:MF | histone demethylase activity | 2.38E-04 | | KDM5D,UTY,KDM6A,KDM5C |
| GO:MF | demethylase activity | 4.04E-04 | | KDM5D,UTY,KDM6A,KDM5C |
| GO:MF | translation initiation factor activity | 1.16E-03 | | EIF1AY,EIF1AX,EIF2S3,EIF3C |
| GO:MF | histone demethylase activity (H3-trimethyl-K4 specific) | 2.49E-03 | | KDM5D,KDM5C |
| GO:MF | histone demethylase activity (H3-K27 specific) | 3.32E-03 | | UTY,KDM6A |
| GO:MF | dioxygenase activity | 4.31E-03 | | KDM5D,UTY,KDM6A,KDM5C |
| GO:MF | translation factor activity, RNA binding | 4.52E-03 | | EIF1AY,EIF1AX,EIF2S3,EIF3C |
| GO:MF | cAMP-dependent protein kinase activity | 5.14E-03 | | PRKY,PRKX |
| GO:MF | histone demethylase activity (H3-K4 specific) | 5.14E-03 | | KDM5D,KDM5C |
| GO:MF | cyclic nucleotide-dependent protein kinase activity | 6.73E-03 | | PRKY,PRKX |
| GO:MF | translation regulator activity, nucleic acid binding | 6.73E-03 | | EIF1AY,EIF1AX,EIF2S3,EIF3C |
| GO:MF | translation regulator activity | 1.27E-02 | | EIF1AY,EIF1AX,EIF2S3,EIF3C |
| GO:MF | heterocyclic compound binding | 1.27E-02 | | RPS4Y1,KDM5D,DDX3Y,EIF1AY,UTY,PRKY,ZFY,DDX3X,EIF1AX,PRKX,ZFX,SEPTIN6,KDM6A,EIF2S3,RPS4X,SMC1A,KDM5C,DGKK,ZRSR2,EIF3C,H3C3,DR1,ZNF382,CTBP2,ALG13,TAOK2,BACH2,NR3C2 |
| GO:MF | nucleic acid binding | 1.33E-02 | | RPS4Y1,KDM5D,DDX3Y,EIF1AY,UTY,ZFY,DDX3X,EIF1AX,ZFX,KDM6A,EIF2S3,RPS4X,SMC1A,KDM5C,ZRSR2,EIF3C,H3C3,DR1,ZNF382,ALG13,BACH2,NR3C2 |
| GO:MF | oxidoreductase activity | 1.33E-02 | | KDM5D,UTY,KDM6A,HEPH,KDM5C,GSTZ1,CTBP2,SC5D |
| GO:MF | organic cyclic compound binding | 1.33E-02 | | RPS4Y1,KDM5D,DDX3Y,EIF1AY,UTY,PRKY,ZFY,DDX3X,EIF1AX,PRKX,ZFX,SEPTIN6,KDM6A,EIF2S3,RPS4X,SMC1A,KDM5C,DGKK,ZRSR2,EIF3C,H3C3,DR1,ZNF382,CTBP2,ALG13,TAOK2,BACH2,NR3C2 |
| GO:MF | pseudouridine 5'-phosphatase activity | 3.39E-02 | | PUDP |
| GO:MF | CTPase activity | 3.39E-02 | | DDX3X |
| GO:MF | maleylacetoacetate isomerase activity | 3.39E-02 | | GSTZ1 |
| GO:MF | translation initiation factor binding | 4.43E-02 | | DDX3X,EIF3C |
| GO:CC | histone methyltransferase complex | 1.59E-02 | | KDM5D,UTY,KDM6A,KDM5C |
| GO:CC | cAMP-dependent protein kinase complex | 1.59E-02 | | PRKY,PRKX |
| GO:CC | methyltransferase complex | 1.59E-02 | | KDM5D,UTY,KDM6A,KDM5C |
| GO:CC | cytosolic small ribosomal subunit | 1.59E-02 | | RPS4Y1,DDX3X,RPS4X |
| GO:CC | eukaryotic translation initiation factor 3 complex | 2.38E-02 | | DDX3X,EIF3C |
| GO:CC | MLL3/4 complex | 2.38E-02 | | UTY,KDM6A |
| GO:CC | small ribosomal subunit | 2.38E-02 | | RPS4Y1,DDX3X,RPS4X |
| GO:CC | pole plasm | 2.38E-02 | | DDX3Y,DDX3X |
| GO:CC | germ plasm | 2.38E-02 | | DDX3Y,DDX3X |
| GO:CC | P granule | 2.38E-02 | | DDX3Y,DDX3X |
| GO:CC | intracellular non-membrane-bounded organelle | 2.38E-02 | | RPS4Y1,KDM5D,DDX3Y,UTY,ZFY,DDX3X,MAP7D2,TMSB4Y,ZFX,SEPTIN6,KDM6A,PNPLA4,NRDE2,RPS4X,SMC1A,H3C3,ARPC4,CHAF1A,MPV17L2,TAOK2,BACH2,NR3C2,BMERB1 |
| GO:CC | ribosomal subunit | 2.38E-02 | | RPS4Y1,DDX3X,RPS4X,MPV17L2 |
| GO:CC | non-membrane-bounded organelle | 2.38E-02 | | RPS4Y1,KDM5D,DDX3Y,UTY,ZFY,DDX3X,MAP7D2,TMSB4Y,ZFX,SEPTIN6,KDM6A,PNPLA4,NRDE2,RPS4X,SMC1A,H3C3,ARPC4,CHAF1A,MPV17L2,TAOK2,BACH2,NR3C2,BMERB1 |
| GO:CC | nucleoplasm | 2.38E-02 | | RPS4Y1,KDM5D,DDX3Y,UTY,ZFY,DDX3X,PRKX,ZFX,KDM6A,NRDE2,RPS4X,SMC1A,KDM5C,ZRSR2,H3C3,DR1,ZNF382,TAOK2,BACH2,NR3C2 |
| GO:CC | septin collar | 3.12E-02 | | SEPTIN6 |
| GO:CC | negative cofactor 2 complex | 3.12E-02 | | DR1 |
| GO:CC | protein-containing complex | 3.12E-02 | | RPS4Y1,KDM5D,UTY,PRKY,DDX3X,PRKX,SEPTIN6,KDM6A,EIF2S3,RPS4X,SMC1A,KDM5C,ZRSR2,EIF3C,H3C3,DR1,ARPC4,CHAF1A,CLSTN3,MPV17L2,CTBP2,TAOK2,QTRT2,NR3C2 |
| GO:CC | intracellular | 3.12E-02 | | RPS4Y1,KDM5D,DDX3Y,USP9Y,UTY,PRKY,ZFY,DDX3X,MAP7D2,EIF1AX,TMSB4Y,PRKX,ZFX,TXLNG,PUDP,SEPTIN6,KDM6A,PNPLA4,HEPH,NRDE2,EIF2S3,RPS4X,SMC1A,KDM5C,DGKK,ZRSR2,EIF3C,H3C3,DR1,TPD52,ARPC4,CHAF1A,CLSTN3,GSTZ1,MPV17L2,ZNF382,CTBP2,ALG13,TAOK2,SC5D,VASH1,BACH2,ASL,QTRT2,NR3C2,BMERB1 |
| GO:CC | cytosolic ribosome | 3.16E-02 | | RPS4Y1,DDX3X,RPS4X |
| GO:CC | ribosome | 4.02E-02 | | RPS4Y1,DDX3X,RPS4X,MPV17L2 |
| REACTOME | Formation of the ternary complex, and subsequently, the 43S complex | 3.13E-05 | | RPS4Y1,EIF1AX,EIF2S3,RPS4X,EIF3C |
| REACTOME | Translation initiation complex formation | 3.13E-05 | | RPS4Y1,EIF1AX,EIF2S3,RPS4X,EIF3C |
| REACTOME | Ribosomal scanning and start codon recognition | 3.13E-05 | | RPS4Y1,EIF1AX,EIF2S3,RPS4X,EIF3C |
| REACTOME | HDMs demethylate histones | 3.13E-05 | | KDM5D,UTY,KDM6A,KDM5C,H3C3 |
| REACTOME | Activation of the mRNA upon binding of the cap-binding complex and eIFs, and subsequent binding to 43S | 3.13E-05 | | RPS4Y1,EIF1AX,EIF2S3,RPS4X,EIF3C |
| REACTOME | L13a-mediated translational silencing of Ceruloplasmin expression | 5.16E-04 | | RPS4Y1,EIF1AX,EIF2S3,RPS4X,EIF3C |
| REACTOME | GTP hydrolysis and joining of the 60S ribosomal subunit | 5.16E-04 | | RPS4Y1,EIF1AX,EIF2S3,RPS4X,EIF3C |
| REACTOME | Cap-dependent Translation Initiation | 5.37E-04 | | RPS4Y1,EIF1AX,EIF2S3,RPS4X,EIF3C |
| REACTOME | Eukaryotic Translation Initiation | 5.37E-04 | | RPS4Y1,EIF1AX,EIF2S3,RPS4X,EIF3C |
| REACTOME | Chromatin organization | 2.31E-03 | | KDM5D,UTY,KDM6A,KDM5C,H3C3,DR1 |
| REACTOME | Chromatin modifying enzymes | 2.31E-03 | | KDM5D,UTY,KDM6A,KDM5C,H3C3,DR1 |
| REACTOME | Formation of a pool of free 40S subunits | 3.65E-03 | | RPS4Y1,EIF1AX,RPS4X,EIF3C |
| REACTOME | Translation | 2.34E-02 | | RPS4Y1,EIF1AX,EIF2S3,RPS4X,EIF3C |
| WIKI | Translation Factors | 1.20E-03 | | EIF1AY,EIF1AX,EIF2S3,EIF3C |

***FIGURE S3*** *Differential expression analysis in sputum cells between eosinophil^high^ and eosinophil^low^ patients (validation analysis; placebo samples). Volcano plot depicting all detected probe sets and coloured by fold change (FC) and adjusted p-value (pFDR): green, FC >|1.3| and pFDR <0.05; red, pFDR<0.05; orange, FC>|1.3 |. Abbreviations: FC, fold-change; pFDR, adjusted p-value*


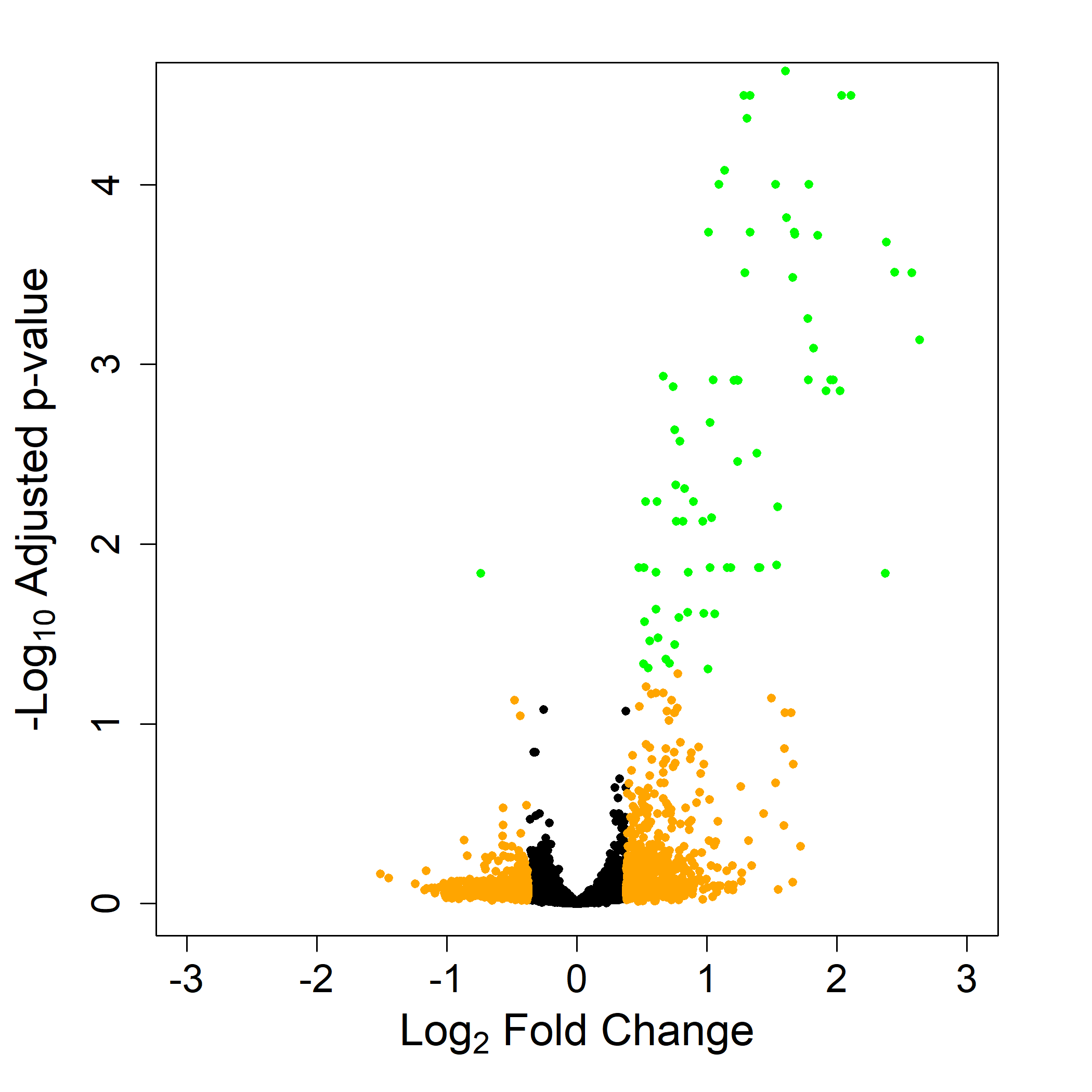


***FIGURE S4*** *Heatmap with z-score representation and hierarchical clustering of patients based on values of the differentially (pFDR<0.05) expressed probe sets in sputum cells (validation analysis; placebo samples).*


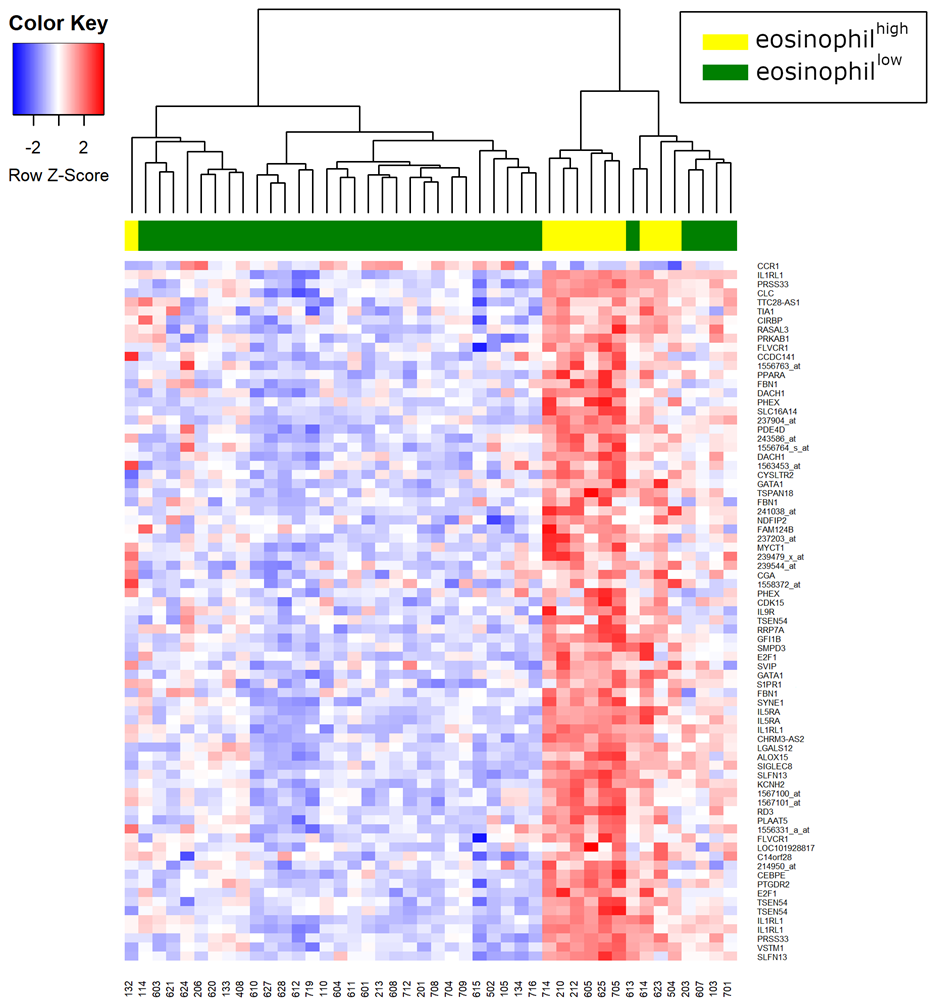


***TABLE S7*** *Significant Gene Ontology (GO) biological processes (BP), GO molecular functions (MF) and Wiki pathways identified by functional enrichment analysis of the significant (pFDR<0.05) differentially expressed genes (DEGs) in sputum cells between eosinophil^high^ and eosinophil^low^ patients (validation analysis; placebo samples). *Molecules from the Gene list that are annotated to the functional group. pFDR, adjusted p-value*

| Entity | Description | pFDR | *Genes |
| --- | --- | --- | --- |
| GO:BP | biomineralization | 6.05E-05 | PHEX,GATA1,SMPD3,PPARA,ALOX15,CCR1,S1PR1 |
| GO:BP | biomineral tissue development | 6.05E-05 | PHEX,GATA1,SMPD3,PPARA,ALOX15,CCR1,S1PR1 |
| GO:BP | bone mineralization | 1.09E-04 | PHEX,GATA1,SMPD3,ALOX15,CCR1,S1PR1 |
| GO:BP | regulation of interleukin-5 production | 5.67E-03 | IL5RA,IL1RL1,PDE4D |
| GO:BP | interleukin-5 production | 5.67E-03 | IL5RA,IL1RL1,PDE4D |
| GO:BP | immune system process | 2.37E-02 | SLFN13,IL5RA,CEBPE,IL1RL1,VSTM1,GATA1,SMPD3,SVIP,PTGDR2,PDE4D,RASAL3,FBN1,FLVCR1,CYSLTR2,ALOX15,CLC,CCR1,S1PR1 |
| GO:BP | organ growth | 2.50E-02 | SMPD3,CGA,FLVCR1,PPARA,S1PR1 |
| GO:BP | regulation of heart rate by chemical signal | 3.10E-02 | KCNH2,PDE4D |
| GO:BP | cellular response to organic substance | 3.84E-02 | E2F1,IL5RA,PHEX,CEBPE,IL1RL1,GATA1,SMPD3,PDE4D,CGA,FBN1,PPARA,ALOX15,CCR1,S1PR1,IL9R,TIA1 |
| GO:BP | ossification | 4.42E-02 | PHEX,GATA1,SMPD3,ALOX15,CCR1,S1PR1 |
| GO:BP | G protein-coupled receptor signaling pathway, coupled to cyclic nucleotide second messenger | 4.42E-02 | PTGDR2,PDE4D,CYSLTR2,CCR1,S1PR1 |
| GO:BP | response to stimulus | 4.74E-02 | E2F1,SLFN13,IL5RA,PHEX,CEBPE,RD3,IL1RL1,PRSS33,VSTM1,GATA1,DACH1,KCNH2,SIGLEC8,LGALS12,SMPD3,SVIP,PTGDR2,PDE4D,PRKAB1,CGA,CIRBP,RASAL3,FBN1,CYSLTR2,PPARA,ALOX15,CLC,CCR1,NDFIP2,S1PR1,IL9R,TIA1 |
| GO:BP | sphingolipid mediated signaling pathway | 4.74E-02 | SMPD3,S1PR1 |
| GO:BP | positive regulation of interleukin-5 production | 4.90E-02 | IL1RL1,PDE4D |
| GO:MF | cytokine receptor activity | 2.29E-02 | IL5RA,IL1RL1,CCR1,IL9R |
| GO:MF | immune receptor activity | 3.37E-02 | IL5RA,IL1RL1,CCR1,IL9R |
| WP | Eicosanoid metabolism via Lipo Oxygenases (LOX) | 4.78E-03 | CYSLTR2,PPARA,ALOX15 |
| WP | Human Thyroid Stimulating Hormone (TSH) signaling pathway | 2.81E-02 | E2F1,PDE4D,CGA |

**TABLE S8** *Fold change and adjusted p-values (pFDR) of the primary and validation analyses for significant (pFDR<0.05) differentially expressed genes (DEGs) identified in the two analyses. pFDR, adjusted p-value*

| Probe_set ID | Gene Symbol | Fold change primary analysis | Fold change validation analysis | pFDR  primary anaysis | pFDR  validation analysis |
| --- | --- | --- | --- | --- | --- |
| 1552348_at | PRSS33^‡^ | 3.8 | 5.44 | 9.47E-03 | 3.08E-04 |
| 1552875_a_at | CD200R1^†^ | 1.45 | 1.13 | 1.41E-02 | 9.30E-01 |
| 1552908_at | GCSAML^†^ | 2.39 | 1.57 | 9.47E-03 | 8.18E-01 |
| 1553423_a_at | SLFN13^†‡^ | 2.15 | 2.88 | 8.62E-03 | 9.94E-05 |
| 1554717_a_at | PDE4D^†‡^ | 2.58 | 2.61 | 9.47E-03 | 3.11E-03 |
| 1557733_a_at | CHRM3-AS2^‡^ | 3.52 | 4.06 | 9.47E-03 | 1.40E-03 |
| 202933_s_at | YES1^†^ | 1.69 | 1.48 | 3.53E-02 | 7.97E-01 |
| 202976_s_at | RHOBTB3 | 1.77 | 1.4 | 1.55E-02 | 7.86E-01 |
| 203222_s_at | TLE1 | 1.71 | 1.66 | 2.39E-02 | 3.48E-01 |
| 203373_at | SOCS2 | 1.84 | 1.66 | 4.19E-02 | 3.46E-01 |
| 204722_at | SCN3B | 1.78 | 1.09 | 3.58E-02 | 9.51E-01 |
| 205471_s_at | DACH1^‡^ | 2.12 | 2.34 | 4.00E-02 | 1.22E-03 |
| 206207_at | CLC^†‡^ | 4.2 | 5.17 | 1.48E-02 | 1.46E-02 |
| 207067_s_at | HDC | 3.38 | 2.49 | 1.11E-02 | 4.44E-01 |
| 207328_at | ALOX15^†‡^ | 3.13 | 2.9 | 1.77E-02 | 1.30E-02 |
| 207538_at | IL4^†^ | 1.75 | 1.37 | 1.48E-02 | 4.72E-01 |
| 208253_at | SIGLEC8^‡^ | 3.97 | 3.92 | 2.90E-03 | 1.22E-03 |
| 208605_s_at | NTRK1^†^ | 1.39 | 1.15 | 4.49E-02 | 8.38E-01 |
| 208650_s_at | CD24^†^ | 3.44 | 3.29 | 1.77E-02 | 4.81E-01 |
| 210036_s_at | KCNH2^‡^ | 2.81 | 3.43 | 3.04E-03 | 1.22E-03 |
| 210446_at | GATA1^†‡^ | 1.64 | 1.58 | 1.41E-02 | 1.16E-03 |
| 211517_s_at | IL5RA^†‡^ | 2.81 | 3.16 | 1.02E-02 | 3.28E-04 |
| 215024_at | CCZ1B | 1.44 | 1.12 | 3.84E-02 | 9.15E-01 |
| 218847_at | IGF2BP2 | 1.54 | 1.08 | 3.80E-02 | 9.38E-01 |
| 219695_at | SMPD3^†‡^ | 2.03 | 2.03 | 8.62E-03 | 2.10E-03 |
| 220059_at | STAP1^†^ | 2.96 | 1.75 | 1.03E-02 | 7.97E-01 |
| 222906_at | FLVCR1^†‡^ | 2.09 | 2.08 | 1.85E-02 | 2.44E-02 |
| 223710_at | CCL26^†^ | 2.7 | 2.12 | 9.54E-03 | 3.48E-01 |
| 223805_at | OSBPL6 | 1.8 | 1.32 | 4.04E-02 | 8.15E-01 |
| 223828_s_at | LGALS12^‡^ | 3.4 | 3.86 | 1.85E-02 | 1.22E-03 |
| 224801_at | NDFIP2^‡^ | 1.87 | 1.8 | 4.20E-02 | 2.39E-02 |
| 227307_at | TSPAN18^‡^ | 1.79 | 2.07 | 3.84E-02 | 1.22E-03 |
| 231050_at | PLAAT5^‡^ | 2.23 | 2.31 | 2.05E-02 | 1.23E-03 |
| 231947_at | MYCT1^‡^ | 2.34 | 2.52 | 6.48E-05 | 3.19E-05 |
| 232027_at | SYNE1^‡^ | 3.08 | 4.1 | 9.47E-03 | 3.19E-05 |
| 235818_at | VSTM1^†‡^ | 3.02 | 3.53 | 9.47E-03 | 8.13E-04 |
| 237403_at | GFI1B^‡^ | 2.12 | 2.43 | 9.47E-03 | 3.19E-05 |
| 238029_s_at | SLC16A14^‡^ | 2.57 | 4.3 | 1.55E-02 | 3.19E-05 |
| 239229_at | PHEX^‡^ | 2.34 | 3.18 | 1.02E-02 | 1.84E-04 |
| 239401_at | S1PR1^†‡^ | 1.64 | 1.54 | 4.04E-02 | 3.31E-02 |
| 242809_at | IL1RL1^†‡^ | 4.88 | 6.2 | 1.02E-02 | 7.30E-04 |
| 204947_at | E2F1 | 1.4 | 2.19 | 5.61E-01 | 8.31E-05 |
| 225879_at | TSEN54 | 1.12 | 2.13 | 7.87E-01 | 9.94E-05 |
| 236563_at | RD3 | 1.69 | 2.51 | 6.41E-02 | 1.84E-04 |
| 214523_at | CEBPE^†^ | 1.65 | 2.02 | 1.06E-01 | 1.84E-04 |
| 230285_at | SVIP^†^ | 1.49 | 1.68 | 7.22E-02 | 2.32E-03 |
| 206361_at | PTGDR2^†^ | 1.51 | 1.73 | 3.47E-01 | 2.68E-03 |
| 201834_at | PRKAB1 | 1.28 | 1.69 | 4.03E-01 | 4.67E-03 |
| 204637_at | CGA | 1.52 | 1.77 | 2.76E-01 | 4.90E-03 |
| 228677_s_at | RASAL3^†^ | 1.31 | 1.53 | 5.03E-01 | 5.79E-03 |
| 230142_s_at | CIRBP | 1.24 | 1.86 | 7.30E-01 | 5.79E-03 |
| 202766_s_at | FBN1^†^ | 1.79 | 2.91 | 5.51E-01 | 6.21E-03 |
| 220813_at | CYSLTR2^†^ | 1.73 | 1.76 | 5.04E-02 | 7.46E-03 |
| 237142_at | PPARA | 1.32 | 1.7 | 3.92E-01 | 7.46E-03 |
| 1556003_a_at | LOC101928817 | 1.5 | 2.22 | 5.31E-01 | 1.35E-02 |
| 202938_x_at | RRP7A | 1.27 | 1.39 | 5.34E-01 | 1.35E-02 |
| 1553645_at | CCDC141 | 1.69 | 1.52 | 2.59E-01 | 1.44E-02 |
| 205099_s_at | CCR1^†^ | -1.11 | -1.67 | 8.57E-01 | 1.46E-02 |
| 220637_at | FAM124B | 1.72 | 1.96 | 2.04E-01 | 2.42E-02 |
| 239201_at | CDK15 | 1.16 | 1.43 | 7.06E-01 | 2.70E-02 |
| 235369_at | C14orf28 | 1.17 | 1.6 | 7.30E-01 | 4.37E-02 |
| 244189_at | TTC28-AS1 | 1.34 | 1.64 | 4.00E-01 | 4.60E-02 |
| 208164_s_at | IL9R | 1.17 | 1.43 | 6.65E-01 | 4.63E-02 |
| 1554890_a_at | TIA1 | 1.42 | 1.46 | 5.82E-02 | 4.88E-02 |

† Inflammatory genes annotated in immune system Gene Ontology (GO) biological processes. pFDR, adjusted p-value

‡ Differentially expressed genes (DEGs) in common between the primary and validation analyses
